# Effectiveness of a personalised self-management intervention for people living with long covid (Listen trial): pragmatic, multicentre, parallel group, randomised controlled trial

**DOI:** 10.1136/bmjmed-2024-001068

**Published:** 2025-01-31

**Authors:** Monica Busse, Philip Pallmann, Muhammad Riaz, Claire Potter, Fiona J Leggat, Shaun Harris, Andrea Jane Longman, Rachel Lowe, Adrian Edwards, Aloysius Niroshan Siriwardena, Nick Sevdalis, Jackie McRae, Jessica Fish, Bernadette Sewell, Fiona Jones

**Affiliations:** 1 Centre for Trials Research, Cardiff University School of Medicine, Cardiff, UK; 2 Population Health Research Institute, St George's University of London, London, UK; 3 Swansea Trials Unit, Swansea University, Swansea, UK; 4 Primary Care and Public Health, Cardiff University School of Medicine, Cardiff, UK; 5 Community and Health Research Unit, University of Lincoln, Lincoln, UK; 6 Centre for Behavioural and Implementation Science Interventions, National University of Singapore Yong Loo Lin School of Medicine, Singapore; 7 Kingston University, London, UK; 8 School of Health and Wellbeing, University of Glasgow, Glasgow, UK; 9 Department of Clinical Neuropsychology and Clinical Health Psychology, St George's University Hospitals NHS Foundation Trust, London, UK; 10 Swansea Centre for Health Economics, Swansea University, Swansea, UK; 11 Bridges Self Management, London, UK

**Keywords:** Clinical trial, COVID-19

## Abstract

**Objective:**

To evaluate the effectiveness of Listen, a self-management support intervention, for people living with long covid who were not in hospital.

**Design:**

Pragmatic, multicentre, parallel group, randomised controlled trial.

**Setting:**

Twenty four sites in England and Wales.

**Participants:**

Identified from long covid clinic waiting lists, word of mouth, and adverts/social media self-referred to the trial, 554 adults with long covid were randomised to receive either the Listen trial intervention or NHS usual care.

**Interventions:**

The Listen intervention involved up to six one-to-one personalised sessions with trained healthcare practitioners and an accompanying handbook co-designed by people with lived experience and health professionals. Usual NHS care was variable, ranging from no access, access to mobile applications and resources, and to specialist long covid clinics.

**Main outcome measures:**

The primary outcome was the Oxford participation and activities questionnaire (Ox-PAQ) routine activities scale score at three months assessed in the intention-to-treat population. Secondary outcomes included Ox-PAQ emotional wellbeing and social engagement scale scores, the Short Form-12 (SF-12) health survey, the fatigue impact scale, and the generalised self-efficacy scale at three months. The EuroQol five-dimension five-level (EQ-5D-5L) assessed health utility. Serious adverse events were recorded.

**Results:**

Between 27 May 2022 and 15 September 2023, 554 people with long covid (mean age 50 (standard deviation 12.3) years; 394 (72.4%) women) were randomly assigned. At three months, participants assigned to the intervention group reported small non-significant improvements in the primary outcome of capacity for daily activities as assessed by Ox-PAQ routine activities scale score (adjusted mean difference −2.68 (95% confidence interval (CI) −5.38 to 0.02), P=0.052) compared with usual NHS care. For the secondary outcomes, people receiving the intervention also reported significant improvements in mental health (Ox-PAQ emotional wellbeing −5.29 (95% CI −8.37 to −2.20), P=0.001; SF-12 2.36 (95% CI 0.77 to 3.96), P=0.004), reductions in fatigue (fatigue impact score −7.93 (95% CI −11.97 to −3.88), P<0.001), and increases in self-efficacy (generalised self-efficacy scale 2.63 (95% CI 1.50 to 3.75), P<0.001). No differences were found in social engagement (−2.07 (95% CI −5.36 to 1.22), P=0.218) or SF-12 physical health (0.32 (95% CI −0.93 to 1.57), P=0.612). No intervention related serious adverse events were reported.

**Conclusions:**

The personalised self-management support intervention of the Listen trial resulted in non-significant short term improvements in routine activities when compared with usual care. Improvements in emotional wellbeing, fatigue, quality of life, and self-efficacy for people living with long covid were also reported. Physical health and social engagement were not affected by the trial intervention. The limited understanding of how much change is clinically meaningful in this population along with the unblinded design, the use of self-referral as a recruitment method and variable usual care may have introduced unintended bias and thus limits robust conclusions about this intervention. Further research is required to fully establish the impact of the intervention.

**Trial registration number:**

ISRCTN36407216, ISRCTN registry, registered 27 January 2022.

**WHAT IS ALREADY KNOWN ON THIS TOPIC**

- The physical symptoms of long covid, such as extreme fatigue, breathlessness, cognitive difficulties, and joint pains can be fluctuating and episodic and have a major impact on everyday activities, emotional wellbeing, and social participation
- Rehabilitation for people with long covid is variable with National Institute for Health and Care Excellence policy guidance advocating supported self-management depending on need
- Supported self-management interventions that rely on signposting and generic advice and information, rather than personalised treatment approaches, have limited evidence of effectiveness

**WHAT THIS STUDY ADDS**

- Listen is a relatively brief, personalised self-management support intervention that integrates theoretical evidenced based outputs and real-world lived experiences and contexts of people with long covid and not in hospital
- A non-significant effect on our primary outcome of everyday activities was noted
- Secondary outcome data suggested a modest benefit in fatigue management, emotional wellbeing, and confidence to self-manage but no impact on physical health or social engagement

**HOW THIS STUDY MIGHT AFFECT RESEARCH, PRACTICE, OR POLICY**

- Personalised support sessions using core principles, such as those evaluated in the Listen intervention, show promise
- Training healthcare practitioners to listen, validate symptoms, and facilitate problem solving to support other self-management strategies are worth considering when planning health services that accommodate varied needs of people living with long covid
- Further research to explore clinical outcomes of the intervention with a longer follow-up, and implementation across different healthcare settings are warranted

## Background

Long covid, the name agreed by a community of people living with long lasting symptoms following a covid-19 illness, now encompasses the UK's definitions of ongoing symptomatic covid-19 (symptoms from four weeks up to 12 weeks), post covid-19 syndrome (symptoms beyond 12 weeks), and the World Health Organization's post covid-19 condition (symptoms beyond three months and persisting for at least two months).^1^,^3^ In the UK, at least 1.9 million people are estimated to meet the criteria for long covid. Of these, 1.3 million have symptoms lasting for more than a year and 762 000 people live with symptoms for more than two years.^3^ A comprehensive study in 2021 reported a total of 203 symptoms across 10 organ systems,^4^ which are both diverse and fluctuating, and may include fatigue, joint or muscle pain, altered smell or taste, cognitive impairment, anxiety, and sleep disorders. Fatigue is by far the most common symptom (72%), followed by difficulties with cognitive function (51%), muscle aches (49%), and shortness of breath (48%).^1^ The potential legacy of long covid is serious, with a high incidence of individuals not returning to work by six months and continuing to experience limitations in their day-to-day activities,^4^,^6^ which could result in macroeconomic costs of £1.5 billion each year ($1.9 billion; €1.81 billion).^7^

The uncertainty and confusion surrounding long covid, with its varied, relapsing, and remitting symptoms, has also been heightened by a heavy sense of loss and stigma experienced by those living with the condition.^8^ Furthermore, no clear diagnosis of long covid increases the risk of individuals with the condition feeling overlooked and misunderstood by healthcare practitioners and services.^9^ Published reports of individuals being dismissed as anxious while presenting with wide ranging and serious symptoms are concerning, and an ongoing need is to broaden the medical community's knowledge and understanding of the long term consequences of covid-19 and access to timely and adequate care.^1^

The long covid personalised self-management support co-design and evaluation (Listen) trial was a randomised pragmatic effectiveness and cost-effectiveness trial of the co-designed Listen intervention,^10^ one of 15 research projects funded by the UK's National Institute for Health and Care Research (NIHR) long covid research programme in July 2021. The Listen trial intervention integrated evidence that relatively short self-management interventions (eg, up to six sessions over 10-12 weeks) can facilitate positive outcomes, contrary to the principles underpinning many rehabilitation interventions that advocate more is better.^11 12^ The trial also addressed the emerging view that interventions that rely on signposting and information giving, which can be the case for long term conditions including long covid (eg,^13^), have limited evidence of effect.^14 15^ Awareness was growing of the ineffective or possibly harmful^16^ effects of approaches such as a graded exercise programme and a focus on recommendations to personalise treatment approaches through recognition and validation of patient experiences was needed.^17^

The complexity of long covid, with the uniqueness and variability of symptoms impacting on everyday life, presents challenges for interventions or services that do not provide scope or space for personalised support. A personalised intervention that integrates theoretical evidenced based outputs as well as the real world lived experiences and context of people with long covid, is likely to be more impactful.^18^ The Listen intervention was co-designed to ensure that it was contextualised and relevant to the specific challenges and complexity of living with long covid.^19^ The Listen intervention was also informed by national surveys, which showed exclusion of seldom heard groups and individuals who had neither received a positive Covid test nor presented to NHS services.^20^ The extent to which current understanding of long covid, and specifically its impacts and strategies to manage the condition, have included the experiences of people of diverse abilities and people of black, Asian, and minority ethnic backgrounds, were unclear. These groups have also been among the most impacted by covid-19 and therefore strategies to engage and involve them in developing interventions were critical to address marginalisation.^20^

In a randomised controlled trial, we evaluated the effectiveness of the co-designed, personalised, self-management support intervention for people not admitted to hospital for covid-19 but living with long covid. Recognising the highly diverse and fluctuating symptoms affecting multiple aspects of everyday life, we selected participation in routine activities as our primary measure of interest. We also gathered information about social participation, emotional wellbeing, quality of life, fatigue, and self-efficacy, and compared these outcomes in people who received the Listen intervention in the trial and in people who received usual NHS care. Healthcare professionals delivering the Listen intervention were provided with targeted training to ensure they had the knowledge and skills to deliver a personalised approach to supporting self-management.^21^,^23^ They were also provided with ongoing support to enable sustained use of core co-designed intervention principles, including specific language and techniques to support key self-management skills such as problem solving, reflection, and personalised goal setting.^22^ These were also integrated into a fidelity checklist to ensure the intervention was delivered as intended.

## Methods

### Trial design and setting

The Listen trial was a pragmatic, multicentre, two arm, parallel group, individually randomised, controlled trial with both primary and secondary care sites (online supplemental table S1) recruiting in England and Wales. The trial methods were developed, and protocol written, in line with the SPIRIT reporting guidelines^24 25^ and are published elsewhere.^10^ The trial was ended once it had recruited fully, and the last recruited participant had completed their final follow-up assessment. We also conducted an integrated health economic evaluation that assessed the cost-effectiveness of the Listen intervention from an NHS and personal social services perspective and a societal perspective. The health economic analyses will be reported elsewhere.

### Participants

Potential participants completed the expression of interest form, and only if eligible, progressed to consenting and baseline data collection. Eligible participants were aged 18 years or older, had experienced at least one long covid symptom for 12 weeks or longer, and participants had to have consulted with their general practitioner (GP) to rule out complications or the need for further investigation in relation to persistent symptoms following covid-19 infection. Individuals with a palliative condition, or receiving end-of-life care, or people in hospital during the acute phase of covid-19 or actively participating in another long covid intervention trial were not eligible. Participants only progressed past baseline once trial teams were able to allocate them to a participating research site. Potential participants were sent study information by post, email, or text messages, or a combination. Self-referrals were also enabled through broad reaching publicity.

### Public and patient involvement

The NIHR's six standards for public and patient involvement and engagement (UK Standards for Public Involvement) underpinned decision making with regards to all research processes in the Listen trial, including participant facing communications, recruitment strategies, interpretation of study results, and knowledge dissemination. See online supplemental table S2 for detailed description of activities as per Guidance for Reporting Involvement of Patients and the Public version 2 (GRIPP2).^26^

### Recruitment, expression of interest, consent, and demographic data collection

Our primary method of recruitment was self-referral. People who were on waiting lists for the long covid clinics were sent invitation letters in the post. The trial was further promoted via social media and advertisement (posters) within primary and secondary care sites. We also opened 30 primary care (GP practices) participant identification centres in England and Wales. Participants identified via a medical record search at a participant identification centre were sent text messages if on screening of records they had a positive covid test at a GP consultation and had ongoing postcovid symptoms that meant that they may have been eligible for the Listen trial. The text message introduced the Listen trial and included a link to the Listen website for more information.

All potential participants identified from waiting lists for long covid services at Listen trial sites or responding to public advertisements or text messages were required to complete an expression of interest form on the trial website and were then assessed for eligibility. We asked people (n=1026) who completed the online expression of interest forms (who were thus assessed for eligibility) to indicate how they heard about the trial. Responses were as follows: advertisement in press (n=32); recruited by trial site either via letter or referral from healthcare practitioner (n=287); social media (353); and other (n=354)). The most common reasons given for other were: (i) told about the study by a friend or relative; (ii) long covid groups; and (iii) staff emails, newsletters at hospitals, or health boards.

If eligible, participants then went on to complete the electronic informed consent form. If those interested in participating were unable or unwilling to use the internet, they could phone the central research team for assistance in form completion. Data were collected for age, gender, sex at birth, ethnic group, household information, highest educational qualification, employment, use of any community based health and social care services or mental health services in the past three months, and positive covid test. We gathered data for the number of long covid symptoms lasting for 12 weeks or longer across 10 organ systems. These systems were defined by Davis et al^27^ who surveyed 3762 participants with confirmed (diagnostic or antibody positive; n=1020) or suspected (diagnostic or antibody negative or untested; n=2742) covid-19, from 56 countries, with illness lasting over 28 days and onset prior to June 2020. These included cardiovascular symptoms, dermatological symptoms, gastrointestinal symptoms, symptoms of the head, ears, eyes, nose, or throat, immunological symptoms, musculoskeletal symptoms, pulmonary symptoms, reproductive symptoms, systemic symptoms (eg, fatigue, fever, sweats, and coldness), mood and emotion symptoms, cognitive dysfunction, headaches, memory issues, sensory issues, sleep issues, language and speech issues, and smell or taste issues.

### Randomisation and blinding

Participants were individually allocated to either the Listen intervention or to usual care study arm (control group) in a 1:1 ratio using simple randomisation stratified by site. Allocation was implemented via the Listen secure remote web database. The randomisation sequences were generated by the trial statistician in Stata 17 using permuted blocks of randomly varying sizes between two and 10. Participants were randomised by the central trial team following the completion of baseline assessments. Email notifications were sent to the participant and their allocated site advising them of their group allocation. Participants and practitioners were not masked to group allocation. Statistical and health economic analyses were conducted blind to group allocation.

### The Listen intervention

The Listen personalised self-management support intervention^19^ was co-designed with 28 people living with long covid and nine healthcare practitioners working in long covid services. The protocol for the co-design^28^ and intervention^19^ are reported and elsewhere in accordance with the Template for Intervention Description and Replication (TIDieR).^29^ Underpinned by social cognitive theory and self-efficacy principles^30^ to help individuals's build belief in their capability, the Listen intervention was adapted from theory and evidence from Bridges self-management to enhance the knowledge, skills, and confidence of people to manage everyday life with symptoms of long covid.^31^ In the intervention, key sources of self-efficacy included goal mastery and vicarious peer modelling experiences, with self-efficacy as a proposed mediator of change. In this approach to self-management, interactions by healthcare practitioners become less directive and more collaborative, facilitating individuals' problem solving skills. The Listen programme theory highlighted self-efficacy, gaining control and stability of symptoms and knowledge about living day-to-day with long covid as key mechanisms of impact. We hypothesised that this would result in greater capacity to engage in regular activities that form the basis of daily life, better symptom management, and improved emotional wellbeing.

The Listen intervention was underpinned by eight core principles developed and refined through co-design stages. Participants accessed up to six one-to-one personalised self-management sessions with a healthcare professional trained to Listen protocol. The content of sessions were tailored to individuals' ongoing needs and priorities and delivered in accordance with the intervention core principles. These principles included attentive listening, and supporting reflection on everyday strategies, problem solving, and feelings of success. Participants also received the Listen handbook available as a hard copy or interactive PDF. The handbook consisted of five sections including narratives of people living with long covid, symptoms, challenges and solutions to managing the condition, navigating social encounters, space for reflection, and further resources. The six sessions were remotely delivered through video conferencing software (eg, Microsoft Teams or Zoom) or by telephone, and could each last up to one hour. While six sessions were offered, minimum session adherence was three; this number was chosen as a result of discussions during co-design stages and public and patient involvement and engagement meetings and considered the minimum required to establish a collaborative relationship and address core intervention principles. Participants were given the choice of number and frequency of sessions, time of the day, and mode of delivery (online or telephone) based on their needs. Participants were initially required to complete all sessions within a 10 week period. However, sessions could take up to 12 weeks to complete due to factors relating to severity of symptoms, competing pressures such as work, and availability of participant and healthcare professional. Therefore, intervention delivery time was extended to 12 weeks early into the trial to accommodate greater flexibility for sessions. Before delivering the Listen intervention sessions to participants, 72 healthcare practitioners comprising nurses, physiotherapists, occupational therapists, and assistant psychologists completed eight hours of training in the Listen core intervention principles and use of the Listen handbook. To supplement the initial eight hours of training, and to maintain intervention delivery fidelity, healthcare practitioners were given access to an additional support and resource package. This wrap-around support package provided resources and interactive events hosted through a Microsoft Teams channel available only to trained practitioners. The virtual platform contained supporting video files, audio files, and documents, including podcasts, frequently asked questions, and quick guides. Recordings from the 35 approximately bi-monthly, virtual top-up question and answer events were also stored on this platform as additional resources.

### Usual care

Participants randomly assigned to the usual care group (control arm) of the study received NHS routine care as available to them in their region. The availability of long covid care varied geographically, and services differed in size, modality of delivery, and clinical specialty (eg, respiratory or neurology). NHS services for long covid evolved during the trial with the introduction of a tiered system. Care in each tier varied ranging from self-management resources (tier 1), GP support (tier 2), referral to long covid services (eg, respiratory, ear, nose, and throat) (tier 3), or referral to highly specialised services (eg, cardiovascular complications, severe autoimmune dysfunction; tier 4).^32^,^34^ Trial recruitment ceased prior to the integration of long covid care into NHS Integrated Care Boards.^35^ Participation in the Listen study did not guarantee or fast track access to NHS services. However, where possible, the Listen team signposted participants to local NHS services if they requested information. Access to and perceptions of NHS usual care were explored as part of an embedded process evaluation (reported elsewhere).

### Outcomes

Outcomes were measured at baseline, six weeks (for purposes of health economic evaluation only), and three months (all outcomes) after randomisation. The primary outcome measure was the routine activities scale score of the Oxford Participation and Activities Questionnaire (Ox-PAQ) at three months,^36^ a fully validated, patient reported outcome measure developed specifically to assess participation and activity in individuals with chronic health problems. Secondary outcomes were emotional wellbeing (Ox-PAQ emotional wellbeing scale score), social engagement (Ox-PAQ social engagement scale score), health related quality of life (Short Form-12) Health Survey,^37^ fatigue (fatigue impact scale^38^), and perceived self-efficacy to predict coping with daily struggles and adaptation after experiencing stressful life events (generalised self-efficacy scale^39^) with additional covid-19 context specific questions (see online supplemental material), which enabled exploration of the key anticipated mediators of intervention outcome. Information about health utility was captured using the EuroQol five-dimension five-level (EQ-5D-5L) questionnaire that includes five dimensions of health: mobility, self-care, usual activities, pain or discomfort, and anxiety or depression.^40^ Adverse events related to psychological distress and any events meeting the definition of a serious adverse event were recorded. Three brief validated scales, namely the acceptability of intervention measure intervention appropriateness measure and feasibility of intervention measure were used to assess the fitting, suitability, likability, and match of the intervention.^41^ Detail for each of the Listen trial outcome measures including number of items, range, minimum important difference and direction of effect are provided in online supplemental tables S3 and S4.

Adherence to the Listen intervention was defined as attendance at three or more of the one-to-one personalised self-management sessions. Fidelity of intervention delivery was assessed in a purposively selected subsample of recorded sessions against a predefined fidelity checklist that reflected the eight core intervention principles. Results of the intervention fidelity, alongside findings from focus groups of participant interviews and healthcare professionals undertaken as part of a detailed embedded mixed methods process evaluation will be reported elsewhere.

### Sample size

We aimed to detect a minimum clinically important standardised effect size of 0.32^42^ between randomised arms in the primary outcome of the routine activities scale score domain of the Ox-PAQ with 90% power while controlling the two sided type I error level at 5%. This relates to a within group minimum important difference of 7.51.^42^ A conventional individually randomised trial would have required 414 participants (based on a two sample t-test), but because the intervention was expected to be delivered by up to 24 community rehabilitation teams, we also took potential clustering in the intervention arm into account.^43 44^ Assuming an intraclass correlation coefficient of 0.03 in the intervention arm, 24 clusters with 10 participants each in the intervention arm and 234 participants in the usual care arm (ie, a total of 474 participants) was required for 90% power. We calculated these values using Moerbeek and Wong's method,^45^ as implemented in version 0.7.0 of the R package clusterPower.^46^ Assuming 15% loss to follow-up, the overall recruitment target was set to 558 participants.

### Data collection

Study data were collected and managed using REDCap electronic data capture tools hosted at Yale University (CT, USA).^47 48^ Outcomes were self-reported and mostly completed online. All site staff had password protected accounts for REDCap to access participant records and complete intervention session notes, and withdrawal and safety forms.

### Enablers to trial participation and inclusivity

Inclusivity considerations were integral to the trial process. Narratives and experiences from a wide variety of people from different backgrounds, contexts, ages, and genders were captured through online co-design meetings and interviews and were included in the Listen handbook to enhance relatability to different groups. Data were collected on gender, age, sex at birth, and ethnic group of trial participants. To enable a diverse population to participate in the trial, and with support of the Listen patient and public involvement and engagement group, multiple steps were implemented. The Listen intervention handbook was posted to all participants in hard copy and the option to receive the intervention by phone was also provided. One-to-one personalised support sessions were offered to participants in both English and Welsh languages, allowing access for Welsh speaking participants. Next, the trial was set up for individuals to self-refer into, thus improving accessibility. The remote delivery of intervention sessions, via a secure web video conferencing system or telephone, and the flexibility of scheduling sessions, were designed to further maximise access of the intervention for all. For participants with debilitating symptoms or physical impairments, remote delivery and inclusivity methods (eg, breaks, shorter sessions, and cameras off) were used to enhance the feasibility and accessibility of the intervention. All our participant materials were developed with guidance from Diversity and Ability, a social enterprise, led by and for disabled people who support groups to create inclusive cultures within their activities. Additionally, for baseline and follow-up questionnaire completion, and the reading of key study documents, additional support and measures were available. For instance, members of the research team and site staff supported participants by taking verbal informed consent and gave support completing forms over the telephone. On the recommendation of the Listen patient and public involvement and engagement group, all study materials were audio recorded for any additional accessibility needs.

### Statistical analyses

Participant characteristics and outcome scores at baseline were summarised descriptively by randomised allocation (usual care or Listen intervention). In scoring the Short Form-12 (v1 US version) in our population, responses to items BP2 and SF2 were set to missing due to incorrect response options provided during survey administration. We worked with the license providers (Quality Metrics) to ensure that the survey and the physical and mental component scores were correctly scored and interpreted as per the validated outcome measure. The primary analysis used the intention-to-treat population. A linear mixed-effect regression model was fitted with routine activities scale score at three months as a dependent variable, the randomisation group and baseline routine activities scale score as independent variables, and a random centre effect to provide the estimated mean difference of the routine activities scale score at three months for the intervention group compared with the usual care group, alongside a 95% confidence interval (CI) and P value (model A). The regression model accounted for clustering due to centres in both groups. The effectiveness of the intervention on the total scores of the secondary outcomes (emotional wellbeing, social engagement, health related quality of life, fatigue, health utility generalised self-efficacy scale) at three months follow-up, adjusted for baseline, were assessed using similar mixed-effect models as for the primary outcome analysis. In a post hoc responder analysis, we created a binary outcome based on whether or not participants met a change of at least 7.51 (minimum important difference) in Ox-PAQ routine activities scale score and used a mixed-effect logistic regression model with randomisation group as independent variable and a random centre effect to compute and odds ratio for the intervention group versus usual care with 95% CI.

Recognising the potential impact of age, gender, ethnic group, employment status, and number of long covid symptoms, we adjusted the primary and secondary outcome analyses for age, gender, ethnic group, employment status, and number of long covid symptoms, in a secondary prespecified analysis (model B). We also explored prespecified subgroup analyses for gender and ethnic group; however, due to the absence of statistical power, we do not report these results here. To assess the impact of missing data, we conducted a sensitivity analysis using multiple imputation using chained equations with the assumption of missingness at random, creating 20 imputed datasets and combining them according to Rubin's rules. Imputation models included trial arm, centre, participant age, gender, qualification level, employment status, participant reporting a positive covid-19 test, and number of long covid symptoms as independent variables. The assumption of clustering in the intervention arm only was evaluated with both homoscedastic and heteroscedastic models. Based on the postestimation of intraclass correlation coefficient and Akaike information criterion for model fit, clustering was considered in both arms and results from the above models reported. The detailed statistical analysis plan was finalised before any analysis was performed using Stata version 17 and is presented in online supplemental materials.

### Patient and public involvement and engagement

Patients were involved in the design, conduct, reporting, or dissemination plans of this research. Refer to the methods section for further details. We have delivered an extensive communication and engagement plan including publication of the trial and co-design protocols and a qualitative publication and intervention development publication co-written with patient and public involvement and engagement colleagues. We have hosted three online knowledge exchange events that were attended by more than 300 people with long covid, NHS healthcare practitioners, and academics. Summaries from the knowledge exchange webinars have been sent to more than 800 registrants. Several more publications are in review or being prepared for submission.

## Results

The Listen trial was open to recruitment between 27 May 2022 and 15 September 2023 with 15 primary and secondary care NHS centres and one non-NHS site within England set up as individual sites and NHS sites in Wales (covering all seven Health Boards and one non-NHS site) set up as a single site. Baseline characteristics of the total sample and by study arms are presented in table 1. Gender (self-described) and sex (assigned at birth) for our total sample at baseline is presented in online supplemental table S5.

**Table 1:** Baseline characteristics of the total sample and by the study groups

Characteristics	Total sample (N=544), no. (%)	Usual care (N=274), no. (%)	Intervention (N=270), no. (%)
Mean age (SD)	50.0 (12.3)	50.0 (12.1)	50.0 (12.5)
Missing	1 (0.2)	1 (0.4)	0 (0.0)
Gender:			
Female	394 (72.4)	199 (72.6)	195 (72.2)
Male	143 (26.3)	72 (26.3)	71 (26.3)
Other*	7 (1.3)	3 (1.1)	4 (1.5)
Missing†	0 (0.0)	0 (0.0)	0 (0.0)
Ethnic group:			
White	505 (92.8)	255 (93.1)	250 (92.6)
Mixed or multiple ethnic groups:	15 (2.8)	8 (2.9)	7 (2.6)
Asian	15 (2.8)	5 (1.8)	10 (3.7)
Black	5 (0.9)	3 (1.1)	2 (0.7)
Other ethnic group	2 (0.4)	1 (0.4)	1 (0.4)
Missing	2 (0.4)	2 (0.7)	0 (0.0)
Living situation:			
Alone	89 (16.4)	53 (19.3)	36 (13.3)
Partner	171 (31.4)	80 (29.2)	91 (33.7)
Children including adopted ones	58 (10.7)	33 (12.0)	25 (9.3)
Partner and children	160 (29.4)	78 (28.5)	82 (30.4)
Other family member	45 (8.3)	20 (7.3)	25 (9.3)
Non-family member	15 (2.8)	9 (3.3)	6 (2.2)
Missing	6 (1.1)	1 (0.4)	5 (1.9)
Dependents:			
None	349 (64.2)	179 (65.3)	170 (63.0)
Children aged ≤16	153 (28.1)	75 (27.4)	78 (28.9)
An adult reliant on you for any support	36 (6.6)	17 (6.2)	19 (7.0)
Missing	6 (1.1)	3 (1.1)	3 (1.1)
Highest level of qualification:			
No qualifications	12 (2.2)	5 (1.8)	7 (2.6)
1-4 GCSEs or equivalent	39 (7.2)	16 (5.8)	23 (8.5)
≥5 GCSEs or equivalent	50 (9.2)	30 (11.0)	20 (7.4)
Apprenticeship	4 (0.7)	2 (0.7)	2 (0.7)
≥2 A levels or equivalent	73 (13.4)	31 (11.3)	42 (15.6)
Degree level or above	343 (63.1)	182 (66.4)	161 (59.6)
Other qualifications	17 (3.1)	7 (2.6)	10 (3.7)
Missing	6 (1.1)	1 (0.4)	5 (1.9)
Employment status:			
In full time education	28 (5.2)	13 (4.7)	15 (5.6)
In part time education	7 (1.3)	6 (2.2)	1 (0.4)
House person	13 (2.4)	8 (2.9)	5 (1.9)
Employed (full time)	230 (42.3)	121 (44.2)	109 (40.4)
Employed (part time)	121 (22.2)	54 (19.7)	67 (24.8)
Unemployed	58 (10.7)	28 (10.2)	30 (11.1)
Retired	82 (15.1)	42 (15.3)	40 (14.8)
Missing	5 (0.9)	2 (0.7)	3 (1.1)
In the past three months, use of any community based health and social care services:			
Yes	111 (20.4)	54 (19.7)	57 (21.1)
No	425 (78.1)	217 (79.2)	208 (77.0)
Missing	8 (1.5)	3 (1.1)	5 (1.9)
In the past three months, use of any community based mental health services:			
Yes	64 (11.8)	34 (12.4)	30 (11.1)
No	470 (86.4)	234 (85.4)	236 (87.4)
Missing	10 (1.8)	6 (2.2)	4 (1.5)
Positive covid-19 test:			
Yes	479 (88.1)	247 (90.1)	232 (85.9)
No	65 (12.0)	27 (9.9)	38 (14.1)
Missing	0 (0.0)	0 (0.0)	0 (0.0)
No. of long covid symptoms:			
Median (interquartile range)	12 (9-14)	12 (9-14)	12 (10-14)
Range	1-18	1-18	1-18
Missing	0 (0.0)	0 (0.0)	0 (0.0)

* Self-identified gender categories were woman, man, transwoman, non-binary/genderqueer/agender/gender fluid, prefer not to say, or other.

† Self-identified gender was missing for 1one participant and replaced with their sex at birth.

**Figure 1:**
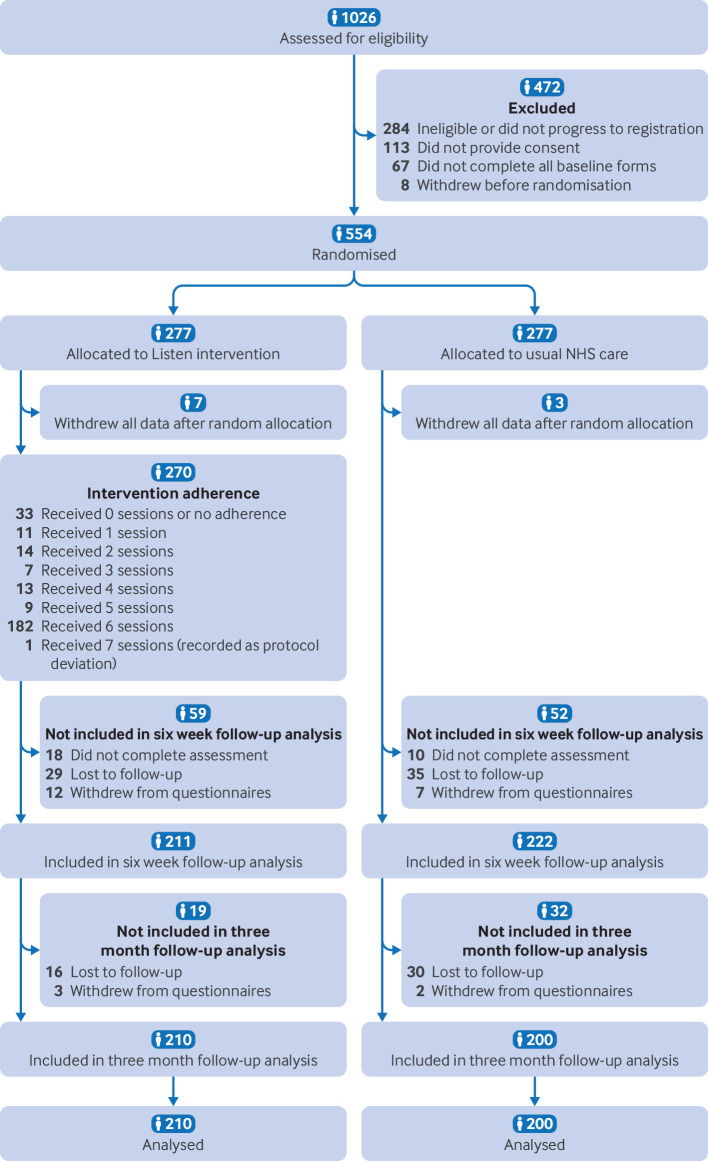
CONSORT diagram showing the flow of participants through the Listen trial. Loss to follow-up and partial withdrawals from questionnaires are reported at six week and three month follow-up. Participants who did not complete the six week follow-up but did complete the three month follow-up (n=18 and n=10 respectively) are also indicated

Our sample broadly matched that of Office for National Statistics data for the condition with a greater prevalence of long covid in women and most likely to affect those aged 35-69 years. Despite statistical uncertainty, Office for National Statistics data suggest long covid is more prevalent in white ethnic groups than in people of black, Asian, or mixed ethnic groups.^6^ The participants in Listen were overwhelmingly from white ethnic groups, despite our use of multiple strategies to recruit from across diverse ethnic communities.^10^ Overall, 42.3% were in full time employment and 22.2% in part-time employment. Most people had been educated to GSCE level and above, and were living with a partner (31.4%), or partner and children (29.4%). 20.4% had accessed community health and social care and 11.8% had accessed or community mental health services in the three months prior to enrolling in the Listen trial. Across both groups, 88.1% had received a positive covid-19 test and had experienced a median of 12 different symptoms related to their long covid. Across the trial, 554 participants were randomly assigned to the trial intervention (n=277) or usual care (n=277). The six week follow-up was completed by 211 participants in the Listen group and 222 in usual care group, and at the three month follow-up, the numbers were 210 in the intervention group and 200 in the usual care group (figure 1; online supplemental tables S6–S10).

Participants who received the Listen intervention perceived it to be acceptable, feasible, and appropriate (table 2). Adherence to the Listen intervention was good, with 78.5% of those allocated to receiving the intervention meeting the criteria for adherence (ie, attending at least three sessions) and only 12% not attending any sessions (table 3; online supplemental table S11). Seven adverse events were reported in the Listen intervention group and three in the usual care group. Of the seven intervention group participants, only one discontinued the intervention and was referred to the local mental health crisis team. Two serious adverse events were reported in the Listen intervention group and one in the usual care group. None of the reported serious adverse events was considered related to the intervention (online supplemental tables S12 and S13).

**Table 2:** Implementation outcomes completed by trial participants

Implementation measure	Median (interquartile range)
Acceptability of Intervention measure score	17 (16-20)
The intervention appropriateness measure score	16 (16-20)
Feasibility of intervention measure score	16 (16-19)

Highest score possible is 20, with higher scores indicating greater acceptability, appropriateness, and feasibility, respectively (table S3 in supplementary material).

**Table 3:** Summary of Listen intervention adherence

No. of sessions	No. (%) of intervention group participants (total n=270)
Full adherence (n=212):	
6	183* (67.8%)
5	9 (3.3%)
4	13 (4.8%)
3	7 (2.6%)
Partial or no adherence (n=58):	
2	14 (5.2%)
1	11 (4.1%)
0	33 (12.2%)

* One participant received a repeated 1stfirst session due to a change in practitioner.

**Figure 2:**
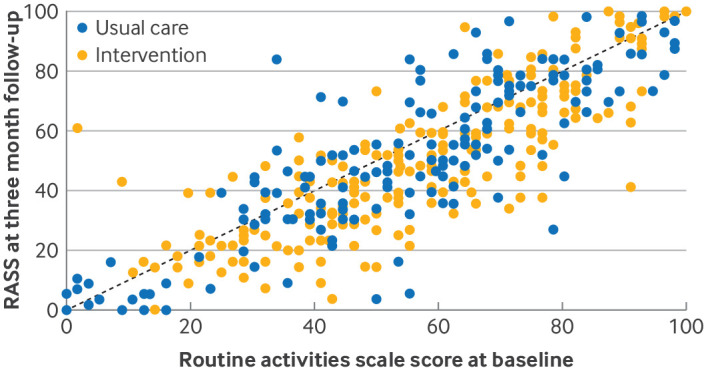
Scatter plot of individual Ox-PAQ routine activities domain scores at baseline (x axis) and three month follow-up (y axis) by treatment group (intervention or control). The dashed diagonal line indicates no change between baseline and follow-up with data points below the dashed line indicating greater ability to participate in routine activities at follow-up compared with baseline (lower scores on routine activities scale score are a better outcome)

Outcomes at the three month follow-up are presented in table 4. The individual datapoints for each participant at baseline (x axis) and follow-up (y axis) for the Ox-PAQ routine activities scale score are shown in figure 2. Our primary analysis indicated that people who had received the Listen intervention had greater capacity for daily activities as assessed by the Ox-PAQ routine activities scale score at three months (adjusted mean difference −2.68 (95% CI −5.38 to 0.02), P=0.052). The adjusted mean difference in a per-protocol analysis, which included participants attending three or more sessions adjusted for the fixed effect of baseline outcome score and random effect of site, was −2.79 (−5.57 to −0.01), P=0.049 for the primary outcome (Ox-PAQ routine activities domain score). Participants also reported improved emotional wellbeing and mental health (Ox-PAQ emotional wellbeing scale score −5.29 (−8.37 to −2.20), P=0.001; Short Form-12 mental health component 2.36 (0.77 to 3.96), P=0.004), reduced fatigue impact (fatigue impact scale −7.93 (−11.97 to −3.88), P<0.001), and increased self-efficacy (generalised self-efficacy scale 2.63 (1.50 to 3.75), P<0.001). No between-group differences in social engagement (social engagement scale score −2.07 (−5.36 to 1.22), P=0.218) or Short Form-12 physical health component (0.32 (−0.93 to 1.57), P=0.612).

**Table 4:** Description of the outcomes and their comparison between the study groups at three months follow-up

Outcomes	Baseline (N=544),mean (SD)		Follow-up (N=410),mean (SD)		Adjusted effect estimate β (95% CI)	P value
	**Usual Care (n=274)**	**ListenIntervention (n=270)**	**Usual Care(n=200)**	**ListenIntervention(n=210)**		
**Primary outcome**						
Ox-PAQ routine activities domain score (range 0-100)	55.8 (23.0)	56.7 (22.5)	51.8 (26.4)	49.8 (24.3)	−2.68 (−5.38 to 0.02)*;−2.90 (−5.66 to −0.15)†	0.052;0.039
Missing no. (%)	4 (1.5)	2 (0.7)	1 (0.5)	2 (1.0)	—	—
**Secondary outcomes**						
Ox-PAQ emotional wellbeing domain score (range 0-100)	58.5 (21.3)	60.6 (21.6)	54.7 (25.7)	50.6 (22.4)	−5.29 (−8.37, −2.20)*;−5.89 (−8.99 to −2.79)†	0.001;<0.001
Missing no. (%)	0 (0.0)	0 (0.0)	0 (0.0)	0 (0.0)		
Ox-PAQ social engagement domain score (range 0-100)	50.1 (24.8)	51.5 (26.2)	48.7 (26.5)	47.0 (26.0)	−2.07 (−5.36 to 1.22)*;−2.81 (−6.19 to 0.57)†	0.218;0.103
Missing no. (%)	0 (0.0)	0 (0.0)	0 (0.0)	0 (0.0)	—	—
Fatigue impact scale scores:						
Cognitive dimension (range 0-40)	22.8 (9.8)	23.2 (9.7)	21.6 (10.7)	19.9 (10.4)	−2.11 (−3.29 to −0.92)*;−2.34 (−3.56 to −1.12)†	0.001<0.001
Physical dimension (range 0-40)	25.4 (9.0)	26.3 (8.6)	23.1 (10.1)	22.7 (9.5)	−1.53 (−2.63 to −0.42)*;−1.80 (−2.93 to −0.67)†	0.007;0.002
Social dimension (range 0-80)	41.2 (18.7)	43.2 (19.1)	39.2 (20.9)	36.5 (19.8)	−4.22 (−6.35 to −2.09)*;−4.63 (−6.81 to −2.45)†	<0.001;<0.001
Overall score (range 0-160)	89.3 (35.1)	92.7 (35.3)	83.9 (39.6)	79.1 (37.9)	−7.93 (−11.97 to −3.88)*;−8.65 (−12.79 to −4.52)†	<0.001;<0.001
Missing no. (%)	0 (0.0)	0 (0.0)	0 (0.0)	0 (0.0)	—	—
EQ-5D-5L scores:						
Index score (range: 0-1)	0.52 (0.25)	0.49 (0.28)	0.53 (0.30)	0.53 (0.28)	0.03 (−0.01 to 0.06)*0.04 (0.00 to 0.07)†	0.137;0.046
Missing no. (%)	5 (1.8)	6 (2.2)	6 (3.0)	5 (2.4)	—	—
VAS score (range: 0-100):	45.7 (20.9)	44.9 (20.8)	48.4 (23.7)	49.7 (21.6)	1.08 (−2.41 to 4.56)*;2.72 (−0.80 to 6.24)†	0.545;0.130
Missing no. (%)	8 (2.9)	5 (1.8)	10 (5.0)	4 (1.9)	—	—
Generalised self-efficacy scale scores:						
Original 10 items scale (range 10-40)	26.9 (5.8)	26.3 (5.9)	26.5 (6.5)	27.5 (6.3)	1.25 (0.39 to 2.11)*;1.42 (0.54 to 2.30)†	0.005;0.002
Covid four items scale (range 4-16)	9.7 (2.5)	9.3 (2.5)	9.7 (2.7)	10.8 (2.7)	1.31 (0.87 to 1.75)*;1.38 (0.93 to 1.82)†	<0.001;<0.001
Original scale with covid four items, overall score (range: 14-56)	36.6 (7.5)	35.6 (7.6)	36.2 (8.5)	38.4 (8.2)	2.63 (1.50 to 3.75)*;2.79 (1.66 to 3.93)†	<0.001;<0.001
Missing no. (%)	0 (0.0)	0 (0.0)	1 (0.5)	0 (0.0)	—	—
Short Form-12 scores:						
Physical health (range: 0-100)	32.3 (9.6)	31.9 (8.9)	33.1 (10.6)	32.8 (9.7)	0.32 (−0.93 to 1.57)*;0.48 (−0.74 to 1.71)†	0.612;0.440
Mental health (range: 0-100)	37.7 (10.8)	36.1 (10.2)	39.0 (11.2)	40.0 (10.6)	2.36 (0.77 to 3.96)*;2.85 (1.23 to 4.46)†	0.004;0.001
Missing no. (%)	0 (0.0)	1 (0.4)	0 (0.0)	0 (0.0)	—	—

β: Regression coefficients (estimated difference of mean outcome scores at three months follow-up between the study groups) adjusted for baseline outcome scores ≈ (difference of mean outcome scores change in the study groups from baseline to three months follow-up).

* In model A, eEffect estimates, 95% CIs, and p-P values from linear mixed--effects models with the outcome scores at three months follow-up as dependent variable and study arm as an independent variable, were adjusted for the random effect of site and fixed effect of baseline outcome scores.

† IModel B included n the models described in model A with, effect estimates are further adjusted for the fixed effects of age, gender, ethnic groupity, employment status, and the number of Long Covidlong covid symptoms at baseline. These additional covariate adjustments were pre-specified in the statistical analysis plan.

CIconfidence intervalEQ-5D-5LEuroQol Group health related quality of life questionnaireOx-PAQOxford Participation and Activities QuestionnaireSDstandard deviationVASVisual Analogue Scale

When adjusting further for the outcome score at baseline, age, gender, ethnic group, employment status, and the number of long covid symptoms at baseline, a statistically significant improvement in the primary outcome (Ox-PAQ routine activities scale score) (−2.90 (−5.66 to −0.15); P=0.039) was noted when compared with scores of people allocated to usual care at the three month follow-up. Similarly, scores were significantly lower in the emotional wellbeing scale score domain of the Ox-PAQ, reflecting current emotional wellbeing (−5.89 (−8.99 to −2.79); P<0.001), all three domains of the fatigue impact scale and the overall fatigue impact scale (−8.65 (−12.79 to −4.52); P<0.001), the generalised self-efficacy scale with (2.79 (1.66 to 3.93); P<0.001) and without (1.42 (0.54 to 2.30); P=0.002) additional covid-related items, and the mental health component of the Short Form-12 (2.85 (1.23 to 4.46); P=0.001). No between-group differences remained in social engagement (social engagement scale score −2.81 (−6.19 to 0.57); P=0.103) which focuses on maintenance of personal and community relationships, or in the physical health component of the Short Form-12 (0.48 (−0.74 to 1.71); P=0.440). Of the participants who provided primary outcome data (n=402), 161 (40.1%) reported changes of at least 7.51 (minimum important difference) on the Ox-PAQ routine activities scale score and the odds of achieving this were higher in people who received the Listen trial intervention (unadjusted odds ratio 1.40 (0.94 to 2.09); fully adjusted odds ratio 1.46 (0.95 to 2.25)).

Sensitivity analyses using multiple imputation for missing observations resulted in very similar effect estimates, 95% CIs, and P values indicating little effect of missing data for the results reported here (online supplemental tables S14 and S15).

## Discussion

We report on the short-term impact of the Listen intervention, the first personalised self-management support intervention for people with long covid (and not in hospital for initial covid-19 infection) to be tested at scale across England and Wales. The Listen intervention was co-designed to focus on gaining control and stability of symptoms at a time when people were describing real helplessness and hopelessness. We hypothesised that those who received the Listen intervention would feel more in control of their symptoms, which would then impact on their everyday life and emotional wellbeing.^10^ Ratings of self-efficacy were explored as part of our assessment of intervention fidelity. Listen participants reported improvements in self-efficacy, increased feelings of control of symptoms such as fatigue and pain, and subsequent improvements in emotional wellbeing and quality of life. These findings must, however, be balanced with the limited understanding of how much change is clinically relevant in this study population and the preliminary single arm study in a small sample of people living with long term health conditions that informed our understanding of the Listen primary outcome.^42^ We observed an average reduction of less than three points in the Ox-PAQ routine activities scale score for people receiving the Listen intervention compared with usual care, less than half the published minimum important difference for Ox-PAQ routine activities scale score of 7.51 points. Also, the 95% CI of the difference between groups includes a null effect (for the primary analysis) or very small and clinically insignificant improvements close to a null effect (for the additional adjusted analysis). Thus, while our data are supportive of our proposed mechanism of impact, we are not able to make firm conclusions about the Listen intervention leading to clinically meaningful benefit.

Trial participants rated the Listen intervention as acceptable, feasible, and appropriate and 78% of those allocated to the intervention met the minimum criteria for adherence, an indicator of the success of this approach. A per-protocol analysis, which included only participants who attended at least 50% (ie, three or more) of their Listen intervention sessions, did not alter the interpretation of our data. Importantly, the literature indicates that the quality of the interactions and support and level of personalisation received during the intervention sessions are more important in ensuring successful self-management intervention outcomes than the intensity (or dose/number) of sessions received.^12^ Our participants were able to receive up to six sessions with a trained practitioner, with a view to enabling the collaborative relationship and encouraging opportunities for self-management activities and reflection on progress. In practice, however, some participants did not need as many as six sessions for the intervention to be delivered as intended (ie, with fidelity) and for participants to achieve the outcomes that they wanted.

The National Institute for Health and Care Excellence (NICE) guidelines for self-management and supported self-management for people with long covid (last updated January 2024) still focus primarily on advice, information, and setting realistic goals.^2^ Given the multiple components that might be included in long covid interventions, it is unsurprising that the rehabilitation intervention protocols (with or without inclusion of self-management support) registered to date typically involve cohort, case-controlled, before-and-after and non-randomised experimental studies. Additionally, they include complex combinations of aerobic or strength exercises, tele-rehabilitation, cognitive rehabilitation, virtual reality rehabilitation, breathing exercises, cognitive behavioural therapy, mindfulness, and various long covid-specific approaches including activity management and nutrition.^49^ However, the recently published REGAIN trial provides the first evidence from a large randomised trial that an online, home based, group, multi-component physical and mental health rehabilitation intervention is effective in improving health-related quality of life in adults with long covid who were initially admitted to hospital.^50^ ^51^ The authors note that the relative contributions of the intervention components are unknown, but that rehabilitation efforts should target fatigue, pain interference, and depression.

When comparing the findings from REGAIN to the Listen trial, the key difference is that the REGAIN population were in hospital whereas those enrolled in Listen were not admitted to hospital following the covid-19 infection; this may explain the differences in these two trials in terms of participant demographics (REGAIN enrolled more men, fewer white and on average older participants who were hospitalised for covid infection).^51^ Interestingly, both trial interventions inferred positive outcomes for health-related quality of life as measured by the EQ-5D index, fatigue impact, and emotional wellbeing.

Although access to self-management support can be integral to many rehabilitation programmes and is recommended by NICE, the core intervention principles and theoretical underpinnings are often unspecified, and the evidence for the individual self-management support components of rehabilitation in long covid is limited.^52^ Personalisation of self-management support interventions has been shown to be a key indicator of success on both clinical and holistic outcomes and those that integrate collective and learnt strategies of people as well as a focus on what matters most, are critical when designing interventions ready for evaluation. In an emerging condition, such as long covid, capturing this learning and priorities for self-management support was important. Interviews with 18 people living with long covid (who were members of the Listen co-design groups) indicated that seeking reassurance and knowledge, developing greater self-awareness through monitoring, learning from others about what had worked for them, building in moments of joy and purpose, and prioritising what is most meaningful, were all important in navigating life with long covid.^53^

Self-management (support) is an integral part of treatments available for people living with complex long term conditions and long covid appears similar. However, while other research groups are exploring self-management practices of people with long covid,^54^ and, importantly, which have informed outcome measure development,^55^ few interventions have been developed specifically for this group^19^ or evaluations of such approaches. A search for registered trials and studies investigating isolated self-management support aor education interventions for people with long covid highlighted the Listen trial and, to our knowledge, one other registered randomised controlled trials (https://clinicaltrials.gov/study/NCT05268523)^56^ focusing explicitly on self-management support. While recognising interventions addressing specific symptoms such as cognitive and respiratory difficulties are in development and findings from studies will be emerging, currently no cure for long covid exists. An intervention such as the Listen personalised self-management intervention, which can support some of the 1.9 million people in the UK living with long covid, could enable people to feel more in control of their symptoms and learn skills and knowledge to engage in and manage everyday meaningful activities.

While our data highlights the value of personalised support from trained healthcare practitioners on self-management strategies to carry out everyday activities, we acknowledge the inherent limitations of this unblinded trial. Most of our trial population were female (approximately 72%) and from a white ethnic background (92%). Our analyses controlled for age, gender, ethnic group, employment status, and number of long covid symptoms, but was not sufficiently powered to explore outcomes by gender or ethnic group and as such, our findings may have limited generalisability. We also did not examine the impact of factors such as health literacy or educational attainment. Potential participants self-referred to the trial by completing an expression of interest form on the trial website. While telephone support was available, a level of digital exclusion cannot be discounted. Allowing self-certification of at least one symptom consistent with SARS-CoV-2 infection during the acute phase and close contact of a confirmed case of covid-19 around the time of onset rather than providing evidence of a confirmed positive antigen test, as part of the self-referral process, may also have led to further ascertainment bias. While exploring outcomes according to recruitment source (ie, advertisement in press, letter/referral from healthcare practitioner, social media, and other) would have been interesting, such an analysis would have been underpowered due to the small subgroups. The short timeframe of the Listen trial is an additional limitation, as is the higher-than-anticipated loss to follow-up rate at the primary endpoint. Funder requirements meant that the outcomes reported here are limited to data collected at the three month endpoint and as such, an understanding of longer term impacts and cost-effectiveness is lacking.

In 2021, when the Listen trial was conceived, long covid was a new, emerging condition, with a limited knowledge base to guide intervention approaches and variable or non-existent NHS services. The trial was designed, and funding secured in 2021, at a time when no consensus on core outcomes for long covid research was available. We selected a primary outcome, namely the Ox-PAQ routine activities scale score, which reflected the multiple aspects of participation that could be impacted on by the wide ranging symptoms of long covid. This patient reported outcome measure is psychometrically sound and valid, developed for use in a range of health conditions and valid for self-administration. It is theoretically grounded in the World Health Organization International Classification of Function and intended for use in the meaningful evaluation of interventions aimed at promoting participation and activity. Since that time, domains of importance for long covid outcomes have been published. Encouragingly, the outcome domains, namely fatigue, pain, cognitive, mental, and physical health that were measured in Listen, alongside our focus on participation in daily living, are now recognised as being relevant and important in the field despite no consensus still for any single instrument that assesses impact on daily life.^57^

In a pragmatic unblinded effectiveness trial, such as ours, and indeed, as in most, if not all rehabilitation trials, we cannot exclude the potential for performance bias impacting our results. Participants could not be blinded to which intervention they were allocated to. This may have resulted in an element of behaviour change simply as result of being a participant in a trial. We were also aware that the confounding nature of a pandemic and healthcare provision itself resulted in variable offerings for usual care; our data were collected a time when most long covid clinics were set up in England and accessible in some but not all parts of Wales. We could not standardise what usual care involved or was provided, and we could not control for contact time between groups. To ensure that this did not invalidate the main findings, we accounted for this variability of care in our prespecified statistical analyses with the use of a mixed effect model with a random effect for the study centre. To further inform our understanding of trial outcomes, in relation to the intervention received, we captured consistency and inconsistency of usual care versus the trial intervention as a mechanism of impact in the trial process evaluation. That is, whether usual care included certain key characteristics that are reflected in the underpinning trial intervention logic model for example personalisation, being heard, supporting problem solving versus providing information, diagnostics, and monitoring. This mixed method process evaluation will be reported elsewhere.

The Listen trial found that a relatively brief, personalised self-management support intervention that integrates theoretical evidenced based outputs and real world lived experiences and contexts of people not initially admitted to hospital with long covid resulted in non-significant short-term improvements in routine activities when compared to usual care. Improvements in secondary outcomes of emotional wellbeing, fatigue, quality of life, and self-efficacy were seen with the intervention. We suggest that interventions for long covid that provide personalised self-management support delivered by highly trained NHS staff may be preferable to one-time advice and information sessions. Clear next steps are available to enable learning from this research, including formal exploration of what works for whom and in what circumstance. Examining the generalisability of our findings to different age and ethnic groups and studying the impact over a longer period would also be useful.

.

## supplementary material

10.1136/bmjmed-2024-001068 online supplemental file 1

10.1136/bmjmed-2024-001068 online supplemental file 2

10.1136/bmjmed-2024-001068 online supplemental file 3

10.1136/bmjmed-2024-001068 online supplemental file 4

## Data Availability

Data are available upon reasonable request.
