# Supplementary material for: Effectiveness of a personalised self-management intervention for people living with long covid (Listen trial): pragmatic, multicentre, parallel group, randomised controlled trial
Source: BMJ Med. 2025 Jan 31;4(1):e001068. doi: 10.1136/bmjmed-2024-001068 (PMC11881025; doi:10.1136/bmjmed-2024-001068)
Supplement: online supplemental file 2 [file bmjmed-4-1-s002.pdf]

Effectiveness of a Personalised Self-management Intervention for People Living with Long Covid: the LISTEN randomised controlled trial.

**Supplementary Materials.**	**Page**
Supplementary Methods	**2-8**
Supplementary Tables	**9-16**

Supplementary Methods

Table S1. Participating NHS and non-NHS sites.

LISTEN Sites
Wales (all 7 health boards*)
Coventry and Warwickshire
South Warwickshire
Guys & St Thomas
Sheffield
Hounslow & Richmond
Epsom & St Helier
Wrightington, Wigan and Leigh
Norfolk
Mid and South Essex
Essex Partnership University Trust
Banbury Cross Practice
St Bartholomew's
Eynsham
Central & North West London
West London
Bridges (non-NHS site)

***Aneurin Bevan University Health Board, Betsi Cadwaladr University Health Board, Cardiff and Vale University Health Board, Cwm Taf Morgannwg University Health Board, Hywel Dda University Health Board, Powys Teaching Health Board, Swansea Bay University Health Board**

Table S2. Guidance for Reporting Involvement of Patients and the Public version 2 (GRIPP2).

Section and topic	Patient & Public Involvement and Engagement (PPIE) item to report on	LISTEN Project
1: Aim	Report the aim of PPIE in the study	To assist the research team at all stages of the LISTEN project, including the project conception, the co-design of the LISTEN intervention, the processes in the setup and undertaking of the clinical trial and in the dissemination of the research.<br><br>The team sought to work collaboratively with the patient and public members with mutual respect and decision-making.
2: Methods	Provide a clear description of the methods used for PPIE in the study	During the project conception, many conversations were undertaken between the co-chief investigators and people in their network living with Long Covid. Three of these people living with Long Covid were subsequently recruited to a PPIE group to refine the project further, and one of these members was also invited to join the Trial Management Group (TMG). Those in the initial PPIE group were involved in the refining of the work packages and selecting appropriate outcomes measures for use in the trial and assisting with recruitment to the first work package, the co-design of the intervention.<br><br>To co-design the intervention, twenty-eight people with Long Covid were recruited. Multiple, varying inclusive opportunities were provided for people to get involved in the co-design phase (e.g., one-to-one telephone calls and virtual group meetings). Researchers worked with Diversity and Ability, a social enterprise, and Long Covid Support, a patient-led charity, to recruit people with Long Covid, including those from diverse and seldom heard groups. People in the co-design phase shared their experiences and their priorities within multiple group co-design meetings (up to 4), in communications with the research team and through 3 online surveys. (reference co-design protocol and co-design process paper).<br><br>Following the co-design phase, an additional four people living with Long Covid who participated in the intervention co-design expressed a desire to continue supporting the project. They subsequently joined the PPIE group bringing the total to seven members.<br><br>PPIE group meetings were hosted four times per year, roughly every 3 months. Meetings lasted no longer than 90 minutes at any one time, and measures were put in place to maximise the accessibility of the meetings (e.g., virtual meetings, choice to have cameras off, regular breaks). Options for meeting times and dates were provided to the group, and the most popular choice was mutually decided. Meetings were also organised with plenty of advanced notice (at least 2 weeks) to enable members to plan accordingly to maximise attendance and not flare up Long Covid symptoms by joining in. Meeting summaries were circulated after the PPIE meetings to update members who could not attend, and to provide members with a recap of the discussion to reflect upon and add to if they chose.
3: Study results	Outcomes—Report the results of PPIE in the study, including both positive and negative outcomes	PPIE group members contributed to the study (and wider project) in multiple ways, including:<br>- Enhancing participant recruitment – the group suggested recruitment strategies, supported the creation of recruitment materials (e.g., posters) and events (e.g., radio, videos for the website) which led to increases in participant recruitment figures<br>- Adapting participant-facing materials – the group reviewed all language used in participant-facing materials to ensure appropriateness and generated the concept for the audio participant information sheet (PIS) as reading was considered too cognitively demanding and fatiguing.<br>- Piloting and testing data collection processes – the group piloted outcome measure sets for feasibility, burden and practicality, and completed tests on the LISTEN database<br>- Providing Long Covid context updates – the group shared information about updates in Long Covid social media platforms, NHS care services and upcoming Long Covid events<br>- Co-authoring research publications – the group have supported the data analysis and write up of two peer-reviewed journal publications to maximise the accessibility of the research to the public

		- Disseminating the research – the group supported how the research would be shared with the general public, healthcare professionals and policy makers, and opted for a virtual 3-part webinar series. The group also attended and have spoken at national conferences about their experiences engaging in the PPIE for the LISTEN project.<br><br>Other members from the co-design phase, not in the PPIE group, also contributed to the trial in the following ways:<br>- Enhanced the quality of the intervention training – supported the practitioner intervention delivery by attending the training and sharing experiences which practitioners described as incredibly useful and facilitating for their own understanding of the condition.<br>- Enhanced the quality of the intervention delivery support package – supported the creation of resources (e.g., Q&A sessions, newsletters), and attended live support sessions to guide practitioners in supporting their participants.<br><br>Not only have these PPIE group and co-design members supported intervention development, research processes and outcomes, but these opportunities have also impacted themselves directly.<br>To capture the outcomes of participation in the co-design work package, group reflections were collected by an external company. Together, these highlighted how involvement in LISTEN helped people to feel purposeful, feel valued and respected, and feel validated. When facing a fluctuating, episodic long-term condition, involvement brought joy, a cathartic release, and “like a warm hug” from other’s experiencing similar feelings.<br><br>PPIE group members reported similar positive impacts on themselves. Exposed to new opportunities, PPIE members have described how LISTEN gave them the opportunity for personal growth and provided feelings of purpose and confidence in life; something that had previously been stripped away due to their Long Covid.
4: Discussion and conclusions	Outcomes—Comment on the extent to which PPIE influenced the study overall. Describe positive and negative effects	PPIE was integral to this project; it was very effective and influenced key aspects of the intervention delivery and clinical trial processes (outlined in section 3). This might have been related to several factors. Firstly, the research team who led the PPIE meetings were experienced at involving patients and the public in research. As several of the PPIE members were not previously known to the research team, these skills likely fostered the rapport needed to grow mutual trust and respect between individuals. In addition, many of the PPIE members were involved in the project from the outset, or from the beginning of the intervention co-design, allowing them to shape the project from the start. Having the funding to finance PPIE members time, from the outset, helped the research team to emphasise the value placed in their support. Finally, a couple of pre-existing partnerships between the researchers and PPIE group meant that the group was hosted in an accessible format. Held virtually, with cameras optional, and with breaks included, people could come along and contribute while also managing Long Covid symptoms.<br><br>Despite working with external organisations to recruit people with Long Covid to the co-design group and PPIE group, the groups required greater representation from people from different ethnicities and marginalised backgrounds. Although some members were male, and younger or older ages, and from mixed ethnicities, the majority of members were white, middle-aged, and female.
5: Reflections/critical perspective	Comment critically on the study, reflecting on the things that went well and those that did not, so others can learn from this experience	PPIE was embedded throughout the ISTEN project and integral to decision making. The PPIE group fostered far-reaching positive impacts in multiple ways. While not a formal aim of the project, the PPIE undertaken showcased how people with lived experiences can be involved in research and foster contributions that enhance research processes. The methods and strategies learnt and used to undertake the PPIE in LISTEN will be taken forward into other clinical trials research involving people with other long-term conditions. However, future PPIE will seek to improve upon the diversity of the group.

Table S3. Details of specific outcome measures, number of items, possible range and direction of effects.

Outcome measure	Number of items	Total possible range	Published Minimally Important Difference (MID)	Direction of effect
Oxford Participation and Activities Questionnaire (Ox-PAQ) Routine Activities scale score (RASS)	14 items	Range: 0-100	7.51[1]	Lower scores indicate greater participation
Ox-PAQ Emotional Wellbeing scale score (EWSS)	5 items	Range: 0-100	10.77[1]	Lower scores indicate greater participation
Ox-PAQ Social Engagement scale score (SESS)	4 items	Range: 0-100	5.47[1]	Lower scores indicate greater participation
Short Form-12 (SF-12) Health Survey	12 items	Range: 0-100	Physical health component summary: 2.3[2]<br>Mental health component summary: 1.4[2]	Higher scores indicate greater health related quality of life
Fatigue Impact Scale (FIS)	Cognitive functioning subscale (10 items), physical functioning sub-scale (10 items), and psychosocial functioning sub scale (20 items)	Range: 0-160	9-24[3]	Lower scores indicate less impact of fatigue
Generalised Self-Efficacy Scale (GSES) (see Table S4 for details of additional Covid-19 specific items)	10 items in original scale plus 4 Covid specific items	Range: 14-56	Not available. https://userpage.fu-berlin.de/%7Ehealth/faq_gse.pdf	Higher scores indicate greater self-efficacy
EuroQol five-dimension five-level (EQ-5D-5L)	5-item questionnaire with an additional visual analogue scale (VAS)	EQ-5D Index Score Range: 0-1<br>EQ-5D VAS Range: 0-100	EQ-5D-5L index score: 0.037-0.069[4]	EQ-5D Index Score: 1 indicates full health, 0 indicates as bad as being dead<br>EQ-5D VAS: 0 indicates worst health, 100 indicates best health
Acceptability of Intervention Measure (AIM)	4 items	Range: 4-20	Not applicable	Higher scores indicate stronger perceptions of acceptability
Intervention Appropriateness Measure (IAM)	4 items	Range: 4-20	Not applicable	Higher scores indicate stronger perceptions of appropriateness
Feasibility of Intervention Measure (FIM)	4 items	Range: 4-20	Not applicable	Higher scores indicate stronger perceptions of feasibility

Table S4. The Generalised Self-Efficacy Scale including additional four Long Covid specific items*.

Variable Name	Variable Label	Value	Value Label
GSES 1	I can always manage to solve difficult problems if I try hard enough.	1	Not at all true
		2	Hardly true
		3	Moderately true
		4	Exactly true
GSES 2	If someone opposes me, I can find the means and ways to get what I want.	1	Not at all true
		2	Hardly true
		3	Moderately true
		4	Exactly true
GSES 3	It is easy for me to stick to my aims and accomplish my goals.	1	Not at all true
		2	Hardly true
		3	Moderately true
		4	Exactly true
GSES 4	I am confident that I could deal efficiently with unexpected events.	1	Not at all true
		2	Hardly true
		3	Moderately true
		4	Exactly true
GSES 5	Thanks to my resourcefulness, I know how to handle unforeseen situations.	1	Not at all true
		2	Hardly true
		3	Moderately true
		4	Exactly true
GSES 6	I can solve most problems if I invest the necessary effort.	1	Not at all true
		2	Hardly true
		3	Moderately true
		4	Exactly true
GSES 7	I can remain calm when facing difficulties because I can rely on my coping abilities.	1	Not at all true
		2	Hardly true
		3	Moderately true
		4	Exactly true
GSES 8	When I am confronted with a problem, I can usually find several solutions.	1	Not at all true
		2	Hardly true
		3	Moderately true
		4	Exactly true
GSES 9	If I am in trouble, I can usually think of a solution.	1	Not at all true
		2	Hardly true
		3	Moderately true
		4	Exactly true
GSES 10	I can usually handle whatever comes my way.	1	Not at all true
		2	Hardly true
		3	Moderately true
		4	Exactly true
GSES 11*	I can cope with ups and downs of my long Covid symptoms.	1	Not at all true

		2	Hardly true
		3	Moderately true
		4	Exactly true
GSES 12*	I can explain my long Covid symptoms to other people.	1	Not at all true
		2	Hardly true
		3	Moderately true
		4	Exactly true
GSES 13*	I am confident in managing the uncertainty of recovery from my long Covid symptoms.	1	Not at all true
		2	Hardly true
		3	Moderately true
		4	Exactly true
GSES 14*	If I experience new symptoms, I feel confident that I can find a way to manage them.	1	Not at all true
		2	Hardly true
		3	Moderately true
		4	Exactly true

*****The four additional context-specific questions (GSES 10-14) were generated together with people with Long Covid and represented items of most importance.

Supplementary Tables

Table S5. Breakdown of gender (self-described) and sex (assigned at birth) at baseline.

	Woman	Man	Transwoman	Nonbinary/ genderqueer/ agender/ genderfluid	Prefer not to say	Other
**Born female**	378	0	0	2	1	1
**Born male**	1	139	0	2	0	0
**Sex at birth missing**	15	4	1	0	0	0

Table S6. LISTEN complete data withdrawals by arm.

Arm	Number
Pre-randomisation	5
Intervention	7
Usual Care	3

Table S7. LISTEN partial withdrawals.

Partial withdrawals before randomisation (these participants were not progressed to randomisation)	3
Partial withdrawals after randomisation	42
Total number of partial withdrawals	45

Table S8. LISTEN partial withdrawals level and reasons by arm.

	Intervention	Usual Care
**Total number of partial withdrawals after randomisation (n=42)**	33	9
**Level of withdrawal***	**Intervention**	**Usual Care**
Withdrawal from intervention	33	0
Withdrawal from follow-up questionnaires (at 6-week & 3-month points) **	15	9
Withdrawal from qualitative interviews	20	8
**Reason for withdrawal***	**Intervention**	**Usual Care**
*Withdrawal due to patient choice****	32	9
Withdrawal due to serious adverse event (SAE)	1 ****	0
Withdrawal due to investigator's decision	0	0

*More than one level and reason of withdrawal could be selected per participant. It was not mandatory to provide an answer for each option.
**Cumulative withdrawals from questionnaires i.e. across both 6-week and 3-month time points) are presented here. Withdrawals are presented at each time point in the CONSORT flow chart.
***Participant choices for withdrawal included: Usual NHS care offered and prioritised, intervention not what expected, not randomised to the intervention, unable to prioritise sessions, symptoms too challenging to participate (including co-morbidities).
****Myocardial infarction

Table S9. LISTEN survey completion by arm.

	Intervention	Usual Care
Baseline	277	277
6 weeks*	211 (76.1%)	222 (80.1%)
3-month follow-up part 1**	210 (75.8%)	200 (72.2%)
3-month follow-up part 2***	198 (71.45%)	187 (67.5%)

*6-week follow-up CSRI and EQ-5D only
**3-month follow-up part 1 comprised Ox-PAQ, SF-12, FIS, EQ-5D-5L, GSES, Long COVID questions adapted from (55) (included in the intention to treat (ITT) analyses).
***3-month follow-up part 2 comprised AIM, IAM, FIM, PIC, CSRI

Table S10. Stage of withdrawal from follow-up questionnaire completion.

		Total	Intervention	Usual Care
**Total number of participants who withdrew from follow-up questionnaires**		24	15	9
	**Withdrew before 6-week follow-up was completed**	19	12	7
	**Withdrew after 6-week follow-up was completed**	5	3	2

Table S11. Reasons for LISTEN Intervention partial or non-adherence and summary of intervention sessions received.

**Reasons for partial or non-adherence to intervention**	**No. Intervention sessions**					**Total no. participants**
	**0**	**1**	**2**	**3**	**4***	
Withdrawal	17	6	7	2	1	33
No response or stopped responding to contact from research team/site/practitioner	16	5	5	3	0	29
Adverse event (AE)	0	0	1	0	0	1
Satisfied with support & felt no additional sessions necessary	0	0	1	2	0	3
Total	33	11	14	7	1	66*

*One participant met full intervention adherence but withdrew after completing 4 sessions.

Table S12. Adverse and Serious Adverse Event reporting by group.

	Total	LISTEN Intervention	Usual Care
**Adverse Events***	10	7 **	3
**Serious Adverse Events**	3	2 ***	1

*Only events of psychological distress or/and new/progressed psychiatric conditions were classified as an AE (where is does not meet the definition of an SAE).
** Of the 7 reported adverse events, 1 participant discontinued the intervention whilst 6 completed all sessions. The participant who discontinued the intervention was referred on to the mental health crisis team. Of the other 6 participants, 3 were referred on for additional support to the GP or psychological services.
*** Of the participants where a serious adverse event was reported, 1 participant went on to complete the full intervention.

Table S13. Serious Adverse Event categories by group.

**Serious Adverse Events**	**LISTEN Intervention**	**Usual Care**
**Reason for event being serious**		
Resulted in death	0	0
Life-threatening	1*	0
Required inpatient hospitalisation or prolongation of existing hospitalisation	1**	1
Persistent or significant disability/incapacity	0	0
Congenital anomaly/birth defect	0	0
Other medically important condition	0	0
**Causality**		
LISTEN Intervention	0	0
LISTEN assessments	0	0
Concomitant medication	0	0
Underlying disease	2	0
Other cause	0	1***
**Action taken due to SAE**		
Intervention withdrawal temporarily	0	0
Intervention stopped (withdrawal)	1	0
Intervention delayed	1	0
Intervention not changed	0	0

Unknown	0	1

*myocardial infarction
** severe headache prior to start of intervention sessions, intervention delayed but fully completed
*** impaired kidney function requiring hospital-based investigations

Table S14. Baseline characteristics of the total sample by the two groups: outcome data are missing and not missing at follow-up.

Characteristics	Total sample (n=544)<br>n (%)	Outcomes data are missing (n=134)<br>n (%)	Outcomes data are not missing (n=410)<br>n (%)
Age:			
Mean (SD)	50.0 (12.3)	46.8 (11.7)	50.1 (12.3)
Missing	1 (0.2)	1 (0.1)	0 (0.0)
Study arm:			
Usual Care	274 (50.4)	74 (55.2)	200 (48.8)
LISTEN Intervention	270 (49.6)	60 (44.8)	210 (51.2)
Gender:			
Female	394 (72.4)	93 (69.4)	301 (73.4)
Male	143 (26.3)	40 (29.9)	103 (25.1)
Other	7 (1.3)	1 (0.7)	6 (1.5)
Missing*	0 (0.0)	0 (0.0)	0 (0.0)
Ethnicity:			
White	505 (92.8)	121 (90.3)	384 (93.7)
Mixed or multiple ethnicity	15 (2.8)	6 (4.5)	9 (2.2)
Asian	15 (2.8)	4 (3.0)	11 (2.7)
Black	5 (0.9)	2 (1.5)	3 (0.7)
Other ethnicity	2 (0.4)	0 (0.0)	2 (0.5)
Missing	2 (0.4)	1 (0.7)	1 (0.2)
Living with:			
Alone	89 (16.4)	23 (17.1)	66 (16.1)
Partner	171 (31.4)	31 (23.1)	140 (34.2)
Children including adopted ones	58 (10.7)	13 (9.7)	45 (11.0)
Partner & children	160 (29.4)	44 (32.8)	116 (28.3)
Other family member	45 (8.3)	15 (11.2)	30 (7.3)
Non-family member	15 (2.8)	6 (4.5)	9 (2.2)
Missing	6 (1.1)	2 (1.5)	4 (1.0)
Dependents:			
None	349 (64.2)	81 (60.5)	268 (65.4)
Children aged ≤16	153 (28.1)	43 (32.1)	110 (26.8)
An adult reliant upon you for any support	36 (6.6)	8 (6.0)	28 (6.8)
Missing	6 (1.1)	2 (1.5)	4 (1.0)
Highest level of qualification:			
No qualifications	12 (2.2)	3 (2.2)	9 (2.2)
1-4 GCSEs or equivalent	39 (7.2)	16 (11.9)	23 (5.6)
5+ GCSEs or equivalent	50 (9.2)	16 (11.9)	34 (8.3)
Apprenticeship	4 (0.7)	0 (0.0)	4 (1.0)
2+ A Levels or equivalent	73 (13.4)	21 (15.7)	52 (12.7)
Degree level or above	343 (63.1)	75 (56.0)	268 (65.4)

Other qualifications	17 (3.1)	2 (1.5)	15 (3.7)
Missing	6 (1.1)	1 (0.7)	5 (1.2)
Employment status:			
In full time education	28 (5.2)	10 (7.5)	18 (4.4)
In part time education	7 (1.3)	5 (3.7)	2 (0.5)
House person	13 (2.4)	4 (3.0)	9 (2.2)
Employed (full time)	230 (42.3)	63 (47.0)	167 (40.7)
Employed (part time)	121 (22.2)	23 (17.2)	98 (23.9)
Unemployed	58 (10.7)	15 (11.2)	43 (10.5)
Retired	82 (15.1)	13 (9.7)	69 (16.8)
Missing	5 (0.9)	1 (0.7)	4 (1.0)
In the past 3 months, use of any community-based health and social work services:			
Yes	111 (20.4)	24 (17.9)	87 (21.2)
No	425 (78.1)	108 (80.6)	317 (77.3)
Missing	8 (1.5)	2 (1.5)	6 (1.5)
In the past 3 months, use of any community-based mental health services:			
Yes	64 (11.8)	19 (14.2)	45 (11.0)
No	470 (86.4)	113 (84.3)	357 (87.1)
Missing	10 (1.8)	2 (1.5)	8 (2.0)
Positive Covid test:			
Yes	479 (88.0)	114 (85.1)	365 (89.0)
No	65 (12.0)	20 (14.9)	45 (11.0)
Missing	0 (0.0)	0 (0.0)	0 (0.0)
Number of Long Covid symptoms:			
Mean (SD)	11.5 (3.6)	11.8 (3.6)	11.4 (3.6)
Missing	0 (0.0)	0 (0.0)	0 (0.0)

Table S15. Sensitivity analysis* using multiple imputation for missing observations and comparison of the outcomes between the study arms (N=544).

Outcomes	Adjusted effect estimates β (95% CIs)[a] from Table 4	p-values	Sensitivity analysis*: Adjusted effect estimates β (95% CIs) [b]	p-values
Primary outcome: Ox-PAQ routine activities domain score	-2.90 (-5.66, -0.15)	0.039	-2.80 (-5.53, -0.24)	0.032
Secondary outcomes:				
Ox-PAQ emotional wellbeing domain score	-5.89 (-8.99, -2.79)	<0.001	-6.21 (-9.29, -3.13)	<0.001
Ox-PAQ social engagement domain score	-2.81 (-6.19, 0.57)	0.103	-2.30 (-5.51, 0.91)	0.160
FIS scores:				
Cognitive dimension	-2.34 (-3.56, -1.12)	<0.001	-2.36 (-3.53, -1.18)	<0.001
Physical dimension	-1.80 (-2.93, -0.67)	0.002	-1.68 (-2.76, -0.60)	0.001
Social dimension	-4.63 (-6.81, -2.45)	<0.001	-4.37 (-6.54, -2.19)	<0.001
Overall score	-8.65 (-12.79, -4.52)	<0.001	-8.46 (-12.90, -4.03)	<0.001
EQ-5D-5L scores:				
Index score	0.04 (0.00, 0.07)	0.046	0.04 (0.00, 0.07)	0.058
VAS score	2.72 (-0.80, 6.24)	0.130	2.49 (-0.81, 5.79)	0.139
GSES scores:				
Original 10 items scale	1.42 (0.54, 2.30)	0.002	1.55 (0.68, 2.42)	0.001
Covid 4 items scale	1.38 (0.93, 1.82)	<0.001	1.40 (0.93, 1.86)	<0.001
Original scale with Covid 4 items overall score	2.79 (1.66, 3.93)	<0.001	2.88 (1.69, 4.06)	<0.001
SF-12 scores:				
Physical health	0.48 (-0.74, 1.71)	0.440	0.64 (-0.57, 1.86)	0.299
Mental health	2.85 (1.23, 4.46)	0.001	2.82 (1.19, 4.45)	0.001

β: Regression coefficients (difference of mean outcome scores at three months follow-up between the study arms) adjusted for baseline outcome scores (≈ difference of mean outcome scores change in the study arms from baseline to three months follow-up)
95% CIs: 95% confidence intervals
Ox-PAQ: Oxford Participation and Activities Questionnaire
SF-12: Short Form-12 items version 1
FIS: Fatigue Impact Scale
EQ-5D-5L: EuroQol Group health related quality of life questionnaire
VAS: Visual Analogue Scale
GSES: Generalised Self-Efficacy Scale
[a] Effect estimates (95% CI) from linear mixed effect models with the outcome scores at three months follow-up as dependent variable and study arm and baseline outcome scores as independent variable, adjusted for the random effect of site and fixed effects of age, gender, ethnicity, employment status and the number of long Covid symptoms at baseline
***Sensitivity analysis using imputation for missing observations:**
[b] Effect estimates from the analysis based on multiple imputation for missing observations with the assumption of missingness at random (MAR). Missing observations were replaced by the imputed values using chained equations of linear regression. In the imputation equation for each outcome at follow-up, baseline variables of the study arms, site, age, gender, ethnicity, employment status, qualification, the number of long Covid symptoms and baseline outcome were used as independent variables. Twenty imputed datasets were created for the imputation of each outcome and the imputation-specific estimates were obtained from a similar model as described in [a] for the effect of intervention on the primary and secondary outcomes. The estimates were combined using Rubin's rules.

References.

1. Morley D, Dummett S, Kelly L, et al. Measuring improvement in health-status with the Oxford Participation and Activities Questionnaire (Ox-PAQ). *Patient Related Outcome Measures* 2019:153-56.
2. Joly F, Kosinski M, Shafer F, et al. Clinically important change in SF-12v2 physical (PCS) and mental (MCS) component summary scores for patients with cold agglutinin disease: an analysis using the phase 3 CARDINAL and CADENZA studies. *Blood* 2021;138:2002.
3. Rendas-Baum R, Yang M, Cattelin F, et al. A novel approach to estimate the minimally important difference for the Fatigue Impact Scale in multiple sclerosis patients. *Quality of Life Research* 2010;19:1349-58.
4. McClure NS, Al Sayah F, Xie F, et al. Instrument-defined estimates of the minimally important difference for EQ-5D-5L index scores. *Value in Health* 2017;20(4):644-50.
