# Supplementary material for: Effectiveness of a personalised self-management intervention for people living with long covid (Listen trial): pragmatic, multicentre, parallel group, randomised controlled trial
Source: BMJ Med. 2025 Jan 31;4(1):e001068. doi: 10.1136/bmjmed-2024-001068 (PMC11881025; doi:10.1136/bmjmed-2024-001068)
Supplement: online supplemental file 3 [file bmjmed-4-1-s003.pdf]

Listen

LONG COVID PERSONALISED SELF-MANAGEMENT SUPPORT EVALUATION

(LISTEN TRIAL)

PROTOCOL VERSION 6.0

DATED 10.03.2023

**Sponsor:**	**Kingston University**
**Sponsor ref:**	
**Funder:**	NIHR [Long COVID in non-hospitalised individuals Call]
**Funder ref:**	COV-LT2-0009
**REC ref:**	21/WA/0368
**IRAS number:**	306220
**ISRCTN ref:**	36407216
**Q-Pulse Document Template Number:**	**TPL/003/2**

SIGNATURE PAGE

The undersigned confirm that the following protocol has been agreed and accepted and that the Chief Investigator agrees to conduct the trial in compliance with the approved protocol and will adhere to the principles outlined in the relevant trial regulations, Good Clinical Practice (GCP) guidelines, and Centre for Trial Research's (CTR's) Standard Operating Procedures (SOPs).

I agree to ensure that the confidential information contained in this document will not be used for any other purpose other than the evaluation or conduct of the clinical investigation without the prior written consent of the Sponsor.

I also confirm that I will make the findings of the trial publicly available through publication or other dissemination tools without any unnecessary delay and that an honest accurate and transparent account of the trial will be given; and that any discrepancies from the trial as planned in this protocol will be explained.

**Co-Chief Investigator - Kingston and St George's Joint Faculty Health, Social Care and Education**			
**Name**	**Position**	**Signature**	**Date**
**Co-Chief Investigator – Cardiff University**			
**Name**	**Position**	**Signature**	**Date**

General Information This protocol describes the LISTEN clinical trial, and provides information about the procedures for entering participants into the trial. The protocol should not be used as a guide, or as an aide-memoire for the treatment of other participants. Every care has been taken in drafting this protocol; however, corrections or amendments may be necessary. These will be circulated to the known Investigators in the trial. Problems relating to the trial should be referred, in the first instance, to CTR.

Contact details – Chief Investigator/s & Co-Investigator/s

CHIEF INVESTIGATOR

Professor Fiona Jones

Position: Professor of Rehabilitation Research

Institution: Faculty of Health Social Care and Education

St George's University of London and Kingston University

SW17 0RE

E-mail : f.jones@sgul.kingston.ac.uk

CHIEF INVESTIGATOR

Professor Monica Busse

Position: Director of Mind Brain Neuroscience Trials

Institution: Centre for Trials Research

Cardiff University

CF144XN

E-mail : busseme@cardiff.ac.uk

CO-INVESTIGATORS

Professor Nick Sevdalis

Position: Professor of Implementation Science and Patient Safety

Institution: Kings College London

E-mail : nick.sevdalis@kcl.ac.uk

Professor Aloysius Niroshan Siriwardena

Position: Professor of Primary & Pre-Hospital Health Care

Institution: University of Lincoln

E-mail: nsiriwardena@lincoln.ac.uk

Professor Adrian Edwards

Position: Professor of General Practice. Director of PRIME Centre Wales and Wales Covid-19 Evidence Centre

Institution: Cardiff University

E-mail : EdwardsAG@cardiff.ac.uk

Dr Natalie Joseph-Williams

Position: Senior Lecturer in Improving Patient Care

Institution: Cardiff University

E-mail : JosephNJ1@cardiff.ac.uk

Dr Philip Pallmann

Position: Deputy Director of Research Design and Conduct Service and Research Fellow (Statistician)

Institution: Cardiff University

E-mail: PallmannP@cardiff.ac.uk

Dr Bernadette Sewell

Position: Senior Lecturer, Public Health, Policy and Social Sciences (Health Economist)

Institution: Swansea University

E-mail: b.diethart@swansea.ac.uk

Dr Jackie McRae

Position: Associate Professor, Director of Research and Consultant Speech and Language Therapist (UCLH)

Institution: Centre for Allied Health. Kingston University

E-mail : J.Mcrae@sgul.kingston.ac.uk

Dr Jessica Fish

Position: Clinical Psychologist

Institution: Clinical Neuropsychology & Health Psychology, St George's University Hospitals NHS Foundation Trust

E-mail : Jessica.Fish@stgeorges.nhs.uk

Dr Rachel Lowe

Position : Senior Trial Manager

Institution: Cardiff University

E-mail : LoweRS@cardiff.ac.uk

Miss Maria Ines de Sousa de Abreu

Position: Public and Patient Involvement (PPI) Representative.

**SPONSOR contact details:**
Title and name: Professor Priscilla Harries
Position: Associate Dean Research & Business Innovation
Institution: Kingston University
E-mail : P.Harries@kingston.ac.uk

Trial Co-ordination:

The LISTEN trial is being coordinated by the Centre for Trials Research (CTR), Cardiff University, a Clinical Research Collaboration (UKCRC) registered trials unit.

This protocol has been developed by the LISTEN Trial Management Group (TMG)

For **all queries** please contact the LISTEN team through the main trial email address. Any clinical queries will be directed through the Trial Manager to either the Chief Investigator or a Co-Investigators

**Main Trial Email:**	LISTEN@cardiff.ac.uk
Trial Administrator:	Ms Mandy Ilses
Trial Manager:	Ms Claire Potter
Data Manager:	Ms Christy Barlow
Senior Trial Statistician:	Dr Philip Pallmann
Trial Statistician	Dr Muhammad Riaz
Senior Trial Manager:	Dr Rachel Lowe
Health Economist:	Dr Bernadette Sewell
Director:	Professor Monica Busse
Research Associate:	Dr Ishrat Islam
Research Associate:	Dr Anna Torrens Burton
Qualitative Research Associate:	Dr Fiona Leggat

Randomisations:

Randomisation
All participants who have passed screening and eligibility checks will be randomised by the central study team from within the study database (See section 9.5 for more details).

Clinical queries:

Clinical queries
All clinical queries will be directed to the most appropriate clinical person.
**Contact details for any clinical queries:** LISTEN@cardiff.ac.uk

Serious Adverse Events (SAE):

SAE reporting
Where the adverse event meets one of the serious categories, an SAE form should be completed in the study database by the responsible clinician within 24 hours of becoming aware of the event (See section 16 for more details).
**Contact details for any SAE reporting:** LISTEN@cardiff.ac.uk

Table of Contents

1 Amendment History 5
2 Synopsis 7
3 Trial Summary & Schema 11
3.1 Trial Schema 11
3.2 Participant Flow Diagram 12
Trial Lay Summary 13
4 Background 13
4.1 The rationale for Current Trial/Justification of Treatment Options 14
5 Trial Objectives/Endpoints and Outcome Measures 16
5.1 Primary Objectives 16
5.2 Secondary Objectives 16
5.3 Exploratory Objectives 16
5.4 Primary Outcomes Measure(s) 16
5.5 Secondary Outcomes Measure(s) 17
5.6 Exploratory Outcome Measure(s) 18
6 Trial Design and Setting 18
6.1 Risk Assessment 19
7 Site and Investigator Selection 20
8 Participant Selection 20
8.1 Inclusion Criteria 21
8.2 Exclusion Criteria 21
9 Recruitment, Screening and Registration 21
9.1 Participant Identification 21
9.2 Screening Logs 24
9.3 Recruitment Rates 24
9.4 Informed Consent 24
9.5 Registration and Randomisation 25
9.5.1 Registration 25
9.5.2 Randomisation 25
10 Withdrawal & Lost to Follow-up 26
10.1 Withdrawal 26
10.2 Lost to Follow up 26
11 Trial Intervention 27
11.1 The 'LISTEN' Personalised Self-Management Support Intervention 27
11.2 Comparator 27
12 Trial Procedures 28
12.1 Internal Pilot 28
12.2 Staff Training and Assessment of Intervention Fidelity of Delivery 28
12.3 Assessments 29
12.3.1 Baseline 29
12.3.2 Follow-up 29
12.3.3 Mixed Methods Process Evaluation 31
13 Safety Reporting 32
13.1 Definitions 32
13.2 Trial Specific AE Reporting Requirements 33
13.3 Trial Specific SAE Reporting Requirements 33
13.4 SAE Causality 34
13.5 SAE Expectedness 35
13.6 SAE Reporting Procedures 35
13.6.1 SAE Participating Site Responsibilities 35
13.6.2 The CTR Responsibilities 36

13.7 Contraception and Pregnancy ... 36
13.8 Urgent Safety Measures (USMs) ... 36
14 Statistical Considerations ... 37
14.1 Randomisation ... 37
14.2 Blinding ... 37
14.3 Sample Size ... 37
14.4 Missing, Unused & Spurious Data ... 37
14.5 Procedures for Reporting Deviation(s) from the Original SAP ... 37
14.6 Termination of the Trial ... 37
14.7 Inclusion in Analysis ... 37
15 Analysis ... 38
15.1 Main Analysis ... 38
15.1.1 Sub-Group & Interim Analysis ... 38
15.2 Qualitative Analysis ... 38
15.3 Cost-Effectiveness Analysis ... 39
16 Data Management ... 40
16.1 Data Collection ... 42
16.2 Completion of CRFs ... 42
16.2.1 Paper CRFs ... 42
16.2.2 Electronic CRFs ... 42
17 Translational Research or Sub-Trial ... 43
18 Protocol/GCP Non-Compliance ... 43
19 End of Trial Definition ... 43
20 Archiving ... 43
21 Regulatory Considerations ... 44
21.1 Ethical and Governance Approval ... 44
21.2 Data Protection ... 44
21.3 Indemnity ... 44
21.4 Trial Sponsorship ... 45
21.5 Funding ... 45
22 Trial Management ... 45
22.1 TMG (Trial Management Group) ... 45
22.2 TSC (Trial Steering Committee) ... 45
23 Quality Control and Assurance ... 46
23.1 Monitoring ... 46
23.2 Audits & inspections ... 46
24 Publication policy ... 46
25 Milestones ... 46
26 References ... 47

Glossary of abbreviations

**ABPI**	Association of the British Pharmaceutical Industry
**AE**	Adverse Event
**AIM**	Acceptability of Intervention Measure
**AR**	Adverse Reaction
**CF**	Consent Form
**CFIR**	Consolidated Framework for Implementation Research
**CI**	Chief Investigator
**CRF**	Case Report Form
**CSRI**	Client Service Receipt Inventory
**CTR**	Centre for Trials Research
**CTU**	Clinical Trials Unit
**CU**	Cardiff University
**DMC**	Data Monitoring Committee
**DSCHR**	Division of Social Care and Health Research
**EQ-5D-5L**	EuroQol Five Dimensions Five Levels Quality of Life Questionnaire
**FDA**	Food and Drug Administration
**FIS**	Fatigue Impact Scale
**FIM**	Feasibility of Intervention Measure
**GAfREC**	Governance Arrangements for NHS Research Ethics Committees
**GCP**	Good Clinical Practice
**GP**	General Practitioner
**GSES**	Generalised Self-Efficacy Scale
**HB**	Health Board
**HE**	Health Economics
**HRA**	Health Research Authority
**HTA**	Health Technology Assessment
**IAM**	Intervention Appropriateness Measure
**IC**	Informed consent
**ICC**	Intraclass Correlation
**ICF**	International Classification of Functioning, Disability and Health
**ICH**	International Conference on Harmonization
**IDMC**	Independent Data Monitoring Committee
**IEC**	Independent Ethics Committee

**IRAS**	Integrated Research Application System
**IRB**	Institutional Review Board (IRB)
**ISF**	Investigator Site File
**ISRCTN**	International Standard Randomised Controlled Trial Number
**IT**	Information technology
**MICD**	Minimum clinically important difference
**MRC**	Medical Research Council
**NHS**	National Health Service
**NICE**	National Institute for Clinical Excellence
**NIHR**	National Institute for Health Research
**NRR**	National Research Register
**Ox-PAQ**	Oxford Participation and Activities Questionnaire
**PCT**	Primary Care Trust
**PCU**	Permissions Coordinating Unit
**PI**	Principal Investigator
**PIAG**	Participant Information Advisory Group
**PIC**	Participant Identification Centre
**PIS**	Participant Information Sheet
**PPI**	Public and Patient Involvement
**QA**	Quality Assurance
**QALY**	Quality-Adjusted Life Years
**QL (QoL)**	Quality of Life
**R&D**	Research and Development
**RCT**	Randomised Controlled Trial
**REC**	Research Ethics Committee
**RGF**	Research Governance Framework for Health and Social Care
**SAE**	Serious Adverse Event
**SAGE**	Scientific Advisory Group for Emergencies
**SAP**	Statistical Analysis Plan
**SF-12**	Short Form (12) Health Survey
**SOP**	Standard Operating Procedure
**SSA**	Site Specific Assessment
**TMF**	Trial Master File
**TMG**	Trial Management Group
**TOC**	Table of Content

**TSC**	Trial Steering Committee
**TSF**	Trial Site File
**UC**	Usual Care
**UKCRC**	UK Clinical Research Collaboration
**USM**	Urgent Safety Measures
**WHO**	World Health Organisation
**WOCBP**	Women of Child Bearing Potential

1 Amendment History

The following amendments and/or administrative changes have been made to this protocol since the implementation of the first approved version.

Amendment No.	Protocol version no.	Date issued	Summary of changes made since the previous version
Substantial Amendment 11	6.0	10/03/2023	- **9.1 Participant Identification –** this section has been updated to include follow up phonecalls from site teams to participants sent LISTEN recruitment information by text messages or mailouts.
Substantial Amendment – 09	5.0	23/01/2023	- **9.1 Participant Identification –** this section has been updated to include text messages as a method for advertising the lISTEN study to waiting lists/PIC search records.
Non-substantial Amendment – 03	4.0	28-11-2022	- **Update to sponsor details**<br>- **Update to Trial Statistician**<br>- **Section 8.2 Exclusion Criteria** the exclusion criteria for progressive or palliative condition has been amended due to feedback from participants that is what not clear.<br>- **9.2 Participant Identification** the use of videos for recruitment has been added<br>- **12.1 Internal Pilot.** The internal pilot dates and targets have been updated in line with a change request to funder
Substantial Amendment - 02	3.0	29-03-2022	**Section 3.2** Participant Flow Diagram has been updated. Process evaluation questionnaires removed from baseline, added originally in error.<br><br>Site locations throughout protocol have been amended to UK.<br><br>**9.1 Participant Identification** information added to allow central CTR team to conduct over the phone eligibility reviews and consenting, as well as sites.

			Process for completing expression or interest form has been updated due to a change in database programme.<br>Process for completing consent and questionnaires has been changed from logging into the database to email based links.<br>**9.5.2 Randomisation** The method of randomisation has been changed from minimisation to stratification by site.<br>**10.1 Withdrawal** Process for completing withdrawal of consent form changed to link sent through an email.<br>**14.1 Randomisation** The method of randomisation has been changed, within the statistical section, from minimisation to stratification by site.<br>**16 Data Management Source Data Table** paper consent forms added and source data for expression of interest andeligibility updated.<br>**16.2.2 Electronic CRFs** The programme used to build the LISTEN database has been changed to REDCap with additional surveys on Jisc Online Surveys.
**Substantial Amendment - 01**	2.1	20-12-2021	Sponsor name changed to Kingston University

List summary of protocol amendments here whenever a new version of the protocol is produced. Ensure details are also updated in a full protocol change log.

2 Synopsis

**Short title**	Long Covid PersonalIsed Self-managemenT support EvaluatioN
**Acronym**	LISTEN
**Internal ref. no.**	
**Development phase**	Phase III
**Funder and ref.**	NIHR COV-LT2-0009
**Trial design**	Individually randomised two-arm controlled trial with internal pilot and mixed-methods process evaluation
**Trial participants**	Individuals with long Covid, ≥18 years, English or Welsh speaker or who have access to someone who can act as a translator
**Planned sample size**	558 individuals living with long Covid
**Planned number of sites**	24 research sites across the UK
**Inclusion criteria**	Participants will be eligible if they experience persistent illness (at least one long Covid symptom for 12 weeks or longer) AND meet any one of the following criteria:<br>(1) Positive SARS-CoV-2 PCR or antigen test (positive Covid-19 test) during the acute phase of illness;<br>(2) Positive SARS-CoV-2 antibody test (positive Covid-19 antibody test) at any time point in the absence of SARS-CoV-2 (Covid-19) vaccination history;<br>(3) Loss of sense of smell or taste during the acute phase in the absence of any other identified cause;<br>(4) Symptoms consistent with SARS-CoV-2 (Covid-19) infection during the acute phase and high prevalence of Covid-19 at time and location of onset;<br>(5) at least one symptom consistent with SARS-CoV-2 (Covid-19) infection during the acute phase AND close contact of a confirmed case of Covid-19 around the time of onset.<br>They will be aged 18 years or above and be an English or Welsh speaker or have access to someone who can act as a translator.<br>They must have consulted with their GP to rule out serious complications or the need for further investigation in relation to persistent symptoms following Covid-19 infection.

**Exclusion criteria**	Participants will not be eligible if they are recieving palliative or end of life care or have been hospitalised for treatment of Covid-19 symptoms, during the acute phase of Covid illness, or are currently participating in any Covid intervention trial (including contributing to the LISTEN co-design activities).
**Treatment duration**	Up to 6 (1 hour) coaching sessions over 10 weeks
**Follow-up duration**	3 months with consent for contact for long term follow up
**Planned trial period**	24 months
**Primary objective**	To evaluate the impact of the LISTEN co-designed personalised self-management support intervention on routine activities as assessed by the routine activities domain of the Oxford Participation and Activities Questionnaire (Ox-PAQ).
**Secondary objectives**	i. To evaluate the impact of the LISTEN intervention on emotional well-being as assessed by the relevant domain sub-scale of the Ox-PAQ.<br>ii. To evaluate the impact of the LISTEN intervention on social engagement as assessed by the relevant domain sub-scale of the Ox-PAQ.<br>iii. To evaluate the impact of the LISTEN intervention on health-related quality of life as assessed by the Short Form (12) Health Survey.<br>iv. To evaluate the impact of the LISTEN intervention on fatigue as measured by the Fatigue Impact Scale (FIS).<br>v. To assess health-related quality of life expressed as utility using the EQ-5D-5L questionnaire.<br>vi. To gather information on healthcare resource use using an adapted client service receipt inventory.<br>vii. To assess the cost-effectiveness of the LISTEN intervention.<br>viii. To explore key anticipated mediators of intervention outcome (namely self-efficacy in the context of Covid-19) using the generalised self-efficacy scale (GSES) with additional context-specific questions.<br>ix. To conduct a theory-driven detailed process evaluation within the trial using validated implementation scales to assess intervention acceptability, appropriateness and feasibility (through use of the AIM, IAM and FIM questionnaires, respectively) for intervention users and providers.

**Exploratory objectives**	x. To explore issues relating to the context, mechanisms and outcomes of the intervention and how they may differ to usual care through qualitative interviews in a sub-set of participants who have received either the LISTEN intervention or usual care and focus groups with practitioners.
**Primary outcome**	The primary outcome will be the change in routine activities. It will be measured by the routine activities domain of the Ox-PAQ.
**Secondary outcomes**	Secondary outcomes of intervention effect will include the change in emotional well-being, social engagement and quality of life. These will be measured by the Emotional well-being and Social Engagement sub-scales of the Ox-PAQ; the SF-12; FIS, EQ-5D-5L; GSES.<br>Furthermore, we will examine the intervention cost and changes in healthcare resource use and cost as a result of the intervention using an adapted Client Service Receipt Inventory (CSRI).<br>Intervention process-related secondary outcomes will be the perceptions of acceptability, appropriateness and feasibility of the use and implementation of the intervention. These will be measured by the Acceptability of Intervention Measure (AIM); Intervention Appropriateness Measure (IAM); and Feasibility of Intervention Measure (FIM) tool/ questionnaire in both participants and in intervention providers (practitioners).
**Exploratory outcomes**	The exploratory outcomes will be the perceptions of the participants and the intervention providers on the following topics:<br>i. Acceptability and feasibility of trial processes (randomisation, outcomes measures etc).<br>ii. Acceptability and usability of co-design resources – book and digital.<br>iii. Acceptability of the intervention training.<br>iv. Acceptability and feasibility of one-to-one coaching sessions<br>v. Skills required to deliver the intervention and alignment with intervention fidelity.<br>vi. Indicators of intervention success (ways in which participants and intervention providers recognise a successful coaching interaction or outcome) and the extent to which they match to outcomes of

	importance to participants with long Covid and those delivering the intervention.<br>vii. Processes that facilitate and/or act as barriers to implementation , including contextual factors such as organisational, personal or professional issues.<br>viii. Factors required to enable sustainability and spread to other NHS sites beyond the project timeline.
**Intervention**	A personalised self-management support intervention consisting of practitioner delivered one-to-one coaching sessions to support everyday activities and living with long Covid symptoms (up to 6 sessions delivered remotely online or via telephone) and access to co-designed resources.
**Comparator**	Usual care as currently available in the NHS.

3 Trial Summary & Schema

3.1 Trial Schema

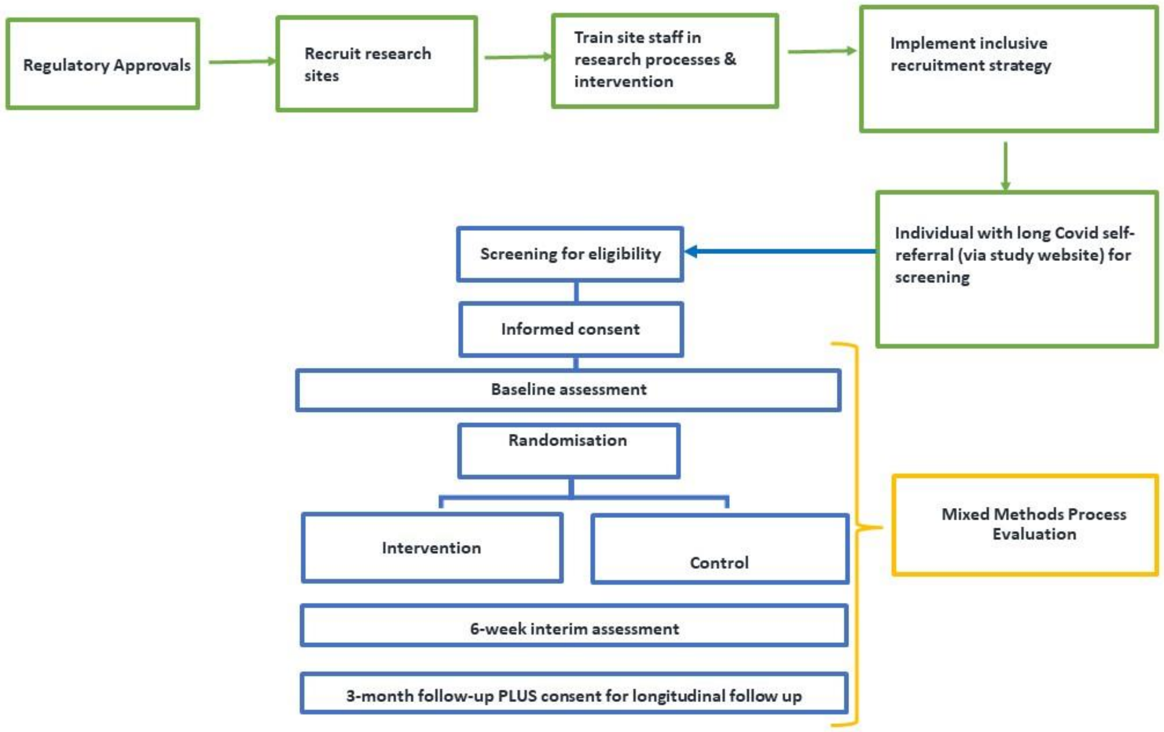

3.2 Participant Flow Diagram

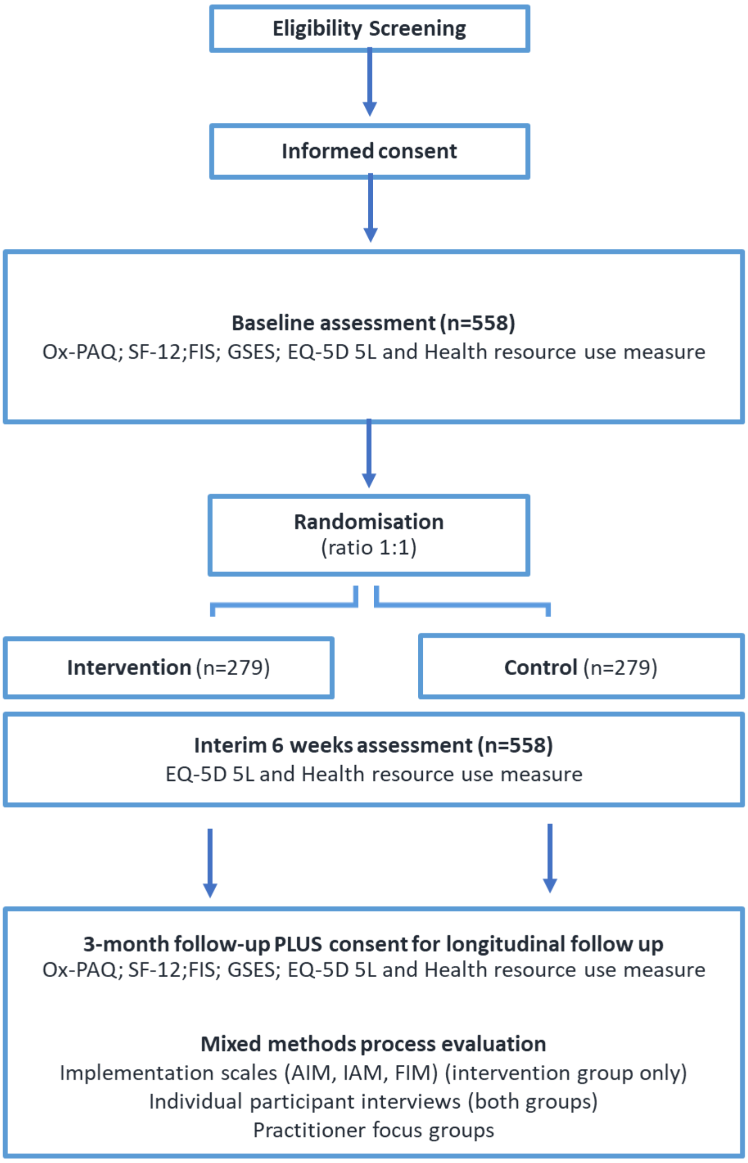

Trial Lay Summary

Individuals with long Covid experience a wide variety of ongoing problems such as tiredness and difficulty with everyday tasks and means they can struggle to return to their former lives. This is then made worse by uncertainty and a lack of understanding by some healthcare professionals.

The LISTEN project is evaluating a package of self-management support, co-designed with individuals living with long Covid, to ensure that the intervention can be personalised to individual needs.

We will recruit individuals with long Covid and randomly allocate them to an intervention or control group. The control group will continue with their usual care and if requested, the LISTEN team will signpost them to long Covid care pathways in their regions; the intervention group will receive the new resources and up to six coaching sessions from the trained rehabilitation practitioners. We will estimate the cost of the intervention and test its effect on how participants feel and cope with everyday activities. We will record healthcare resource use, expenses and time of work to understand the economic impact of long Covid and our intervention on society and individuals. We will explore ways in which the intervention can be used across communities. Individuals living with long Covid and a large PPI panel from diverse backgrounds have helped shape the LISTEN project. With our co-design group and inclusion advisor we will recruit a PPI panel co-chaired by our PPI co-applicant. We will share our findings through accessible communications designed with our PPI group, academic publications and conferences.

4 Background

Long Covid is the name 'collectively made' by individuals to bring notice to persistent and complex Covid symptoms[1], with a reported prevalence of 205 different symptoms commonly including 10 organ systems. Fatigue, and cognitive dysfunction are the most common, and exercise, mental activity and stress are important triggers[2]. Long Covid is estimated to affect at least 10% of individuals with a positive Covid-19 test, although this is an underestimation as many in the early months of the pandemic were never tested[3].

An NIHR themed review stated that 'long Covid is a significant health burden unlikely to be met by existing NHS services' [4] . The potential for a lasting legacy of long Covid is serious, with a high incidence of individuals experiencing symptoms for >28 days, not returned to work by six months and continuing to experience significant symptom burden[2]. However, the impact extends beyond symptoms and includes the social impact of stigmatising attitudes of healthcare professionals. This risks repetition of similar mistakes and moral judgements to those made with Myalgic Encephalomyelitis (ME) or

Functional Neurological Disorders (FND)[1 5 6]. There is the potential to experience the same inadequate system of 'revolving door' healthcare which despite high prevalence, severity, costs and growing evidence have poorly accessible, geographically patchy, inequitable care provision[7]. Furthermore, paternalistic, overly medicalised management of long Covid could directly contribute to poor long-term clinical outcomes, with a cycle of costly and unnecessary re-evaluations, re-investigations and inappropriate treatments[8 9].

The uncertainty and confusion of long Covid with varied, relapsing and remitting symptoms are compounded by a heavy sense of loss and stigma[3]. The lack of a clear diagnosis has parallels with other poorly understood and medically unexplained conditions which increases the risk of individuals with long Covid feeling misunderstood and overlooked by healthcare professionals and services, respectively[10]. Reports of individuals experiencing wide-ranging and serious symptoms, dismissed with the label of 'anxiety' is concerning given the amount of 'unknowns' including symptoms, not obviously post viral, appearing later[11].

Respiratory interventions or physical activity pacing may seem intuitive intervention options[12]. However, focussing solely on one symptom risks ignoring the biopsychosocial impact of multiple interacting and fluctuating symptoms on everyday life and routine activities[13]. In the absence of definitive evidence to guide appropriate rehabilitation interventions there is the opportunity to extrapolate learning from personalised models such as self-management support with positive outcomes for people living with multiple and complex conditions [14]. Such interventions accommodate heterogeneity and the need for individualised treatment plans and are also advocated by long Covid patient groups [15]. Indeed, a review of existing evidence provides support for a self-management approach aimed at the diverse needs of individuals with long Covid [14]. Such interventions could provide a positive impact over a relatively short duration[16], contrary to underpinning principles of many rehabilitation interventions that emphasise 'more is better' [17].

4.1 The rationale for Current Trial/Justification of Treatment Options

Evidence for self-management interventions emphasise the need for contextualisation to 1) specific challenges and complexity of the condition 2) understanding the setting (community healthcare) and 3) training which addresses knowledge, skills and attitudes required by practitioners [18,19] and the adoption of specific language and techniques to support key self-management skills such as problem-solving, reflection and personalised goal setting [20].

The key aim of this randomised two-arm trial is to evaluate the effectiveness and cost-effectiveness of a personalised self-management support intervention for non-hospitalised people living with long Covid. The LISTEN intervention is personalised self-management support intervention that draws on

evidence from Bridges Self-management theoretically informed by self-efficacy as the most successful foundation for self-management programmes [14],[21] [20]. In this approach, therapeutic interactions become less directive and more collaborative, facilitating individuals' problem solving, goal mastery and building self-efficacy [22]. It will also incorporate new emerging evidence, for example, that a 'one-size fits all' graded exercise programme is unlikely to be of use [13], and that the relative risk of developing psychiatric and neurological disorders post-Covid is markedly higher than the general population[23]. Throughout this document, when we describe the 'LISTEN intervention' we are referring to the aforementioned personalised self-management support intervention.

The LISTEN intervention will be evaluated in terms of impact on participation in routine activities, emotional well-being, social participation, fatigue and self-efficacy. Impact on quality of life and cost-effectiveness will also be evaluated. Information about the study will be made available to participants through a variety of sources including NHS, third sector settings and community support groups and interested individuals with long Covid will be able to self-refer into the trial. The primary outcome time-point is at three month following randomisation however consent will be obtained for longer-term follow-up (beyond the length of the funded evaluation). An internal pilot will assess site opening and recruitment. Intervention acceptability and feasibility will be measured as part of the embedded mixed-methods process evaluation, and enable a detailed analysis of implementation enablers and barriers to adoption and sustainability beyond the project timeline. This work will inform and deliver a national implementation support package (for example training programme for rehabilitation teams, web platform, training manuals etc.) ready for scale-up and implementation by the end of the project.

5 Trial Objectives/Endpoints and Outcome Measures

5.1 Primary Objectives

Our primary objective is to evaluate the impact of the LISTEN co-designed personalised self-management support intervention on routine activities as assessed by the routine activities' domain of the Oxford Participation and Activities Questionnaire (Ox-PAQ).

5.2 Secondary Objectives

Secondary objectives are:

i. To evaluate the impact of the 'LISTEN intervention' on emotional well-being as assessed by the relevant domain sub-scale of the Ox-PAQ.

ii. To evaluate the impact of the 'LISTEN intervention' on social engagement as assessed by the relevant domain sub-scale of the Ox-PAQ.

iii. To evaluate the impact of the 'LISTEN intervention' on health-related quality of life as assessed by the Short Form-12 - Health Survey.

iv. To evaluate the impact of the 'LISTEN intervention' on fatigue as measured by the Fatigue Impact Scale.

v. To gather information on utility (using the EQ-5D-5L questionnaire) and health care resource use (using an adapted Client Service Receipt Inventory).

vi. To assess the cost-effectiveness of the 'LISTEN intervention'.

vii. To explore key anticipated mediators of intervention outcome (namely self-efficacy in the context of Covid-19) using the generalised self-efficacy scale (GSES) with additional context-specific questions.

viii. To conduct a theory-driven detailed process evaluation within the trial using validated implementation scales to assess intervention acceptability, appropriateness and feasibility.

5.3 Exploratory Objectives

Additional exploratory objectives are to understand issues relating to the context, mechanisms and outcomes of the intervention and how they may differ to usual care through qualitative interviews in a sub-set of participants who have received either the LISTEN intervention or usual care and focus groups with practitioners.

5.4 Primary Outcomes Measure(s)

The trial primary outcome measure is the routine activities scale domain of the Oxford Participation and Activities Questionnaire (Ox-PAQ). The Ox-PAQ is a 23-item, fully validated patient reported outcome measure developed specifically to assess participation and activity in individuals with chronic

health problems. Ox-PAQ items have been generated using the World Health Organisation (WHO) International Classification of Functioning, Disability and Health (ICF) as a theoretical framework. Participation is reflected across three domains, namely Routine Activities (14 items), Emotional Well-Being (5 items) and Social Engagement (4 items), all of which demonstrate sound psychometric properties in terms of validity, reliability and sensitivity to change[24]. The minimum clinically important difference (MCID) for the routine activities sub-scale is 7.1 corresponding to an effect size of 0.32.

5.5 Secondary Outcomes Measure(s)

i. Emotional well-being is evaluated in 5 items relating to feeling of control over life and feeling of sadness, anxiety, stress and depression in the past 4 weeks. The MCID for the emotional well-being sub-scale is 10.77 with an effect size of 0.44[24].

ii. Social engagement is measured in 4 items namely difficulties in maintaining the friendship, engaging with people, engaging in community life and communicating with others. The MCID for the social engagement sub-scale is 5.47 with an effect size of 0.28[24].

iii. The Short Form-12 (SF-12) Health Survey[25], a 12-item, patient-reported survey of patient health, will facilitate an in-depth exploration of physical, social and emotional domains relevant to health-related quality of life.

iv. The Fatigue Impact Scale[26] (FIS) will provide a detailed understanding of the impact of fatigue on cognitive, physical and psychosocial functioning in daily living.

v. The generalised self-efficacy scale[27] (GSES) {http://userpage.fu-berlin.de/%7Ehealth/engscal.htm)} assesses perceived self-efficacy to predict coping with daily struggles and adaptation after experiencing stressful life events. The GSES with its 10 items and additional context specific questions will allow us to explore the key anticipated mediators of intervention outcome (namely self-efficacy in the context of Covid-19). The context-specific questions will represent items of most importance to this group.

vi. Information on the utility will be gathered using the EQ-5D-5L questionnaire that includes 5 dimensions of health mobility, self-care, usual activities, pain/discomfort and anxiety/depression[28].

vii. Health care resource use to inform the health economics evaluation will be gathered using a client services receipt inventory (CSRI)[29], adapted specifically to capture resource use in patients with long COVID.

viii. The Acceptability of Intervention Measure (AIM) 4 item tool will be used to evaluate the approval, appeal, likability and approachability (welcomeness). The Intervention Appropriateness Measure (IAM) 4 item tool will be used to assess the level of fitting, suitability, likability and match.

ix. The Feasibility of Intervention Measure (FIM) 4 item tool will be used to assess the perception of the service users and providers if the intervention is implementable, possible, doable, and easy to use. All three implementation measures use a 5-point ordinal scale with options ranging from "completely disagree" to "completely agree". Items scores are summed or averaged to provide a single scale score for each implementation construct[30].

5.6 Exploratory Outcome Measure(s)

i. Perceptions of acceptability and feasibility of trial processes (randomisation, outcomes measures etc).
ii. Perceptions of acceptability and usability of codesign resources – book and digital.
iii. Perceptions of training for intervention practitioners.
iv. Perceptions of acceptability and feasibility of one-to-one coaching sessions.
v. Skills required to deliver the intervention and alignment with intervention fidelity.
vi. Indicators of intervention success and the extent to which they match to outcomes of importance to participants with long Covid and those delivering the intervention.
vii. Processes which facilitate and/or act as barriers to implementation, including contextual factors such as organisational, personal or professional issues.
viii. Factors required to enable sustainability and spread to other NHS sites beyond the project timeline.

6 Trial Design and Setting

The trial is a two-arm individually randomised effectiveness trial comparing the 'LISTEN intervention' to Usual Care (UC) for non-hospitalised individuals living with long Covid in the UK. We will recruit 558 individuals with long Covid. Recruitment will be inclusive of age, gender, ethnic and disability groups. All attempts will be made to reflect current data on people experiencing long Covid and include people across age, ethnic groups and those with and without previous long-term conditions and people working in health and social care. Information about the study will be made available to participants through a variety of sources including NHS, third sector settings and community support groups and interested individuals with long Covid will be able to self-refer into the trial. Statistical analysis will be blinded; participants and site staff will be unblinded.

Recruitment is anticipated to last 9 months, with each participant participating in the trial for 3 months and the last follow-up 12 months after start of recruitment. Final study report is anticipated to be submitted in month 24. Initially, sites will be opened in Wales, London, East of England and the Midlands with further sites across England being opened as becomes feasible. All participants will be

required to provide online informed consent for the primary study and will be asked to consider sharing contact details for longer-term follow up after the end of this trial (defined as date of final data capture to meet the trial endpoints).

The LISTEN intervention will be delivered by community rehabilitation teams (intervention practitioners) and will consist of personalised self-management support consisting of one-to-one practitioner delivered self-management support sessions (up to 6 sessions) and access to co-designed resources. Self-management coaching sessions will be delivered remotely, via video call or by telephone according to participant preference.

Outcome data will be collected at baseline and 3 months after randomisation (primary outcome timepoint) for both control and intervention participants. Data collection will be achieved via electronic case report forms, self-reported by participants and accessed using a purpose-developed online database. For participants who have issues accessing the database or have difficulties using a computer, the central CTR team will be available to provide IT support by telephone. If participants do not have access to a computer or are unwilling to use the internet, paper case report forms can be sent by post and the central CTR team will ring the participant to record the answers to the questionnaires.

An internal pilot with progression criteria will assess site opening and recruitment. In order to understand mechanisms of action and facilitate future scale-up, we will conduct a mixed-methods process evaluation to capture in detail implementation aspects of the intervention, including barriers to it, as part of the trial.

6.1 Risk Assessment

A Trial Risk Assessment has been completed to identify the potential hazards associated with the trial and to assess the likelihood of those hazards occurring and resulting in harm. This risk assessment includes:

- The known and potential risks and benefits to human subjects
- How high the risk is compared to normal standard practice
- How the risk will be minimised/managed

This trial has been categorised as a low risk where the level of risk is comparable to the risk of standard medical care. A copy of the trial risk assessment may be requested from the Trial Manager. The trial risk assessment is used to determine the intensity and focus of monitoring activity (see section 25.1).

7 Site and Investigator Selection

This trial will be carried out at participating sites (NHS Trust/Health board) in the UK. Each site may have multiple rehabilitation teams. All sites that are interested in participating in the trial will be required to complete a Site Feasibility Questionnaire to confirm that they have adequate resources and experience to conduct the trial.

Before any site can begin recruitment a Principal Investigator at each site must be identified. The following documents must be in place and copies sent to the LISTEN mailbox (LISTEN@cardiff.ac.uk):

- The approval letter from the site's R&D Department
- Favourable opinion of host organisation/PI from Main Ethics committee
- A signed Trial Agreement (mNCA)
- Current Curriculum Vitae and GCP training certificate of the Principal Investigator (PI)
- Completed Site Staff Delegation Log
- Full contact details for all host care organisation personnel involved, indicating preferred contact
- A copy of the most recent approved version of the Participant Information Sheet(s) and Consent Form(s) on host care organisation headed paper
- A copy of the most recent approved GP letter on host care organisation headed paper

Upon receipt of all the above documents, the Trial Manager will send written confirmation to the Principal Investigator detailing that the site is now ready to recruit participants into the trial. This letter/email must be filed in each site's Investigator Site File.

Occasionally during the trial, amendments may be made to the trial documentation listed above. CTR will issue the site with the latest version of the documents as soon as they become available. It is the responsibility of the CTR to ensure that they obtain local R&D approval for the new documents.

Site initiation will be by attendance at a remote LISTEN launch meeting.

8 Participant Selection

Participants are eligible for the trial if they meet the following inclusion criteria and none of the exclusion criteria apply. They must be an English or Welsh speaker or have access to someone who can act as a translator (Welsh information sheets will be made available and the intervention materials

will be translated into Welsh). All queries about participant eligibility will be managed by the central LISTEN trial team before randomisation/registration.

8.1 Inclusion Criteria

- Age ≥18 years **AND**
- Experience persistent illness (at least one Long Covid symptom for 12 weeks or longer **AND**
- Positive SARS-CoV-2 PCR or antigen test (positive Covid19 test) during the acute phase of illness **OR**
- Positive SARS-CoV-2 antibody test (positive Covid-19 antibody test) at any time point in the absence of SARS-CoV-2 (Covid-19) vaccination history **OR**
- Loss of sense of smell or taste during the acute phase in the absence of any other identified cause **OR**
- Symptoms consistent with SARS-CoV-2 (Covid-19) infection during the acute phase and high prevalence of Covid-19 at time and location of onset **OR**
- At least one symptom consistent with SARS-CoV-2 (Covid-19) infection during the acute phase **AND** close contact of a confirmed case of Covid-19 around the time of onset.
- Must have consulted with their GP to rule out serious complications or the need for further investigation in relation to persistent symptoms following Covid-19 infection.

8.2 Exclusion Criteria

Participants will only be excluded if they are recieving palliative or end of life care, have been hospitalised for treatment of Covid symptoms, during the acute phase of Covid illness , or are currently participating in any Covid intervention trial focussing on improving long Covid symptoms. Individuals who have participated in the LISTEN co-design activities will also not be eligible for the trial.

9 Recruitment, Screening and Registration

9.1 Participant Identification

We will recruit non-hospitalised individuals living with long Covid in the UK. Recruitment will be inclusive of age, gender, ethnic and disability groups through primary care. All attempts will be made to reflect current data on people experiencing long Covid and include people across age, ethnic groups and those with and without previous long-term conditions, people working in health and social care.

Information about the trial will be made available to participants through a variety of sources including NHS, third sector settings and community support groups and interested individuals with long Covid will be able to self-refer into the trial. GP practices and NHS organisations running long Covid clinics will operate as Participant Identification Centres (PICs).

Participants will be recruited by the following methods:

- Mail-out

GP practices will be set up as PICs. Invitation letters from GP practices will be sent out to potentially eligible participants via the docmail system.

Trial sites and PICs able to identify potential participants from database records will direct potential participants to the expression of interest online form and PIS.

- Text messages

Sites will be able to use text messaging systems to send template texts to potential participants identified from database record searches. The text message will introduce the LISTEN study and include a link to the LISTEN website for more information.

Sites will be able to follow up recruitment material with a phone call to potential participants. This will give people the opportunity to ask questions and to be supported to express interest in taking part.

- Routine clinic attendance

Potential participants attending routine clinic appointments at either a trial site or a PIC site during the recruitment phase will be screened for eligibility during routine appointments, those eligible and interested in participating will be provided with a PIS.

- Publicity

Advertisements (e.g. posters, flyers) will be provided to trial sites, PICs and community support groups so they can advertise the trial on their premises and during clinics/support groups. Members of the trial team and/or trial sites may also be invited to support groups to talk about the trial. The trial will also be advertised via social media through written and video content, which will be co-developed with the LISTEN PPI group. It will be explicit in any advertisements that the trial is only available in particular regions.

All outward-facing communications (including audio and filmed materials) about the project will be reviewed by our Inclusion Advisors (Diversity and Ability Social Enterprise) who will review for representativeness, accessibility and inclusivity.

Potential participants approached during a clinic or via the mail-out will have been provided with a participant information sheet. Advertisements will also refer to the LISTEN trial website where all the study information needed in order to make an informed choice about taking part in the trial will be presented.

Potential participants will be asked to self-refer to the trial by completing the expression of interest form and eligibility checklist via the link provided on the LISTEN website. If they are unable or unwilling to use the internet, local advertisements will also contain a central phone number for potential participants to contact the central CTR team for IT support who will then arrange to provide assistance over the telephone.

For potential participants able and willing to use the internet, the expression of interest form will be available to be completed until eligibility has been confirmed for the entire sample size. Prior to the initial target sample size being recruited, information will be provided on the final page of the expression of interest and eligiblity checklist form, thanking them for their interest, explain the next steps and advise that they will be contacted in due course. Once eligibility has been confirmed for the entire sample size and the study is closed to recruitment, the expression of interest page will be disabled; however, interested individuals will be able to provide their contact details to receive trial updates and results.

Those participants who are interested in the trial, are deemed eligible and are able/willing to provide their data online, will be emailed by the LISTEN trial providing a link to complete online consent for study participation. Once they have consented, they will receive further emails from the LISTEN trial providing them with links to completethe baseline measures directly online. Twenty percent of eligible participants will be telephoned by the central CTR team as an additional eligibility check.

As part of the expression of interest, potential participants will be asked to select if they are willing to complete the data collection (questionnaires) online (accessed via links sent within emails from the LISTEN trial) or whether would require their data to be collected over the telephone.

The baseline and follow-up case report forms can be sent in the post if preferred. A member of the central trial team will then telephone the participant at a time agreeable to them to complete the baseline measures with the participant over the telephone, entering the data into the LISTEN database on the participants' behalf.

Potential participants unable and/or unwilling to use the internet and who thus cannot complete their own online expression of interest once assistance has been provided, will have their contact details taken. The online expression of interest form and initial eligibility review will be completed on the potential participants behalf. The central CTR team or relevant site will make provisions to complete the online expression of interest form, undertake the eligibility assessment and provide either verbal consent over the telephone or a face to face consent option (see section 9.4).

All potential participants who express interest in the study will have their name, age, contact details, GP surgery address and eligibility assessment collected during the expression of interest process.

These details will be stored securely on Cardiff University servers. How their data will be managed and secured is detailed in the data management section 16.

9.2 Screening Logs

A screening log will be generated centrally by the LISTEN database.

9.3 Recruitment Rates

The overall recruitment target is 558 (across 24 sites). This equates to recruiting 24 participants per site over 9 months and a monthly recruitment target of 2.6 participants per site.

9.4 Informed Consent

The provision of information and the opportunity to ask questions will be as described in section 9.1.

Electronic consent: Participants who are able and willing to use the internet will be required to provide electronic informed consent for the LISTEN trial by completing theonline Consent Form accessed via a link sent in an email to eligible participants from the LISTEN trial.

The e-consent form will consist of declarations with yes/no tick boxes, typed name, typed date and date of birth, and an automatic date/time stamp generated as part of the audit trail upon saving the form.

Participants and the relevant site will have the ability to download the completed consent form from the website/trial database. An email will also be sent to the participant and the site confirming the participant's consent. The email will only contain a participant's initials and date of birth. Sites should save a copy of the email in their investigator site file. The participant's GP should be informed of their participation after randomisation.

- **Participants unable and/or unwilling to use the internet** will be able to provide written informed consent face-face in the presence of site staff or if this is not possible, verbal consent will be taken by the central CTR team or site staff over the telephone.
- **Written Consent provided face-face in the presence of site staff.** The potential participant will be provided with a paper copy of the PIS and a paper copy of the consent form and will provide written informed consent in the presence of site personnel delegated to do so.
- **Verbal Consent provided over the telephone.** To ensure the trial is inclusive and has reach, where online consent or face-face consent is not possible, verbal consent will be sought by the central CTR team or site personnel delegated to do so over the telephone. A version-controlled script will be used and the consent conversation will be fully documented on the LISTEN verbal consent form by the site staff undertaking the phone call. Where possible, the

potential participant will be provided with a copy of the PIS and a copy of the completed verbal consent form.

In both situations where the site takes Consent (written or verbal), the site will confirm eligibility, obtain GP contact details and provide the central CTR team with a copy of the completed consent form (if collected face to face) and it will be recorded on the database that consent and eligibility assessment were taken at site. An email will be sent to the site confirming that the participant's consent has been recorded. The email will only contain a participant's initials and date of birth. Sites should save a copy of the email and consent form (if collected face to face) in their investigator site file.

All participants will also be asked to consider sharing contact details for longer term follow up after the end of this study. Given this study is via self-referral, participants will have as long as they require before providing informed consent. Please note, only when informed consent has been obtained from the participant and they have been randomised into the trial can they be considered a trial participant.

The right of the participant to refuse to participate in the trial without giving reasons will be respected. The participant will be free to withdraw at any time from the protocol treatment without giving reasons and without prejudicing their further treatment. The participant's GP will be informed of their participation.

9.5 Registration and Randomisation

9.5.1 Registration

Registration will consist of a self-registration process carried out via the online LISTEN platform, or by contacting the central CTR team by telephone, or by the site completing an expression of interest form (see section 9.1).

9.5.2 Randomisation

Participants will be individually allocated to the intervention or usual care arm using simple randomisation stratified by site. This will be implemented via RedCap once the baseline assessments have been completed. Once randomised, site staff from their local site will be alerted to a newly recruited participant and those in the intervention arm will arrange the first online coaching session. The randomisation process is described in a separate randomisation plan.

10 Withdrawal & Lost to Follow-up

10.1 Withdrawal

Participants have the right to withdraw consent for participation in any aspect of the trial at any time. The participants care will not be affected at any time by declining to participate or withdrawing from the trial. If a participant initially consents but subsequently withdraws from the study, clear distinction must be made regarding what aspect of the study the participant is withdrawing from. These aspects could be:

- Withdrawal from intervention
- Partial withdrawal from further data collection
- Complete withdrawal from further data collection
- Withdrawal of permission to use data already collected

The withdrawal of participant consent shall not affect the trial activities already carried out and the use of data collected prior to participant withdrawal. The use of the data collected prior to the withdrawal of consent is based on informed consent before its withdrawal.

Furthermore, it is important to collect safety data ongoing at the time of withdrawal, especially if the participant withdraws because of a safety event. There is specific guidance on this contained in the Participant Information Sheet but briefly:

A participant may withdraw or be withdrawn from the trial intervention for the following reasons:

- Non-compliance
- Intolerance to intervention

In all instances, participants who consent and subsequently withdraw should complete a withdrawal form which will be sent to them via a link within an email following consent The withdrawal form can also be completed on the LISTEN database on the participant's behalf by the central trial team or site researcher/clinician based on information provided by the participant . Any queries relating to potential withdrawal of a participant should be forwarded to the trial email address.

10.2 Lost to Follow up

We will make every effort to reduce loss to follow-up using the methods listed below:

i. We will emphasise the importance of getting follow-up data to all participants at baseline and the follow-up assessment.

ii. Participants will have two weeks prior and two weeks after follow-up measure time points to complete them. Automated reminders will be sent 2 weeks ahead of the assessment due date. If the assessments are not completed within 2 weeks after the due date, the trial team will telephone the participant to prompt outcome measure completion.

iii. We will invite a selection of participants (from both those who receive the LISTEN intervention and those who receive usual care) to interview (process evaluation) at the initial registration and gather information on the most suitable day and time for any follow-up interviews.

iv. For the interviews, up to five attempts will be made to contact a participant to arrange a date for their interview.

11 Trial Intervention

11.1 The 'LISTEN' Personalised Self-Management Support Intervention

The trial intervention will involve remotely delivered (via a secure web video conferencing system or telephone), one to one personalised self-management support session (up to six sessions over 10 weeks, each maximum of one hour), incorporating digital and paper-based self-management resources accessible and applicable to this population. These will include narratives of individuals with long Covid, and their problem-solving ideas and strategies. Access to peer-support groups and specialist advice will also be promoted. The key sources of self-efficacy, goal mastery and modeling are integral to the intervention, as both mediators of change and an anticipated outcome.

Participants allocated to the intervention arm will receive coaching sessions from NHS practitioners trained to support people with long Covid with the confidence, skills and knowledge to self-manage everyday life. Practitioners will be trained to use language that focuses on exploring participants' assets as well as problems and work towards outcomes of importance shaped by their needs and priorities. In partnership, they will develop strategies and knowledge to aid recovery and work towards meaningful goals. Participants will also gain access to the new codesign resources in the form of a book and web based resources.

11.2 Comparator

All participants randomised to control will receive usual care as currently available in the NHS, within the participants' region. The current standard care pathway is variable across the UK, ranging from access to long Covid specialist clinics to access use of the My Covid Recovery App. The LISTEN team will signpost them to information about local services as required. We will assess usual care within the trial regions during site set up and during the process evaluation interviews with site clinical

practitioners after the clinical trial has been completed. Given the potential for contamination, we will also gather detailed records of usual care (through the use of a health services resource questionnaire) in those randomised to the comparator arm as part of our process evaluation.

12 Trial Procedures

12.1 Internal Pilot

An internal pilot will assess site opening and recruitment. The overall recruitment target is 558 (across 24 sites) to be recruited over 9 months. This equates to recruiting 24 participants per site over 9 months and a monthly recruitment target of 2.6 participants per site which should be stable in all sites 5 months after recruitment activated. The internal pilot end date is month 5 of recruitment. The traffic light system (green, amber, red) of progression criteria as proposed by Avery et al[31] will guide decision making with green resulting in the trial continuing as planned; amber, the trial continuing with changes; red: the trial stops.

Table 1. Internal Pilot Progression Criteria

Progression Criteria (assessed at month 5 of recruitment)	Go (green)	Amend (amber)	Stop (red)
Sites open	All 14-18 sites	10-13 sites	9 sites or fewer
Average recruitment rate per month per open site	5 or more participants	2-4 participants	Fewer than 2participant

Data completeness at follow-up and intervention adherence and fidelity will not be formal progression criteria but will be monitored with reporting to the trial steering committee.

12.2 Staff Training and Assessment of Intervention Fidelity of Delivery

Participating practitioners within community teams will receive training co-delivered by Bridges Self-Management (Bridges) and individuals with long Covid. Practitioners will access their training resources via an on-line platform and will receive additional supervision from the Bridges team and a Clinical Psychologist.

In order to confirm intervention fidelity, we will carry out an independent analysis of 10% of one-to-one remote self-management coaching sessions delivered in intervention sites, recorded via Zoom or MS Teams depending on participants' preference. These will be reviewed against pre-defined fidelity markers. We will capture reflections from the training delivery team about methods used to engage and sustain fidelity of intervention delivery, through the completion of online reflective journals.

12.3 Assessments

12.3.1 Baseline

Participants who have consented to take part in the trial, will be sent a link via email to the baseline questionnaires which will be self-completed online (via electronic case report forms (see section 9.1 for more detail on how the forms can be completed).

12.3.2 Follow-up

Follow up questionnaires at 6 weeks and 3 months (see Table 1) after randomisation (primary outcome timepoint) for both control and intervention participants will be collected using the same approach.

Table 2. Schedule of Events[1]

	VISITS						
**Procedures**	**Screening**	**Consent**	**Baseline**	**Intervention (up to 10 weeks)**	**6 week (interim data collection point)**	**3 month Follow Up**	**Process Evaluation**
**Screening (sites/PICs)**	X						
**Information provision (mail-out, in clinic, publicity)**	X						
**Self-referral expression of interest**	X						
**Self-assessment online eligibility check[A]**		X					

[1] Taken from the HRA CTIMP protocol template (2016).

Procedures	VISITS						
	Screening	Consent	Baseline	Intervention (up to 10 weeks)	6 week (interim data collection point)	3 month Follow Up	Process Evaluation
**Informed consent**		X					
**Demographics[B]**			X				
**Long Covid history[B]**			X				
**Ox-PAQ Questionnaire[B]**			X			X	
**SF-12 Questionnaire[B]**			X			X	
**FIS Questionnaire[B]**			X			X	
**EQ-5D-5L Questionnaire[B]**			X		X	X	
**GSES Questionnaire[B]**			X			X	
**Health service use questionnaire[B,C]**			X		X	X	
**Randomisation**			X				
**Delivery of intervention**				X			
**AIM[D]**							X
**IAM[D]**							X
**FIM[D]**							X
**Semi-structured Interviews[D, E]**							X
**SAE Reporting**				X		X	

[A:] A random selection of participants will be phoned by the central CTR team for an eligibility review
[B] If participants are unable/unwilling to complete the questionnaires online, a paper/ hard copy can be sent in the post and the forms completed over the telephone with a member of the central CTR team.
[C] A diary will be available to download to help participants record their appointments to complete the health service use questionnaire.
[D] Process evaluation questionnaires and interviews will be conducted with a subset of participants and will include both those who received the LISTEN intervention and usual care.

[E] *Process evaluation interviews and focus groups will also be conducted with a subset of staff members involved in LISTEN delivery*

12.3.3 Mixed Methods Process Evaluation

We will carry out a theory-driven, detailed process evaluation within the trial. We will use validated implementation scales, qualitative interviews in a sub-set of participants who have received either the LISTEN intervention or usual care and focus groups with practitioners to explore issues relating to the context, mechanisms and outcomes of the intervention and how they may differ to usual care.

We have designed the process evaluation in accordance with the MRC framework[32] for evaluation of complex interventions, as LISTEN fits the criteria for a complex intervention with, multiple interacting components. To further guide the collection of implementation data we will apply the Proctor implementation outcome taxonomy[33], which offers the current gold standard in conceptually articulating different aspects of implementation to be assessed. Of this taxonomy, we will assess the acceptability, feasibility and appropriateness of LISTEN as perceived by the service users who have completed the LISTEN intervention (at 3 months after randomisation) and providers (at 3- and 6-months post first randomisation within their services) thus facilitating the assessment of early and more established implementation. The brief validated scales (4 items each; 12 implementation items in total; all scored on 5-point Likert scales) that will be used include the Acceptability of Intervention Measure (AIM), the Intervention Appropriateness Measure (IAM), and Feasibility of Intervention Measure (FIM)[30].We will ask all participants who complete the LISTEN intervention (up to maximum of n=234) to complete the implementation measures and will target all providers (census approach) involved in delivery of LISTEN. We will conduct semi-structured interviews with a sub-set of participants (up to n=60). All interviews will be carried out remotely after the primary outcome data collection time point. Interviews with participants who received the LISTEN intervention will explore experiences of engaging with LISTEN, its perceived acceptability, feasibility and appropriateness for them as an intervention to address their holistic needs. We will also ask about their experiences of interfacing with the rehabilitation services through which LISTEN will be offered, challenges they faced in accessing or receiving LISTEN and how they overcame such challenges and completed LISTEN. Interviews with those who received usual will focus on the content of the usual care intervention and the experiences of those who have received usual care. We will sample purposively with the aim of ensuring a sample that reflects the current data on who is more likely to experience long Covid. e.g women, between ages 20-60, and those living with other long-term conditions.

We expect that with this sample we will achieve thematic saturation in what participants report in relation to LISTEN delivery. We intend for these interviews in this sample to be used to supplement

the knowledge gathered from other aspects of the process evaluation in terms of usual care and its variability across the UK. The topic guide for the interviews will be designed jointly with our PPI panel and people living with and recovered from long Covid who have already informed our study design stage, prior to funding been made available.

We will also conduct up to six focus groups with an average of n=6 participants in each group, providing representation from across all recruiting rehabilitation practitioners we anticipate that with this sample size we will be able to reach saturation in the thematic areas relating to LISTEN delivery from the perspective of the providers. Focus groups will be used to explore (i) how training was delivered to staff and its acceptability to them, (ii) how self-management support was administered and whether staff perceived that they were able to deliver the intervention as intended and any modifications required and (iii) any contextual factors within community teams and the wider health service environment that affected intervention implementation. This will increase understanding of barriers and facilitators and generate insights into how this might enable or prevent sustainability and spread thereafter, should the intervention be shown to be effective and cost-effective.

13 Safety Reporting

For the purpose of this trial, only adverse events (AEs) relating to psychological distress and serious adverse events (SAEs) will be reported and we will not collect, or report expected events related to Long Covid symptoms. The Principal Investigator is responsible for ensuring that all site staff involved in this trial are familiar with the content of this section.

All SAEs must be reported immediately (and within 24 hours of knowledge of the event) by the PI at the participating site to the Trial team unless the SAE is specified as not requiring immediate reporting (see section 13.2).

13.1 Definitions

**Term**	**Definition**
**Adverse Event (AE)**	Any untoward medical occurrence in a participant or clinical trial participant administered an intervention which are not necessarily caused by or related to that product
**Serious Adverse Event (SAE)**	Any adverse event that -<br>• Results in death<br>• Is life-threatening*

	• Required hospitalisation or prolongation of existing hospitalisation**<br>• Results in persistent or significant disability or incapacity***<br>• Consists of a congenital anomaly or birth defect<br>• Other medically important conditions***

***Note:** The term 'life-threatening' in the definition of serious refers to an event in which the trial participant was at risk of death at the time of the event or it is suspected that used or continued use of the product would result in the subjects death; it does not refer to an event which hypothetically might have caused death if it were more severe.

** **Note:** Hospitalisation is defined as an inpatient admission, regardless of the length of stay, even if the hospitalisation is a precautionary measure for continued observation. Pre-planned hospitalisation e.g. for pre-existing conditions which have not worsened, or elective procedures, does not constitute an SAE.

*** **Note:** other events that may not result in death, are not life-threatening, or do not require hospitalisation, may be considered as an SAE when, based upon appropriate medical judgement, the event may jeopardise the participant and may require medical or surgical intervention to prevent one of the outcomes listed above.

13.2 Trial Specific AE Reporting Requirements

For the purpose of this trial, only the following events will be considered as AEs:

- Psychological distress or/and new/progressed psychiatric conditions will be classified as an AE only where it does not meet the definition of an SAE (i.e. a deterioration in mental health associated with an imminent risk of death would be an SAE).

Adverse events can be reported by the clinical practitioners on the AE Reporting form in the LISTEN database. The central CTR team will be notified of the uploaded AE and report this to the site PI. If the participant is in the intervention group, the trial clinical psychologist will also be informed.

13.3 Trial Specific SAE Reporting Requirements

This trial is not a Covid symptom trial, we will gather long Covid history at baseline. We are not monitoring pregnancy outcomes. For the purpose of this trial, we will report, within 24 hours of knowledge of the event, all other SAEs that meet the definition in Section 13.1.

13.4 SAE Causality

A causal relationship will be assessed for the intervention.

The Principal Investigator (or another delegated suitably qualified clinician or intervention practitioner from the study team registered on the delegation log) will assess each SAE to determine the causal relationship and the Chief Investigator (or another appropriately qualified member of the Trial Management Group) can also provide this assessment where necessary:

Relationship	Description	Is there a reasonable possibility that the SAE may have been caused by the intervention?
**Unrelated**	There is no evidence of any causal relationship with the intervention	No
**Unlikely**	There is little evidence to suggest there is a causal relationship with the intervention (e.g. the event did not occur within a reasonable time after administration of the trial medication). There is another reasonable explanation for the event (e.g. the participant's clinical condition, other concomitant treatment).	No
**Possible**	There is some evidence to suggest a causal relationship with the intervention (e.g. because the event occurs within a reasonable time after administration of the trial medication). However, the influence of other factors may have contributed to the event (e.g. the participant's clinical condition, other concomitant treatments).	Yes
**Probable**	There is evidence to suggest a causal relationship and the influence of other factors is unlikely.	Yes
**Definite**	There is clear evidence to suggest a causal relationship and other possible contributing factors can be ruled out.	Yes

The causality assessment given by the Principal Investigator (or delegate) cannot be downgraded by the Chief Investigator (or delegate), and in the case of disagreement, both opinions will be provided.

13.5 SAE Expectedness

For the LISTEN trial there will be no expected SAEs. SAEs which add significant information on the specificity or severity of a known, already documented adverse event constitute unexpected events. For example, an event more specific or more severe than that described in the protocol is considered unexpected.

13.6 SAE Reporting Procedures

SAEs can be reported by the participant or the site team responsible for the participant. The participant can notify the trial team and site of an SAE occurring by completing the online "Report a Problem" form. This form will ask them for information regarding the event including, the onset of the event, detail of the event and treatment received.

13.6.1 SAE Participating Site Responsibilities

For SAEs reported by the participant, the PI (or delegated appropriately qualified clinician or practitioner from the study team registered on the delegation log) should review the submitted Report a Problem form, and complete a corresponding SAE form on the LISTEN database. The site staff member should electronically sign and date the SAE Form to acknowledge that they have performed the seriousness and causality assessments. Investigators should also report SAEs to their own health boards or trust in accordance with local practice.

A completed SAE form for all events requiring immediate reporting should be submitted within 24 hours of knowledge of the event. A separate form must be used to report each event, irrespective of whether or not the events had the same date of onset.

The participant will be identified only by trial number, partial date of birth (mm/yy) and initials. The participant's name should not be used in any correspondence with the CTR team.

It is also required that sites respond to and clarify any queries raised on any reported SAEs and report any additional information as and when it becomes available through to the resolution of the event. Additionally, the CTR may request additional information relating to any SAEs and the site should provide as much information as is available to them in order to resolve these queries.

Serious adverse events should be reported from the time of signature of informed consent, throughout the treatment period up until the 3-month follow-up data has been collected.

An SAE form is not considered as complete unless the following details are provided:

- Full participant trial number

- A Serious Adverse Event
- A completed assessment of the seriousness, and causality as performed by the PI (or another appropriate clinician or practitioner from the study team registered on the delegation log).

If any of these details are missing, the site will be contacted and the information must be provided by the site to the CTR within 24 hours.

13.6.2 The CTR Responsibilities

Following the initial report, all SAEs should be followed up to resolution wherever possible, and further information may be requested by the CTR. Follow-up information must be provided when available.

Once an SAE is received at the CTR, it will be evaluated by staff at the CTR and sent to the Chief Investigator/s (or their delegate) for an assessment of expectedness.

For all non-CTIMP studies only reports of SAEs that are:

- **related** to the study (i.e. they resulted from the administration of any of the research procedures) and
- **unexpected** (i.e. not listed in the protocol as an expected occurrence)

should be submitted to the REC. These should be sent within 15 days of the chief investigator/s becoming aware of the event. There is no requirement for annual safety reports in addition to the information provided through the annual progress report.

13.7 Contraception and Pregnancy

There is no requirement for Women of Child Bearing Potential (WOCBP) entering into this trial to take contraception during the trial as a condition of participation. Sites do not need to report any pregnancy occurring during the course of the intervention as there is no risk the intervention will have an effect on the pregnancy or baby.

13.8 Urgent Safety Measures (USMs)

An urgent safety measure is an action that the Sponsor or Chief Investigator/s may carry out in order to protect the subjects of a trial against any immediate hazard to their health or safety. It is extremely unlikely that any urgent safety measures should be required for this trial, but any urgent safety measure relating to this trial that does occur must be notified to the local Institutional Review Board (IRB) immediately by telephone, and in any event within 3 days in writing, that such a measure has been taken. USMs reported to the CTR will be handled according to CTR processes.

14 Statistical Considerations

14.1 Randomisation

Participants will be individually allocated to the intervention or usual care arm using simple randomisation stratified by site. This will be implemented via RedCap.

14.2 Blinding

It is not possible for participants to be blinded to allocation to intervention or usual care. All data collection (outcome assessment) is self-reported and submitted via online system. In cases where telephone completion of outcome assessments is requested, this will be done by the central CTR team. All statistical analysis will be carried out blind to allocated treatment. Treatment arm will be requested following completion of this and testing of analysis syntax (using dummy randomisation data).

14.3 Sample Size

We aim to detect an MCID effect size of 0.32 between randomised arms in the primary outcome of the routine activities domain of the Ox-PAQ with 90% power whilst controlling the two-sided type I error level at 5%.[24] A conventional individually randomised trial would require 414 participants (based on a two-sample t-test), but since the intervention will be delivered by 24 community rehabilitation teams, we must also take potential clustering in the intervention arm into account. Assuming an intraclass correlation (ICC) of 0.03 in the intervention arm, 24 clusters with 10 participants each in the intervention arm and 234 participants in the usual care arm (i.e. a total of 474 participants) are required for 90% power. This was calculated using the method of Moerbeek and Wong[34] as implemented in v0.7.0 of the R package 'clusterPower'[35]. Assuming 15% loss to follow-up, the overall recruitment target is 558.

14.4 Missing, Unused & Spurious Data

Sensitivity analyses will involve the imputation of missing data as required. Details will be provided in the Statistical Analysis Plan (SAP).

14.5 Procedures for Reporting Deviation(s) from the Original SAP

These will be submitted as substantial amendments where applicable and recorded in subsequent versions of the protocol and SAP.

14.6 Termination of the Trial

Progression criteria for the internal pilot phase are described in section 12.1.

14.7 Inclusion in Analysis

All randomised participants will be included in the analysis dataset.

15 Analysis

15.1 Main Analysis

Participant characteristics will be summarised descriptively by allocation (usual care or intervention). The primary analysis will be intention-to-treat (i.e. participants will be analysed as receiving usual care or intervention according to the randomisation, regardless of adherence to the intervention) and use a partially clustered multi-level model i.e. a linear mixed-effects model with random cluster effects in the intervention arm only and allowing for heteroskedastic individual-level errors[36]. Fixed intervention effects will be included to estimate the difference in average Ox-PAQ routine activities scores at 3 months (adjusted for baseline) between participants receiving the LISTEN intervention and those receiving usual care. The intervention effect will be presented as a point estimate with a two-sided 95% confidence interval and p-value. If the estimate favours the LISTEN intervention and the 95% CI excludes zero, effectiveness of the intervention will be concluded. Similar analyses will be performed for the secondary outcomes.

15.1.1 Sub-Group & Interim Analysis

In secondary analyses, we will add the validated implementation scales as covariates into the model, to assess the impact of implementation perceptions on the outcome measure. We will also adjust for additional covariates such as age, gender, and the index of multiple deprivations. A detailed analysis plan will be finalised prior to the analysis.

15.2 Qualitative Analysis

All data will be entered and stored on NVIVO to enable the initial coding and categorisation of raw data. Descriptive themes will be developed to identify emerging concepts and analysis. Further to the data-driven themes that emerge from the interviews with patients and focus groups with providers, the Consolidated Framework for Implementation Research (CFIR) will be applied to allow us to synthesise findings from an implementation perspective. CFIR is one of the best-established implementation frameworks[37] and aimed as an aid to understand factors that impact upon successful implementation, and then address them. In the context of the study, CFIR suggests 5 major determinants of the implementation of LISTEN:

- LISTEN itself as an intervention, including its theoretical underpinnings
- The implementation process
- The people involved in designing and implementing LISTEN
- The local context in the community services within the trial (including defining usual care across

trial regions)

- The wider context of the NHS and the ongoing pandemic

This analysis will allow us to map barriers/drivers of implementation from the perspective of the service users and providers and map them onto potential implementation support strategies as they have emerged in the CFIR evidence base[38] – in addition to what such strategies might emerge from the process evaluation itself.

The process evaluation will be carried out prior to knowledge of the final statistical analysis of primary and secondary outcomes in order to interpret findings without being influenced by knowledge of the results. The proposed methods will enable triangulation of multiple data sources and theory, and provide an in-depth understanding of the functioning of the intervention, mechanisms and contextual factors and the implementation process to support LISTEN beyond this study. A revised logic model for LISTEN will be produced upon completion of the process evaluation to help support subsequent scale-up.

15.3 Cost-Effectiveness Analysis

The base case analysis will take an NHS and Personal Social Services perspective. In addition, we will record patient expenses and loss of productivity to gauge the impact of the intervention on the burden to the patient and society. We will investigate the implementation cost of the intervention (including training, staff, costs of online resources, e.g., hosting and access,) compared to usual care through review of study notes and discussions with the study team. Furthermore, we will collect patient healthcare resource use using a CSRI, specifically adapted to individuals with long Covid as part of the proposed study. Health care resource use will be collected in the three months before baseline (through patient recall with the option to provide longer-term resource use if patients wish to do so) and at the 3-month follow-up point for both control and intervention groups to estimate the impact of the intervention on use of healthcare resources in primary, secondary and social care as well as patient out-of-pocket expenses and ability to undertake paid work. A cost-utility analysis will be undertaken commensurate with the statistical analysis (regarding primary analysis population, handling of missing data and model used) and will calculate the cost per quality-adjusted life-year gained (based on EQ-5D-5L[1] responses at baseline and 3-months follow-up). A cost-consequences analysis will be conducted, and net monetary benefit calculated to weigh up all costs and outcomes of the intervention. Sensitivity and scenario analyses will explore the impact of uncertainty on the results.

16 Data Management

Source Data is defined as *"All information in original records and certified copies of original records of clinical findings, observations or other activities in a clinical trial necessary for the reconstruction and evaluation of the trial. Source data are contained in source documents."* There is only one set of source data at any time for any data element, as defined in the site source data agreement.

Trial Data	SOURCE DATA								
	CRF/Questionnaire on LISTEN Jisc Online Survey	CRF/Questionnaires on LISTEN database	Electronic or telephone Consent form on LISTEN	Paper consent form for face to face consenting	Audio/video recording[A]	Report a problem on Jisc Online Survey	AE or SAE form on LISTEN database	Withdrawal form on Jisc Online Survey[B] database	Withdrawal form on LISTEN database[B]
Expression of Interest	X[B]	X[B]							
Eligibility	X[B]	X[B]							
Informed Consent			X	X					
Case Report Forms		X			X[A]				
Clinical practitioner session notes		X							
Qualitative interviews and focus groups					X				
Intervention Fidelity					X				
Serious adverse event initially reported by a patient						X			
Serious Adverse Event initially reported by a site							X		

Trial Data	SOURCE DATA									
	CRF/Questionnaire on LISTEN Jisc Online Survey	CRF/Questionnaires on LISTEN database	Electronic or telephone Consent form on LISTEN	Paper consent form for face to face consenting	Audio/video recording[A]	Report a problem on Jisc Online Survey	AE or SAE form on LISTEN database	Withdrawal form on Jisc Online Survey[B]database	Withdrawal form on LISTEN database[B]
Participant withdrawal								X[B]	X[B]

[A] An audio recording will be source data where the telephone or conversation was audio recorded prior to a CRF or transcript being completed/available.

[B] Expression of Interest, eligibility and withdrawal form can be completed by the participant on Jisc Online Surveys or on their behalf by the CTR team or site staff on the LISTEN database

16.1 Data Collection

All data collection for this study will be completed using an online electronic system using electronic CRFs via individual log-ins, either by the participant or the CTR or site team on behalf of the participant. A full data management plan will accompany this protocol and will be stored in the TMF.

16.2 Completion of CRFs

16.2.1 Paper CRFs

Paper CRFs can be sent in the post for participant unable/unwilling to complete the baseline and follow up questionnaires via the internet. The paper CRFs will only be used as a reference document for participants to answer the questions over the telephone with the CTR team member. Paper CRFs will not be accepted for completion.

16.2.2 Electronic CRFs

For initial self-referral expression of interest and eligibility checks, a publicly available survey has been developed on Jisc Online Surveys tool. This same tool will be used by participants if they wish to withdraw from the trial (see section 10) or they wish to report a problem (see section 13). All data entered by participants is secure and confidential.

It is intended that data collection and data management will be developed using REDCap electronic data capture tools. REDCap (Research Electronic Data Capture) is a secure, web-based software platform designed to support data capture for research studies, providing 1) an intuitive interface for validated data capture; 2) audit trails for tracking data manipulation and export procedures; 3) automated export procedures for seamless data downloads to common statistical packages; and 4) procedures for data integration and interoperability with external sources.

A user password will be supplied to investigators upon completion of all processes required prior to opening. Detail can be found in the Data Management Plan, upon request.

17 Translational Research or Sub-Trial

Not applicable.

18 Protocol/GCP Non-Compliance

The Principal Investigator should report any non-compliance to the trial protocol or the conditions and principles of Good Clinical Practice to the CTR in writing as soon as they become aware of it.

19 End of Trial Definition

This trial will have a 3- month follow-up period. Consent for long-term follow-up will also be sought.

The end of the trial is defined as the date of final data capture to meet the trial endpoints. In this case end of the trial is defined as the date on which data for all participants is frozen after the last participant has had their 3-month follow-up and once the mixed-method process evaluation has been completed. Any long-term follow-up will continue after this trial is regarded as completed.

The sponsor must notify the main REC of the end of a clinical trial within 90 days of its completion or within 15 days if the trial is terminated early.

20 Archiving

The TMF and TSF containing essential documents will be archived at an approved external storage facility for a minimum of 10 years. The CTR will archive the TMF and TSFs on behalf of the Sponsor. The Principal Investigator is responsible for archival of the ISF at the site on approval from Sponsor. Essential documents pertaining to the trial shall not be destroyed without permission from the Sponsor. Where there is no reason that data cannot be shared, they should be responsibly shared and made available for re-use via the research data repository record.

21 Regulatory Considerations

21.1 Ethical and Governance Approval

This protocol has approval from a Research Ethics Committee (REC) that is legally "recognised" by the United Kingdom Ethics Committee Authority for review and approval.

This trial protocol will be submitted through the relevant permission system for global governance review dependant on the location of the lead site e.g. DSCHR PCU if Wales led and HRA if England.

Approval will be obtained from the host care organisation who will consider local governance requirements and site feasibility. The Research Governance approval of the host care organisation must be obtained before the recruitment of participants within that host care organisation.

21.2 Data Protection

The CTR will act to preserve participant confidentiality and will not disclose or reproduce any information by which participants could be identified, except where specific consent is obtained. Data will be stored in a secure manner and will be registered in accordance with the General Data Protection Regulation 2018 and the Data Protection Act 2018. The data custodian for this trial is the Kingston University with responsibility for trial data management delegated to Cardiff University.

21.3 Indemnity

- Non-negligent harm: This trial is an academic, investigator-led and designed trial, coordinated by the CTR. The Chief Investigator, local Investigators and coordinating centre do not hold insurance against claims for compensation for injury caused by participation in a clinical trial and they cannot offer any indemnity. The Association of the British Pharmaceutical Industry (ABPI) guidelines will not apply.

- Negligent harm: Where studies are carried out in a hospital/service, the hospital/service continues to have a duty of care to a participant being treated within the hospital/service, whether or not the participant is participating in this trial. Kingston University does not accept liability for any breach in the other hospital's/service's duty of care, or any negligence on the part of employees of hospitals/services. This applies whether the hospital/service is an NHS Trust or not. The Sponsor shall indemnify the site against claims arising from the negligent acts and/or omissions of the Sponsor or its employees in connection with the Clinical Trial (including the design of the Protocol to the extent that the Protocol was designed solely by the Sponsor and the Site has adhered to the approved version of the Protocol) save to the extent that any such claim is the result of negligence on the part of the Site or its employees.

All participants will be recruited at NHS sites and therefore the NHS indemnity scheme/NHS professional indemnity will apply with respect to claims arising from harm to participants at site management organisations.

21.4 Trial Sponsorship

Kingston University will act as Sponsor for trial. The Sponsor has/will be delegating certain responsibilities to Cardiff University (CTR), the Chief Investigators, Principal Investigators, host sites and other stakeholder organisations as appropriate in accordance with the relevant agreement that is informed by regulation and trial type.

21.5 Funding

This study has been funded by the National Institute of Health Research.

22 Trial Management

22.1 TMG (Trial Management Group)

A trial management group (TMG), including all co-applicants, will meet monthly to discuss key management issues and where milestones will be monitored. TMG members will be required to sign up for the remit and conditions set out in the TMG Charter. All activities within this trial will adhere to the UKCRC registered Cardiff University Centre for Trials Research Standard Operating Procedures (SOPs), including those for data management and protection, serious adverse event reporting, maintaining trial documentation according to GCP and archiving data. Study-specific SOPs will be developed.

22.2 TSC (Trial Steering Committee)

A Trial Steering Committee (TSC) will be established and will meet 4 times over 24 months. It will comprise of an independent Chair with expertise in trials of self-management support, an independent rehabilitation expert, an independent Statistician, a Health Economist, representatives from the PPI panel with the CI, Statistician and Senior Trial Manager as observers. The TSC will determine at their first meeting whether a separate Data Monitoring Committee is required. The TSC will provide overall supervision for the trial. TSC members will be required to sign up for the remit and conditions set out in the TSC Charter. The TSC will fulfull the function of the Data Monitoring Committee.

23 Quality Control and Assurance

23.1 Monitoring

The clinical trial risk assessment has been used to determine the intensity and focus of central and on-site monitoring activity in the LISTEN trial. Low monitoring levels will be employed and are fully documented in the trial monitoring plan.

Investigators should agree to allow trial-related monitoring, including audits and regulatory inspections, by providing direct access to source data/documents as required. Participant consent for this will be obtained.

Findings generated from on-site and central monitoring will be shared with the Sponsor, CI, PI & local R&D.

23.2 Audits & inspections

The trial is a participant to inspection by the NIHR as the funding body. The trial may also be a participant to inspection and audit by Kingston University under their remit as Sponsor.

24 Publication policy

All publications and presentations relating to the trial will be authorised by the Trial Management Group. We will actively collaborate and engage with our stakeholders (Government, Department of Health, NHS bodies) and long Covid advocacy groups, to increase their investment, generate ownership and build trust. As part of our Pathway to Impact, we will produce the LISTEN manual, including a clinical delivery and implementation guide (for services). We will provide service user-facing materials (including podcasts and blog posts from individuals with long Covid), a LISTEN briefing document and an infographic for DHSC/SAGE, namely a two-page summary with what we found, what it means and how to scale it. In the last 3 months, we will hold a knowledge mobilisation event to discuss and debate the findings and consider implementation at scale. Publications (for example scientific journal articles) that rely on research findings must include a statement with information about the research data and where and under what conditions they may be accessed. This should reflect funder or publishers' rules, where appropriate.

25 Milestones

Milestones specific to this trial can be found in the Trial Gantt, which can be obtained from the Trial Manager.

26 References

1. Callard, F. & Perego, E. How and why patients made Long Covid. Soc Sci Med 268, 113426 (2021).

2. Davis, H. E. et al. Characterizing Long COVID in an International Cohort: 7 Months of Symptoms and Their Impact. medRxiv 2020.12.24.20248802 (2020) doi:10.1101/2020.12.24.20248802.

3. Ladds, E. et al. Persistent symptoms after Covid-19: qualitative study of 114 “long Covid” patients and draft quality principles for services. BMC Health Serv Res 20, (2020).

4. Living with Covid19 – Second review. NIHR Evidence https://evidence.nihr.ac.uk/themedreview/living-with-covid19-second-review/ doi:10.3310/themedreview_45225.

5. Dickson, A., Knussen, C. & Flowers, P. Stigma and the delegitimation experience: An interpretative phenomenological analysis of people living with chronic fatigue syndrome. Psychology & Health 22, 851–867 (2007).

6. Patterson, C. Long COVID – we’ve been here before. The British Medical Association is the trade union and professional body for doctors in the UK. https://www.bma.org.uk/news-and-opinion/long-covid-we-ve-been-here-before.

7. Suspected neurological conditions: recognition and referral. 26.

8. Greenhalgh, T., Knight, M., A’Court, C., Buxton, M. & Husain, L. Management of post-acute covid-19 in primary care. BMJ 370, m3026 (2020).

9. Sivan, M. & Taylor, S. NICE guideline on long covid. BMJ 371, m4938 (2020).

10. The Lancet. Facing up to long COVID. Lancet 396, 1861 (2020).

11. Patients’ experiences of ‘longcovid’ are missing from the NHS narrative. The BMJ https://blogs.bmj.com/bmj/2020/07/10/patients-experiences-of-longcovid-are-missing-from-the-nhs-narrative/ (2020).

12. Humphreys, H., Kilby, L., Kudiersky, N. & Copeland, R. Long COVID and the role of physical activity: a qualitative study. BMJ Open 11, (2021).

13. Ladds, E. et al. Developing services for long COVID: lessons from a study of wounded healers. Clin Med (Lond) 21, 59–65 (2021).

14. Reynolds, R. et al. A systematic review of chronic disease management interventions in primary care. BMC Fam Pract 19, 11 (2018).

15. Richards, T. & Scowcroft, H. Patient and public involvement in covid-19 policy making. BMJ 370, m2575 (2020).

16. Fu, V. et al. Taking Charge after Stroke: A randomized controlled trial of a person-centered, self-directed rehabilitation intervention. International Journal of Stroke 174749302091514 (2020) doi:10.1177/1747493020915144.

17. Lohse, K. R., Lang, C. E. & Boyd, L. A. Is more better? Using meta-data to explore dose-response relationships in stroke rehabilitation. Stroke 45, 2053–2058 (2014).

18. Kulnik, S. T., Pöstges, H., Brimicombe, L., Hammond, J. & Jones, F. Implementing an interprofessional model of self-management support across a community workforce: A mixed-methods evaluation study. Journal of Interprofessional Care 31, 75–84 (2017).

19. Jones, F. et al. Feasibility study of an integrated stroke self-management programme: a cluster-randomised controlled trial. BMJ Open 6, (2016).

20. Jones, F., Pöstges, H. & Brimicombe, L. Building Bridges between healthcare professionals, patients and families: A coproduced and integrated approach to self-management support in stroke. NeuroRehabilitation 39, 471–480 (2016).

21. Bodenheimer, T., Lorig, K., Holman, H. & Grumbach, K. Patient self-management of chronic disease in primary care. JAMA 288, 2469–2475 (2002).

22. Mäkelä, P., Jones, F., Abreu, M. I. de S. de, Hollinshead, L. & Ling, J. Supporting self-management after traumatic brain injury: Codesign and evaluation of a new intervention across a trauma pathway. Health Expectations 0,.

23. Taquet, M., Geddes, J. R., Husain, M., Luciano, S. & Harrison, P. J. 6-month neurological and psychiatric outcomes in 236 379 survivors of COVID-19: a retrospective cohort study using electronic health records. The Lancet Psychiatry 8, 416–427 (2021).

24. Morley, D., Dummett, S., Kelly, L. & Jenkinson, C. Measuring improvement in health-status with the Oxford Participation and Activities Questionnaire (Ox-PAQ). Patient Relat Outcome Meas 10, 153–156 (2019).

25. Ware, J. E. & Sherbourne, C. D. The MOS 36-item short-form health survey (SF-36). I. Conceptual framework and item selection. Med Care 30, 473–483 (1992).

26. Frith, J. & Newton, J. Fatigue Impact Scale. Occup Med (Lond) 60, 159–159 (2010).

27. Schwarzer R & Jerusalem M. Generalized self-efficacy scale. in Measures in health psychology: A user's portfolio. Causal and control beliefs. 35–37 (NFER-NELSON, 1995).

28. EQ-5D-5L – EQ-5D. https://euroqol.org/eq-5d-instruments/eq-5d-5l-about/.

29. Early beginnings of the CSRI | Client Service Receipt Inventory. https://www.pssru.ac.uk/csri/early-beginnings-of-the-csri/.

30. Weiner, B. J. et al. Psychometric assessment of three newly developed implementation outcome measures. Implementation Science 12, 108 (2017).

31. Avery, K. N. L. et al. Informing efficient randomised controlled trials: exploration of challenges in developing progression criteria for internal pilot studies. BMJ Open 7, e013537 (2017).

32. Moore, G. F. et al. Process evaluation of complex interventions: Medical Research Council guidance. BMJ 350, (2015).

33. Proctor, E. et al. Outcomes for Implementation Research: Conceptual Distinctions, Measurement Challenges, and Research Agenda. Adm Policy Ment Health 38, 65–76 (2011).

34. Moerbeek, M. & Wong, W. K. Sample size formulae for trials comparing group and individual treatments in a multilevel model. Statistics in Medicine 27, 2850–2864 (2008).

35. Kleinman, K. et al. clusterPower: Power Calculations for Cluster-Randomized and Cluster-Randomized Crossover Trials. (2021).

36. Flight, L. et al. Recommendations for the analysis of individually randomised controlled trials with clustering in one arm – a case of continuous outcomes. BMC Medical Research Methodology 16, 165 (2016).

37. Damschroder, L. J. et al. Fostering implementation of health services research findings into practice: a consolidated framework for advancing implementation science. Implementation Science 4, 50 (2009).

38. Waltz, T. J., Powell, B. J., Fernández, M. E., Abadie, B. & Damschroder, L. J. Choosing implementation strategies to address contextual barriers: diversity in recommendations and future directions. Implementation Science 14, 42 (2019).

39. Overview – The Consolidated Framework for Implementation Research. https://cfirguide.org/evaluation-design/overview/.
