# Supplementary material for: Effectiveness of a personalised self-management intervention for people living with long covid (Listen trial): pragmatic, multicentre, parallel group, randomised controlled trial
Source: BMJ Med. 2025 Jan 31;4(1):e001068. doi: 10.1136/bmjmed-2024-001068 (PMC11881025; doi:10.1136/bmjmed-2024-001068)
Supplement: online supplemental file 4 [file bmjmed-4-1-s004.pdf]

Statistical Analysis Plan (SAP)

Listen

LONG COVID PERSONALISED SELF-MANAGEMENT SUPPORT – CO-DESIGN AND EVALUATION (RANDOMISED CONTROLLED TRIAL)

ISRCTN number:	36407216	Version number:	1.0 (5 December 2023)

**Final Plan**
Based on protocol version: 3.0 (29 March 2022)

SAP Revision History

Protocol version	Updated SAP version number	Section number changed	Description and reason for change	Date changed
3.0	0.2	• 3.3<br>• 3.7<br>• 6.1.12<br>• 6.2.1.1<br>• 6.2.1.2.5	1. Original statement of the sample size as included in the protocol kept<br>2. Changes to the traffic light section (stopping rules)<br>3. Descriptive analysis of the pilot sample reduced	10 November 2022

			4. Secondary outcomes of the process evaluation and their statistical analysis added	
3.0	0.3		5. Text for including HEAP within SAP deleted from the introduction as the HEAP will be a separate document<br>6. Changes to the outcomes of the process evaluation and implementation<br>7. Definition of the fidelity outcome added<br>8. Descriptive analysis of the pilot sample removed<br>9. Primary and secondary outcomes edited for conciseness<br>10. Analyses of implementation measures added<br>11. Intervention fidelity analyses added	5 September 2023
3.0	0.4		12. In the covariate adjustment section, index of deprivation, severity and duration of long Covid deleted and number of long Covid symptoms at baseline added<br>13. Text related to the change in Covid symptoms deleted<br>14. Reference for PRO CoRE software added<br>15. Shell tables for the results added in the Appendix	10 October 2023
3.0	0.5		Various changes following review by PP	20 October 2023
3.0	1.0		Various changes following review by TSC statistician	5 December 2023

ROLES AND RESPONSIBILITIES

<table>
<tr><td colspan="4">Trial Statistician: Dr Muhammad Riaz</td></tr>
<tr><td colspan="4">Role: Research Fellow (Statistics), LISTEN trial statistician</td></tr>
<tr><td>Date:</td><td></td><td>Signature:</td><td></td></tr>
<tr><td colspan="4">Senior Statistician: Dr Philip Pallmann</td></tr>
<tr><td colspan="4">Role: Senior Research Fellow (Statistics), LISTEN co-investigator</td></tr>
<tr><td>Date:</td><td></td><td>Signature:</td><td></td></tr>
<tr><td colspan="4">Chief Investigators: Professor Fiona Jones and Professor Monica Busse</td></tr>
<tr><td colspan="4">Role: Co-chief investigators</td></tr>
<tr><td>Date:</td><td></td><td>Signature:</td><td></td></tr>
<tr><td colspan="4">Other non-signatory contributor to the SAP: Dr Fiona Leggat</td></tr>
<tr><td colspan="4">Role: LISTEN research associate</td></tr>
</table>

Abbreviations

**AE**	Adverse Event
**AIM**	Acceptability of Intervention Measure
**CI**	Confidence Interval
**CTR**	Centre for Trials Research
**EQ-5D-5L**	EuroQol Five Dimensions Five Levels Quality of Life Questionnaire
**EWSS**	Emotional Well-being Sub-scale Score (of the Ox-PAQ)
**FIM**	Feasibility of Intervention Measure
**FIS**	Fatigue Impact Scale
**GCP**	Good Clinical Practice
**GEE**	Generalised Estimating Equation
**GSES**	Generalised Self-Efficacy Scale
**IAM**	Intervention Appropriateness Measure
**ICC**	Intraclass Correlation Coefficient
**IQR**	Interquartile Range
**ISRCTN**	International Standard Randomised Controlled Trial Number
**ITT**	Intention to Treat
**LISTEN**	Long Covid Personalised Self-management Support – Co-design and Evaluation
**MAR**	Missing at Random
**Ox-PAQ**	Oxford Participation and Activities Questionnaire
**PPI**	Public and Patient Involvement
**RASS**	Routine Activities Sub-scale Score (of the Ox-PAQ)
**RCT**	Randomised Controlled Trial
**SAE**	Serious Adverse Event
**SAP**	Statistical Analysis Plan
**SD**	Standard Deviation
**SESS**	Social Engagement Sub-scale Score (of the Ox-PAQ)
**SF-12**	Short Form (12-item) Health Survey
**SOP**	Standard Operating Procedure

1. INTRODUCTION

This statistical analysis plan (SAP) describes the final analyses and presentation of results for the LISTEN randomised controlled trial (RCT). This plan, along with all other documents relating to the analysis of this trial, will be stored electronically in the statistical analysis master file. In line with this plan, input will be provided to the statistical sections of the funder report as well as to the principal papers that are submitted for publication in a journal. Comments and suggestions from reviewers and journal editors will be considered, and further analysis will be conducted, if necessary, in consensus with the chief investigators as far as possible in line with the principles of this SAP. In case of any deviations from this SAP, these will be described and justified in the final study report. The analysis will be conducted by the authors of this SAP or any other experienced statistician available to the trial at the time of the final analysis, who will ensure the integrity of the data during analysis processing following the strict guidelines of the Centre for Trials Research (CTR) at Cardiff University as laid out in relevant standard operating procedures (SOPs). This analysis plan has been reviewed by the senior trial statistician and agreed by the Trial Management Group before sign-off by the author and trial statistician (MR), senior trial statistician (PP), and the chief investigators (FJ and MB). A copy has been sent to the Trial Steering Committee for review, and their comments have been incorporated as appropriate.

To ensure consistency, some of the sections of this analysis plan have been directly replicated from the LISTEN study protocol (version 3.0) or the published protocol paper [1].

2. BACKGROUND

2.1 RATIONALE AND RESEARCH QUESTION

Some individuals who are infected with Covid-19 suffer from long Covid signs and symptoms and experience a wide variety of ongoing problems such as fatigue, pain, and difficulties with day-to-day tasks. This means that they may struggle to return to their former lives. This is then made worse by uncertainty and a lack of understanding by some healthcare professionals.

The LISTEN project is developing and evaluating an intervention (i.e. a package of self-management support) that has been co-designed with individuals living with long Covid to ensure that the interventions can be personalised to the individual’s needs as appropriate.

The study will recruit individuals who are living with long Covid symptoms and will be randomised to the intervention or a control group. The control group will receive usual care and if requested, the LISTEN team will signpost them to long Covid care pathways in their

region. The intervention group will receive the newly developed LISTEN resources and up to six coaching sessions from trained rehabilitation practitioners. The LISTEN intervention will be evaluated in terms of impact on participation in routine activities, emotional well-being, social participation, fatigue and self-efficacy. The impact on quality of life and cost-effectiveness of the intervention compared to usual care will also be examined. Healthcare resource use, expenses and time off work will also be recorded to understand the economic impact of long Covid and our intervention on society and individuals. The study will explore ways in which the intervention can be used across communities.

Individuals living with long Covid and a large Public and Patient Involvement (PPI) panel from diverse backgrounds have helped shape the LISTEN project. With co-design groups and an inclusion advisor, the study has recruited a PPI panel co-chaired by the study PPI co-applicant. Information about the study will be made available to participants through a variety of sources including NHS, third sector settings and community support groups and interested individuals with long Covid will be able to self-refer into the trial. The primary outcome time-point is at three months following randomisation; however, consent will be obtained for longer-term follow-up (beyond the length of the funded evaluation). An internal pilot will assess site opening and recruitment. Intervention acceptability and feasibility will be measured as part of the embedded mixed-methods process evaluation and enable a detailed analysis of implementation enablers and barriers to adoption and sustainability beyond the project timeline. This work will inform and deliver a national implementation support package (e.g. a training programme for rehabilitation teams, web platform, training manual) ready for scale-up and implementation by the end of the project. The findings of this research will be shared with the study funder, with the PPI group, in academic publications, and at conferences.

2.2 OBJECTIVES

2.2.1 PRIMARY OBJECTIVES

The primary objective is to evaluate the impact of the LISTEN co-designed personalised self-management support intervention on routine activities as assessed by the routine activities domain sub-scale of the Oxford Participation and Activities Questionnaire (Ox-PAQ).

2.2.2 SECONDARY OBJECTIVES

Secondary objectives are:

i. To evaluate the impact of the LISTEN intervention on emotional well-being as assessed by the relevant domain sub-scale of the Ox-PAQ.
ii. To evaluate the impact of the LISTEN intervention on social engagement as assessed by the relevant domain sub-scale of the Ox-PAQ.
iii. To evaluate the impact of the LISTEN intervention on health-related quality of life as assessed by the Short Form (12-item) Health Survey (SF-12).
iv. To evaluate the impact of the LISTEN intervention on fatigue as measured by the Fatigue Impact Scale (FIS).
v. To gather information on utility (using the EQ-5D-5L questionnaire) and health care resource use (using an adapted Client Service Receipt Inventory).
vi. To assess the cost-effectiveness of the LISTEN intervention.
vii. To explore key anticipated mediators of intervention outcome (namely self-efficacy in the context of Covid-19) using the Generalised Self-Efficacy Scale (GSES) with additional context-specific questions.
viii. To conduct a theory-driven detailed process evaluation within the trial using validated implementation scales to assess intervention acceptability, appropriateness, and feasibility.

2.2.3 EXPLORATORY OBJECTIVES

Additional exploratory objectives are to understand issues relating to the context, mechanisms, and outcomes of the intervention through qualitive interviews, focus groups and reflective diaries. In addition to this SAP, analysis plans will be produced for the qualitative and health-economic parts of this study by the relevant researchers.

3. STUDY MATERIALS

3.1 TRIAL DESIGN

This is a two-arm, parallel-group, open-label individually randomised controlled effectiveness trial comparing the LISTEN intervention to usual care.

3.2 RANDOMISATION

Eligible and consenting participants will be individually randomised to the LISTEN intervention or usual care arms based on a computer-generated random sequence with random permuted blocks of randomly varying sizes, created by the trial statistician in Stata version 17,

held on a secure server and implemented in the REDCap database for LISTEN at the CTR. The randomisation is stratified by the study centres, and block randomisation is used to ensure good balance between intervention and usual care within centres. The randomisation sequence is stored in the REDCap database and it is not accessible to the trial manager, data manager and those responsible for randomisation at the CTR or centres. Participants will be allocated to the two trial arms in the ratio of 1:1. The trial staff will be able to access the allocation for each participant via a secure online randomisation system implemented in REDCap. Participants will not know their allocation to intervention or usual care until they have completed all baseline questionnaires and medical assessments. Thus, the allocations will be concealed until a participant has been assigned to an arm and recruitment as well as baseline data collection are complete.

3.3 SAMPLE SIZE

We aim to detect a minimum clinically important effect size of 0.32 between randomised arms in the primary outcome of the routine activities domain of the Ox-PAQ [3] with 90% power whilst controlling the two-sided type I error level at 5%. A conventional individually randomised trial would require 414 participants (based on a two-sample t-test), but since the intervention will be delivered by up to 24 community rehabilitation teams, we must also take potential clustering in the intervention arm into account. Assuming an intraclass correlation coefficient (ICC) of 0.03 in the intervention arm, 24 clusters with 10 participants each in the intervention arm and 234 participants in the usual care arm (i.e. a total of 474 participants) are required for 90% power. This was calculated using the method of Moerbeek and Wong [4] as implemented in version 0.7.0 of the R package 'clusterPower' [5]. Assuming 15% loss to follow-up, the overall recruitment target is 558.

3.4 FRAMEWORK

This is a superiority trial testing whether the LISTEN intervention is superior to usual care.

3.5 INTERIM ANALYSES

We do not plan for any formal interim analysis. An internal pilot study was performed at the end of trial month 12 (anticipated in July 2022) to assess feasibility based on a simple descriptive analysis of recruitment figures.

3.6 PLANNED SAMPLE SIZE ADJUSTMENT

Not applicable

3.7 STOPPING RULES

The internal pilot assessed site opening and recruitment at the end of month 12 of the trial. A traffic light system (green, amber, red) of progression criteria as proposed by Avery et al [6] was used to guide decision-making with green resulting in the trial continuing as planned, amber requiring changes for the trial to continue, and red stopping the trial.

3.8 TIMING OF FINAL ANALYSIS

The end of the trial is defined as the date of final data capture to meet the trial endpoints. In this case, end of the trial is defined as the date on which data for all participants is frozen after the last participant has had their 3-month follow-up and once the mixed-method process evaluation has been completed. Consent for long-term follow-up will also be sought, and any long-term follow-up will continue after the trial is regarded as completed.

All statistical analyses for the final report of the study will be conducted when the trial database is locked for further entries after the 3-month follow-up has been completed for all participants.

3.9 TIMING OF OUTCOME ASSESSMENT

All primary and secondary outcomes will be assessed at baseline and at 3 months follow-up. A complete data collection schedule is provided below.

**Procedures**	**Data collection time points**		
	**Baseline**	**6-week interim data collection**	**3-month follow-up**
**Demographics**	X		
**Long Covid history**	X		
**Ox-PAQ questionnaire**	X		X
**SF-12 questionnaire**	X		X
**FIS questionnaire**	X		X
**EQ-5D-5L questionnaire**	X	X	X
**GSES questionnaire**	X		X
**Health service use questionnaire**	X	X	X

Procedures	Data collection time points		
	**Baseline**	**6-week interim data collection**	**3-month follow-up**
**AIM questionnaire**			X
**IAM questionnaire**			X
**FIM questionnaire**			X
**Semi-structured interviews**			X
**SAE reporting**		X	X

4. STATISTICAL PRINCIPLES

4.1 LEVELS OF CONFIDENCE AND P-VALUES

A two-sided type I error level of 5%, corresponding to a two-sided 95% confidence level, will be used for the statistical analysis. When reporting the results, we will present point estimates, 95% confidence intervals (CIs) and p-values. Hence a p-value <0.05 will imply that a test result is statistically significant, and this should be supported by the relevant 95% CI.

4.2 ADJUSTMENT FOR MULTIPLICITY

Keeping in view the study design and a single primary outcome of interest, we do not need any adjustment for multiple testing in the main analysis. The secondary subgroup analyses will be exploratory and interpreted with caution, thus no multiplicity adjustment is required.

4.3 ADHERENCE AND PROTOCOL DEVIATIONS

Non-adherence and protocol deviations will be handled according to CTR SOP/009/5 (Protocol/GCP non-compliance and serious breaches, version 4.0).

4.4 DEFINITION AND ASSESSMENT OF ADHERENCE

If a participant deviates from the assigned intervention regimen, this is considered non-adherence. For example, if a participant does not attend some or all sessions of the intervention or does not comply with the intervention as described in the study protocol, these will be recorded as partial or complete non-adherence, respectively.

Furthermore, in order to confirm intervention fidelity, an independent analysis of 10% of the planned sample will be carried out based on data from one-to-one remote self-management coaching sessions delivered in intervention sites, recorded via Zoom or Teams depending on

participants' preference. These will be reviewed against pre-defined fidelity markers. We will capture reflections from the training delivery team about methods used to engage and sustain fidelity of intervention delivery, through the completion of online reflective journals. This work will be performed by the LISTEN research associate (FL); the final analysis of the fidelity markers will be performed by the trial statistician (MR).

4.5 PRESENTATION OF ADHERENCE

Non-adherence to the assigned study intervention, including number of sessions missed, will be summarised descriptively by arm.

4.6 DEFINITION OF PROTOCOL DEVIATION

Non-compliances of GCP and/or protocol will be categorised as either a deviation, violation or serious breach according to CTR SOP/009/5.

A planned or unplanned departure from the study protocol that does not increase risk or decrease benefit or does not have a significant impact on the participant's rights, safety or welfare; and/or on the integrity of the data is called a protocol deviation.

An unplanned departure from the protocol or GCP that increases the risk or decreases the benefit or; may have an impact on the participant's rights, safety or welfare; and/or on the integrity of data, is called a protocol violation.

A breach of the protocol or GCP which is likely to significantly affect the safety or physical or mental integrity of the trial participants or the scientific value of the trial is known as a serious breach.

This is not a trial of an investigational medicinal product, and we do not expect any serious breaches and violations.

4.7 PRESENTATION OF PROTOCOL DEVIATIONS

Any deviations, violations, and serious breaches will be summarised descriptively by arm.

4.8 ANALYSIS POPULATION

The trial population will consist of non-hospitalised individuals living with long Covid in England and Wales. Recruitment will be inclusive of age, gender, ethnic and disability groups through primary care. All attempts will be made to reflect current data on people experiencing

long Covid and include people across age, ethnic groups and those with and without previous long-term conditions, people working in health and social care.

The primary analysis will be conducted as intention-to-treat (ITT), meaning that all participants with available outcome data will be analysed based on their allocation as determined by the randomisation, regardless of level of adherence. In a secondary per-protocol analysis we will only include intervention arm participants who completed all, or completed at least 50%, of their sessions, respectively. The analysis of harms will be conducted using a safety population where only participants who received at least some element of the intervention are included in the intervention arm, and everyone else in the control arm.

5. STUDY POPULATION

5.1 SCREENING DATA

A screening log will be generated centrally by the LISTEN database and the screening data will be stored in the database.

5.2 ELIGIBILITY

Participants will be eligible for inclusion if they:

- Are aged ≥18 years **AND**
- Experience persistent illness (at least one long Covid symptom for ≥12 weeks) **AND**
- Had a positive SARS-CoV-2 PCR or antigen test (positive Covid-19 test) during the acute phase of illness **OR**
- Had a positive SARS-CoV-2 antibody test (positive Covid-19 antibody test) at any time point in the absence of SARS-CoV-2 (Covid-19) vaccination history **OR**
- Had a loss of sense of smell or taste during the acute phase in the absence of any other identified cause **OR**
- Had symptoms consistent with SARS-CoV-2 (Covid-19) infection during the acute phase and there was a high prevalence of Covid-19 at time and location of onset **OR**
- Had at least one symptom consistent with SARS-CoV-2 (Covid-19) infection during the acute phase **AND** close contact of a confirmed case of Covid-19 around the time of onset.

5.3 RECRUITMENT

A detailed recruitment plan is available in section 9 of protocol version 3.0.

The number of participants recruited at each centre and broken down by method of recruitment (mail-out, routine clinic, publicity, outward facing communication), randomised to intervention or usual care, lost/withdrawn, followed-up and included in the final analysis (by the study arms) will be summarised in a CONSORT flow diagram.

5.4 WITHDRAWAL/FOLLOW UP

5.4.1 WITHDRAWAL

Participants have the right to withdraw consent for participation in any aspect of the study at any time. The participant's care will not be affected at any time by declining to participate or withdrawing from the trial. If a participant initially consents but subsequently withdraws from the study, a clear distinction will have to be made regarding what aspect(s) of the study the participant is withdrawing from. These aspects could be:

- Withdrawal from intervention
- Partial withdrawal from further data collection
- Complete withdrawal from further data collection
- Withdrawal of permission to use data already collected

The withdrawal of a participant consent shall not affect the trial activities already carried out and the use of data collected prior to participant withdrawal (unless they withdraw from this as well). The use of the data collected prior to the withdrawal of consent is based on informed consent before its withdrawal.

Furthermore, it is important to collect safety data ongoing at the time of withdrawal, especially if the participant withdraws because of a safety reason. There is specific guidance on this contained in the participant information sheet but briefly, a participant may withdraw or be withdrawn from the trial intervention for non-compliance or intolerance to the intervention.

In all instances, participants who consent and subsequently withdraw should complete a withdrawal CRF on the LISTEN database or the withdrawal form should be completed on the participant's behalf by the researcher/clinician based on information provided by the participant.

5.4.2 LOSS TO FOLLOW-UP

As stated in the protocol, every effort will be made to reduce the rate of loss to follow-up using the methods listed below:

i. We will emphasise the importance of getting follow-up data to all participants at baseline and the follow-up assessment.
ii. Participants will have two weeks prior and two weeks after follow-up measure time points to complete the assessments (questionnaires). Automated reminders will be sent two weeks ahead of the assessment due date. If the assessments are not completed within two weeks after the due date, the trial team will telephone the participant to prompt outcome measure completion.
iii. We will invite a selection of participants to interview (process evaluation) at the initial registration and gather information on the most suitable day and time for any follow-up interviews.
iv. For the interviews, up to five attempts will be made to contact a participant to arrange a date for their interview.

5.5 LEVEL OF WITHDRAWAL

See 5.8

5.6 TIMING OF WITHDRAWAL

See 5.8

5.7 REASONS FOR WITHDRAWAL

See 5.8

5.8 PRESENTATION OF WITHDRAWAL/LOSS TO FOLLOW-UP

Level, timing, and reasons for participant withdrawals will be summarised descriptively by arm.

5.9 BASELINE PARTICIPANT CHARACTERISTICS

See 5.10

5.10 LIST OF BASELINE DATA

- Study centre

- Age
- Gender
- Ethnicity
- Education level
- Employment status
- Socioeconomic status (index of multiple deprivation, if available)
- Weight and height (or body mass index)
- Long Covid history
- Ox-PAQ questionnaire at baseline
- SF-12 questionnaire at baseline
- FIS questionnaire at baseline
- EQ-5D-5L questionnaire at baseline
- GSES questionnaire at baseline
- Health service use questionnaire at baseline

5.11 DESCRIPTIVE STATISTICS

Normality of continuous variables will be examined using histograms or boxplots. As appropriate, continuous variables will then be summarised using mean and standard deviation (SD) or median and interquartile range (IQR) while categorical variables will be summarised using frequency and percentage (%). Number (%) of missing values will also be reported for each variable. All these will be presented by arm so that any differences of the baseline variables can be examined descriptively. The baseline data may also be plotted using appropriate methods such as bar graphs, histograms, and boxplots.

We do not anticipate a considerable amount of missing data in this study, but if there is substantial missingness due to withdrawal or loss to follow-up, summary statistics of the baseline variables will be presented separately for those with and without missing follow-up data, respectively, as well as by arm.

6. ANALYSIS

6.1 OUTCOMES DEFINITIONS

6.1.1 PRIMARY OUTCOME

See 6.1.2

6.1.2 TIMING, UNITS AND DERIVATION OF PRIMARY

The Ox-PAQ is a 23-item questionnaire completed by participants for assessing participation and activity in people experiencing a health condition such as COVID-19 [7-11]. It has three main domains, routine activities (14 items), emotional well-being (5 items), and social engagement (4 items). The psychometric analysis of Ox-PAQ has shown that it is a valid and reliable scale for assessing self-reported activity [11].

The primary outcome will be the routine activities domain, which will be collected at baseline and 3 months follow-up. It is comprised of doing household chores, going to shops, physical activities for enjoyment, daily activities, getting around the home, being independent, getting dressed, doing work (paid or unpaid), using public transport, engaging in community life, using own transport, social life, leisure activities, and getting up in the morning. Each item is rated from 0 (never) to 4 (always). A final routine activities sub-scale score (RASS) based on the 14 items will be computed as follows [11]:

$$RASS = \left(\frac{Sum\ of\ ratings\ of\ items\ 1\ to\ 14\ on\ \text{routine activities}\ domain}{4 * 14}\right) * 100$$

As described in the Ox-PAQ scoring manual, if only one item on any domain is missing for a participant, it will be imputed with mean value of all other items to compute the total score. If two or more items of a domain are missing for a participant, the total score will not be computed and will be considered as missing for that participant.

6.1.3 LIST OF SECONDARY OUTCOMES

See 6.1.5

6.1.4 ORDER OF TESTING

Not applicable

6.1.5 TIMING, UNITS AND DERIVATION OF SECONDARY OUTCOMES

Secondary outcomes will include participants' emotional well-being, social engagement, health-related quality of life, fatigue, health utility, and self-efficacy. These outcomes will be measured at baseline and 3 months follow-up by the emotional well-being and social engagement sub-scales of the Ox-PAQ [11], the SF-12 [12], FIS [13], EQ-5D-5L [14-18], and GSES [19]. For all domains of the Ox-PAQ, missing data will be dealt with as described in 6.1.2. A similar rule will be followed for the other questionnaires.

Emotional well-being (Ox-PAQ)

The emotional well-being domain of the Ox-PAQ consists of 5 items: anxiety, sadness, depression, stress, and control over one's life. All these items are rated from 0 (never) to 4 (always). An emotional well-being sub-scale score (EWSS) will be computed as follows and will be used as an outcome for assessing a participant's emotional well-being [11]:

$$EWSS = \left(\frac{Sum\ of\ ratings\ of\ items\ 1\ to\ 5\ on\ \text{emotional wellbeing}\ domain}{4 * 5}\right) * 100$$

Social engagement (Ox-PAQ)

The social engagement domain of the Ox-PAQ consists of 4 items: communicating with others, engaging in the community, maintaining friendships, and maintaining close relationships. All these items are rated from 0 (never) to 4 (always). A social engagement sub-scale score (SESS) will be computed as follows and will be used as an outcome for assessing a participant's social engagement [11]:

$$SESS = \left(\frac{Sum\ of\ ratings\ of\ items\ 1\ to\ 4\ on\ \text{social engagement}\ domain}{4 * 4}\right) * 100$$

SF-12

This 12-item short form is a multi-purpose short survey which was adopted from the SF-36 Health Survey [12, 20-21]. It is comprised of two main components, physical and mental functioning. To examine the mental and physical functioning of participants and their overall health-related quality of life, rated responses to the questions are combined to create total score for the two components and an overall scale with weighting method as described in [12].

FIS

The FIS is a self-reported 40-item instrument comprised of three subscales for examining participants' perceived impact of fatigue on quality of life. The subscales are cognitive functioning (10 items), physical functioning (10 items), and psychosocial functioning (20 items). Each item is rated on a scale from 0 (no problem) to 4 (extreme problem) by the participant, which indicate the extent to which the fatigue has caused problems for them. A total score for the three subscales and overall scale (maximum score = 160) is computed to examine the impact of fatigue [13].

EQ-5D-5L

The EQ-5D [14-18] is a 5-item questionnaire with an additional visual analog scale from 0 to 100 (0=worst health and 100=best health) to rate the general health state of a participant. The visual analog scale gives a quantitative measure of the participant's self-reported state of their overall health. There have been a few versions of EQ-5D and the latest version is the EQ-5D-5L, which is an improved descriptive system of the same five dimensions (mobility, self-care, usual activities, pain/discomfort, and anxiety/depression), but each dimension is now rated with five levels: 1. no problems, 2. slight problems, 3. moderate problems, 4. severe problems, 5. unable to/extreme problems. Participants rate their health status on the scale by selecting an appropriate response out of the above mentioned five levels [14]. The responses for a participant are combined to determine their health state.

GSES

The GSES is used to assess optimistic self-belief [19-22]. This is usually self-administered and examines the belief of a participant regarding how well they can perform a novel or complicated task or can handle a difficult situation in various domains of functioning. This scale can be interpreted in the domains of goal setting, effort investment, resilience, and recovery from any issue. It is comprised of 10 items and the response to each item is rated on a 4-point scale from 1 to 4. A total score is created by summing up the responses to all 10 items, ranging from 10 to 40. The scale can be used to assess a participant's subsequent behaviour or change in their behaviour and clinical practice [19].

Intervention implementation measures

The intervention process-related measures will be the perceptions of acceptability, appropriateness and feasibility of the use and implementation of the LISTEN intervention. These will be measured by the Acceptability of Intervention Measure (AIM), Intervention Appropriateness Measure (IAM), and Feasibility of Intervention Measure (FIM) questionnaires in both participants and intervention providers (practitioners) [1-2]. These three measures consist of four items each, and each item is rated on scale from 1 to 5. A total score for each measure is produced by summing up the response to all four items, with possible scores ranging from 4 to 20. Higher scores on the AIM, IAM, and FIM indicate stronger participant or provider perceptions of acceptability, appropriateness, and feasibility of the LISTEN intervention or routine care, respectively.

LISTEN intervention fidelity measure

A sub-sample of 10% of all LISTEN intervention sessions delivered are recorded for observations. Observations of the recorded intervention sessions are undertaken using an 8-item fidelity checklist, featuring eight pre-defined skills focusing on practitioner language and behaviour. These skills were developed through the LISTEN intervention co-design phase [2] and the underlying LISTEN programme theory [1]. The core skills used by LISTEN practitioners include: 1) attentive listening, 2) hearing beyond words and not rushing to fix, 3) being curious about each individual and their story, 4) exploring how the individual can feel in control, 5) using language that explores how the individual can feel success, 6) using language that helps individuals reflect on what has worked and how, 7) exploring individuals hopes and fears, and 8) being alive to the possibilities of finding joy and a new identity after long Covid.

The use of the co-designed LISTEN handbook during sessions will also be observed. By observing integration of the handbook through the lens of the eight pre-defined criteria, this will monitor participants' preference to use or not use the book as part of their LISTEN intervention experience. Participant preference and tailoring of sessions to participants' needs and preferences is a core component of the LISTEN intervention, and therefore, the decision to use or not use sections of the handbook in each session will be evaluated separately to the eight core skills. Examples of the pre-determined skills, including use of the handbook, have been provided to LISTEN intervention practitioners as part of their training and ongoing support package.

Each of the core skills will be rated by the observer on a 3-point Likert scale from 1 to 3 (1=not observed, 2=partially/inconsistently observed, 3=consistently observed). The sum of all 8 items will give a total fidelity score, assigned to the LISTEN intervention practitioner delivering the sessions.

For consistency, recorded intervention sessions will be observed by the same member of the research team (FL) who was involved in delivering the LISTEN intervention training to practitioners. To ensure face validity of the rating system with the checklist, 20% of all recorded sessions will be observed by additional member of the research team (FJ or ATB). Following separate ratings of the pre-defined skills, all observers will meet to compare and discuss their scores until a consensus is reached.

6.2 ANALYSIS METHODS

6.2.1 LIST OF METHODS AND PRESENTATION

6.2.1.1 Unit of analysis

For all outcome analyses assessing the effect of the intervention on long Covid, individual participants randomised to the trial arms will be the unit of analysis. For measures related to healthcare practitioners such as AIM, IAM, and FIM, individual practitioners will be the unit of analysis. For the healthcare practitioner variables, the analysis methods will be similar to the participant versions of these variables.

6.2.1.2 Description of the overall trial sample by arm

Participant characteristics at baseline will be summarised descriptively by arm using descriptive statistics as described in section 5.11.

6.2.1.3 Primary outcome analysis

The aim of the primary analysis will be to examine the effectiveness of the intervention in terms of the change in the Ox-PAQ RASS from baseline to follow-up. For each participant, the RASS will be computed based on the 14 items of the routine activities domain, both at baseline and at 3 months follow-up. Depending on the distribution of the RASS, mean (SD) or median (IQR) of the score and its change from baseline to 3 months will be computed and presented by arm.

The primary outcome analysis will be performed using the ITT population. We will use a linear mixed-effect regression model [23-25] with RASS at 3 months as dependent variable, randomisation group and baseline RASS as independent variables, and a random centre effect. From this model, we will present the estimated mean difference of the RASS at 3 months for the intervention arm compared with the usual care arm alongside a 95% CI. This regression model will take into account clustering due to centres in both arms. Further covariate adjustments will be carried out as described in section 6.2.2.

6.2.1.4 Secondary outcome analysis

The effectiveness of the intervention on the total scores of the secondary outcomes (Ox-PAQ EWSS and SESS, SF-12, FIS, EQ-5D-5L, GSES) at 3 months follow-up, adjusted for baseline, will be assessed using similar statistical methods (linear mixed-effect modelling) as described above for the primary outcome analysis [24]. The estimated adjusted difference of mean total scores of these secondary outcomes at 3 months follow-up between the two arms and 95% CIs will be computed from linear mixed-effect models. Similar adjustments for clustering due to

centre as described above for the primary outcome analysis will be carried out and further covariates adjustment will be considered as detailed in section 6.2.2.

6.2.1.5 Implementation measure analysis

The LISTEN intervention process evaluation-related outcomes assess the perceptions of acceptability, appropriateness and feasibility of the use and implementation of the intervention. For participants allocated to the intervention arm an implementation total score (range: 12-60) based on the AIM score (range: 4-20), IAM score (range: 4-20), and FIM score (range: 4-20) [52] will be computed and summarised using mean (SD) or median (IQR) as appropriate. The distribution of the score will be examined using histograms or boxplots for assessing normality. Pearson correlation coefficient or Spearman rank correlation coefficient will be used to examine the relationship of the implementation total score and the AIM, IAM, and FIM scores, respectively, with the primary outcome measure (Ox-PAQ RASS) at 3 months follow-up, or its change from baseline to follow-up. Similarly, these scores' relationships with the secondary outcomes will be examined using the same methods. The relationship of the implementation total score with baseline measures such as age, gender and ethnicity will also be explored using appropriate statistical methods such as Pearson or Spearman correlation coefficients. Next, the analysis of the relationship of the total score with outcomes of interest will be adjusted for the clustering effect of study centres and other baseline measures such as age, gender and number of long Covid symptoms using linear mixed-effect models (or generalised estimating equations (GEE) if the assumptions for linear mixed-effect models are not met [41]) with the implementation total score as dependent variable.

6.2.1.6 Intervention fidelity analysis

We will summarise the intervention fidelity measure using mean (SD) or median (IQR) of the fidelity score as appropriate. In a sub-sample, an appropriate method of computing a correlation coefficient (e.g. Pearson, Spearman's rho, or Kendall's tau, depending on the distribution of the variable) will be used to examine the relationship of the fidelity score with the primary outcome measure, Ox-PAQ RASS at 3 months follow-up, or its change from baseline to follow-up. Similarly, the fidelity score's relationship with the secondary outcomes will be examined using the same methods. Correlation of the fidelity score with baseline measures such as age will also be explored. The analysis of the relationship of the fidelity score with outcomes of interest will be adjusted for the clustering effect of study centres using linear mixed-effect models (or GEE if key assumptions are not met [41]) with the outcome of interest

as dependent variable and the fidelity score as independent variable. This analysis will determine the effect of the quality of intervention delivery (fidelity score) on outcomes of interest [42-43].

6.2.2 COVARIATE ADJUSTMENT

We will have an a priori decided set of covariates including age, gender, ethnicity, BMI, employment status, and number of long Covid symptoms [26-32]. We will adjust the primary and secondary outcome analyses for these a priori selected covariates in the linear mixed-effect regression models as described in sections 6.2.1.3 and 6.2.1.4.

6.2.3 ASSUMPTION CHECKING

See 6.2.4

6.2.4 ALTERNATIVE METHODS IF DISTRIBTIONAL ASSUMPTIONS NOT MET

Before the analyses, the distributions of the primary and secondary outcome measures will be examined using histograms or boxplots. If there are any substantial departures from normality, transformations (e.g. logarithmic) will be attempted [33]. If transformations do not improve the distributions of the outcome scores, assumptions of other suitable distributions (e.g. log-normal, Poisson, or negative binomial) will be considered. If the assumption of a suitable distribution is not appropriate for the continuous outcomes, non- or semi-parametric statistical methods (e.g. GEE [34-36], quantile regression [37-38], bootstrapping [39]) will be considered. If a dichotomous or categorical version of an outcome is used, the use of mixed-effect logistic [24, 40] or multinomial logistic regression [40-42] will be considered.

6.2.5 SENSITIVITY ANALYSES

As the intervention is delivered by multiple community rehabilitation teams within each centre, it can be assumed that there might be additional clustering in the intervention arm but not in the usual care due to ‘practitioner effects’, on top of the clustering in both arms due to centres. Therefore, as sensitivity analyses, we plan to use the regression modelling approaches described in [43-44]. The method is comprised of two main approaches to mixed-effect modelling for handling a ‘no (additional) clustering’ scenario in the usual care group: 1. Partially nested homoscedastic regression model, and 2. Partially nested heteroscedastic regression model. In the partially nested homoscedastic model, a random-effects term will

account for the between-cluster variation only in the clustered arm (intervention). In the partially nested heteroscedastic regression model, we will account for two sources of variation in relation to the randomisation groups (intervention and usual care): a random effect for the between-cluster variation in the clustered arm (intervention), and another random effect for the individual-level variation in the non-clustered arm (usual care) [43-44]. The rest of the model structure will be the same as described in section 6.2.1.3 and we will estimate the difference in the primary outcome at 3 months adjusted for baseline for the intervention arm compared with usual care.

Further sensitivity analysis will be conducted based on a per-protocol population as defined in section 4.8 and, if missingness at random can reasonably be assumed, using multiple imputation to investigate the impact of missing observations in outcomes and other variables as described in section 6.2.7.

6.2.6 SUBGROUP ANALYSES

We will investigate the modification of effects if the intervention is effective compared with usual care. To this end, the covariates listed in 6.2.2 (age, gender, ethnicity, BMI, employment status, and number of long Covid symptoms) will be assessed as effect modifiers in the mixed-effect models by adding the relevant interaction terms [45]. If an interaction term is statistically significant, further (exploratory) subgroup analyses will be performed.

If data is available, we will perform exploratory subgroup analyses for type and intensity of the usual care received, and for medication or other therapies participants used to relieve long Covid symptoms. All effect estimates will be presented as adjusted mean differences with CIs.

6.2.7 MISSING DATA

Missingness is likely to occur in the primary and secondary outcomes as well as in independent variables of interest other than the randomisation arms. The quantity and distribution of missing data will be determined. In addition to the primary complete case analysis, if there is a non-negligible amount of missing values, multiple imputation with the assumption of missingness at random (MAR) will be considered to deal with missing observations. Analysis will be conducted to assess the assumption of MAR and identify baseline variables to be used in the imputation model for the primary and secondary outcomes separately. Missing observations in the primary and secondary outcomes, and all other variables with missing observations will be replaced by the imputed values using chained equations [46-47] of linear regression. At least

20 datasets will be created for the imputation of each outcome and any other variables with missing data [48-49] and the imputation-specific estimates for the effect of intervention on the primary and secondary outcome will be combined using Rubin's rules [50-51].
These analyses will be considered as sensitivity analyses, and all the above stated analyses will be reconducted with missing observations replaced by the imputed values, and the results will be compared with the ITT and complete case analyses.

6.2.8 ADDITIONAL ANALYSES

The health economic and qualitative analyses plans are separate from this SAP.

6.2.9 HARMS

This is not a trial of an investigational medicinal product, and it is unlikely that there will be many adverse events (AEs) and serious adverse events (SAEs). Frequency (%) of each type of AE and SAE, as well as total numbers of AEs and SAEs, will be tabulated by arm and compared between the two arms (using the safety population as defined in 4.8) using chi-square tests, if numbers allow. In addition, frequency (%) of each type of problem that the participants faced during the trial (as collected in the LISTEN problem questionnaire) will be presented by arm and compared between the two arms using chi-square tests, if numbers allow.

6.2.10 STATISTICAL SOFTWARE

The main software packages used for the statical analysis will be Stata version 17 or higher (StataCorp LLC, College Station, TX, USA) or R version 4.1.2 or higher (R Foundation for Statistical Computing, Vienna, Austria) via RStudio (RStudio, Boston, MA, USA). Other statistical software packages such as PRO CoRE version 2.1 or higher (Optum Inc, Johnston, RI, USA) may also be used if needed.

7. REFERENCES

7.1 NON-STANDARD STATISTICAL METHODS

1. Potter C, Leggat F, Lowe R, et al. Effectiveness and cost-effectiveness of a personalised self-management intervention for living with long COVID: protocol for the LISTEN randomised controlled trial. Trials. 2023;24:75.

2. Heaton-Shrestha C, Torrens-Burton A, Leggat F, et al. Co-designing personalised self-management support for people living with long Covid: the LISTEN protocol. PLoS One. 2022;17:e0274469.
3. Morley D, Dummett S, Kelly L, Jenkinson C. Measuring improvement in health-status with the Oxford Participation and Activities Questionnaire (Ox-PAQ). Patient Related Outcome Measures. 2019;10:153-156.
4. Moerbeek M, Wong WK. Sample size formulae for trials comparing group and individual treatments in a multilevel model. Statistics in Medicine. 2008;27:2850-2864.
5. Kleinman K, Sakrejda A, Mojer J, et al. clusterPower: Power calculations for cluster-randomized and cluster-randomized crossover trials. R package version 0.7.0. 2021. https://CRAN.R-project.org/package=clusterPower
6. Avery KNL, Williamson PR, Gamble C, et al. Informing efficient randomised controlled trials: exploration of challenges in developing progression criteria for internal pilot studies. BMJ Open. 2017;7:e013537.
7. https://www.ndph.ox.ac.uk/research/health-services-research-unit-hsru/research/oxpaq-initiative
8. Kelly L, Jenkinson C, Dummett S, et al. Development of the Oxford Participation and Activities Questionnaire: constructing an item pool. Patient Related Outcome Measures. 2015;6:145-155.
9. Morley D, Dummett S, Kelly L, et al. The Oxford Participation and Activities Questionnaire: study protocol. Patient Related Outcome Measures. 2013:11;1-6.
10. Morley D, Dummett S, Kelly L, et al. Validation of the Oxford Participation and Activities Questionnaire. Patient Related Outcome Measures. 2016;7:73-80.
11. Morley D, Dummett S, Kelly L, et al. Ox-PAQ User Manual. Oxford: Health Services Research Unit; 2016. https://innovation.ox.ac.uk/wp-content/uploads/2016/08/Ox-PAQ_UserManual_contents.pdf
12. Ware JE Jr, Kosinski M, Keller SD. How to Score the SF-12 Physical and Mental Health Summary Scales. 3rd edition. Boston, MA: The Health Institute, New England Medical Center; 1998.
13. Fisk JD, Ritvo PG, Ross L, et al. Measuring the functional impact of fatigue: initial validation of the Fatigue Impact Scale. Clinical Infectious Diseases. 1994;18:S79-83.

14. Janssen MF, Bonsel G, Luo N. Is EQ-5D-5L better than EQ-5D-3L? A head-to-head comparison of descriptive systems and value sets from seven countries. Pharmacoeconomics. 2018;36:675-697.
15. EuroQol Group. EuroQol – a new facility for the measurement of health related quality of life. Health Policy. 1990;16:199-208.
16. Hurst N, Kind P, Ruta D, et al. Measuring health-related quality of life in rheumatoid arthritis: validity, responsiveness and reliability of EuroQol (EQ-5D). British Journal of Rheumatology. 1997;36:551-559.
17. Devlin NJ, Brooks R. EQ-5D and the EuroQol Group: past, present and future. Applied Health Economics and Health Policy. 2017;15:127-137.
18. EuroQol Research Foundation. EQ-5D-5L User Guide. 2019. https://euroqol.org/publications/user-guides
19. Schwarzer R, Jerusalem M. Generalized Self-Efficacy Scale. In Weinman J, Wright S, Johnston M: Measures in Health Psychology: a User's Portfolio. Causal and Control Beliefs (pp. 35-37). Windsor, UK: NFER-Nelson; 1995.
20. Ware JE Jr, Sherbourne CD. The MOS 36-item short-form health survey (SF-36). I. Conceptual framework and item selection. Medical Care. 1992;30:473-483.
21. Ware JE Jr. SF-36 Health Survey Manual and Interpretation Guide. Boston, MA: The Health Institute New England Medical Center; 1996.
22. Jerusalem M, Schwarzer R. Self-efficacy as a resource factor in stress appraisal processes. In Schwarzer R (ed.), Self-efficacy: Thought Control of Action (pp. 195-213). Washington, DC: Hemisphere; 1992.
23. McCulloch CE, Searle SR. Generalized, Linear, and Mixed Models. New York, NY: John Wiley and Sons; 2000.
24. Riaz M, Lewis S, Coleman T, et al. Which measures of cigarette dependence are predictors of smoking cessation during pregnancy? Analysis of data from a randomized controlled trial. Addiction. 2016;111:1656-1665.
25. Potthoff RF, Roy SN. A generalized multivariate analysis of variance model useful especially for growth curve problems. Biometrika. 1964;51:313-326.
26. Tleyjeh IM, Kashour T, Riaz M, et al. Persistent COVID-19 symptoms at least one month after diagnosis: a national survey. Journal of Infection and Public Health. 2022;15:578-585.

27. Alkodaymi MS, Omrani OA, Fawzy NA, et al. Prevalence of post-acute COVID-19 syndrome symptoms at different follow-up periods: a systematic review and meta-analysis. Clinical Microbiology and Infection. 2022;28:657-666.
28. Tleyjeh IM, Saddik B, AlSwaidan N, et al. Prevalence and predictors of post-acute COVID-19 syndrome (PACS) after hospital discharge: a cohort study with 4 months median follow-up. PloS One. 2021;16:e0260568.
29. Xiong Q, Xu M, Li J, et al. Clinical sequelae of COVID-19 survivors in Wuhan, China: a single-centre longitudinal study. Clinical Microbiology and Infection. 2021;27:89-95.
30. Sudre CH, Murray B, Varsavsky T, et al. Attributes and predictors of long COVID. Nature Medicine. 2021;27:626-631.
31. Munblit D, Bobkova P, Spiridonova E, et al. Incidence and risk factors for persistent symptoms in adults previously hospitalized for COVID-19. Clinical and Experimental Allergy. 2021;51:1107-1120.
32. Augustin M, Schommers P, Stecher M, et al. Post-COVID syndrome in non-hospitalised patients with COVID-19: a longitudinal prospective cohort study. The Lancet Regional Health Europe. 2021;6:100122.
33. Ussher M, Lewis S, Aveyard P, et al. Physical activity for smoking cessation in pregnancy: randomized controlled trial. BMJ. 2015;350:h2145.
34. Hardin J, Hilbe J. Generalized Estimating Equations. London, UK: Chapman and Hall/CRC; 2003.
35. Aloisio KM, Micali N, Swanson SA, et al. Analysis of partially observed clustered data using generalized estimating equations and multiple imputation. Stata Journal. 2014;14:863-883.
36. Cabanillas OB, Michler JD, Michuda A, Tjernstrom E. Fitting and interpreting correlated random coefficient models using Stata. Stata Journal 2018;18:159-173.
37. Higham N. Computing the nearest correlation matrix – a problem from finance. IMA Journal of Numerical Analysis. 2002;22:329-343.
38. Pinheiro J, Bates D, DebRoy S, et al. nlme: Linear and nonlinear mixed effects models. R package version 3.1-117. 2014. https://CRAN.R-project.org/package=nlme
39. Freedman DA. Bootstrapping regression models. Annals of Statistics. 1981;9:1218-1228.
40. Menard S. Applied Logistic Regression Analysis. Thousand Oaks, CA: Sage; 2002. p. 91.
41. Greene WH. Econometric Analysis. 7th edition. Boston, MA: Pearson Education; 2012. pp. 803-806.

42. Engel J. Polytomous logistic regression. Statistica Neerlandica. 1988;42:233-252.
43. Flight L, Allison A, Dimairo M, et al. Recommendations for the analysis of individually randomised controlled trials with clustering in one arm – a case of continuous outcomes. BMC Medical Research Methodology. 2016;16:165.
44. Candlish J, Teare MD, Dimairo M, et al. Appropriate statistical methods for analysing partially nested randomised controlled trials with continuous outcomes: a simulation study. BMC Medical Research Methodology. 2018;18:105.
45. Aiken LS, West SG. Multiple Regression: Testing and Interpreting Interactions. Newbury Park, CA: Sage; 1991.
46. Azur MJ, Stuart EA, Frangakis C, Leaf PJ. Multiple imputation by chained equations: what is it and how does it work? International Journal of Methods in Psychiatric Research. 2011;20:40-49.
47. Van Buuren S, Boshuizen HC, Knook DL. Multiple imputation of missing blood pressure covariates in survival analysis. Statistics in Medicine. 1999;30:681-694.
48. Daley A, Riaz M, Lewis S, et al. Physical activity for antenatal and postnatal depression in women attempting to quit smoking: randomised controlled trial. BMC Pregnancy and Childbirth. 2018;18:156.
49. Ussher M, Lewis S, Aveyard P, et al. The London exercise and pregnant smokers (LEAP) trial: a randomised controlled trial of physical activity for smoking cessation in pregnancy with an economic evaluation. Health Technology Assessment. 2015;19:84.
50. Rubin DB. Multiple Imputation for Nonresponse in Surveys. New York, NY: Wiley; 1987.
51. Rubin DB. Discussion on multiple imputation. International Statistical Review. 2003;71:619-625.
52. Weiner BJ, Lewis CC, Stanick C, et al. Psychometric assessment of three newly developed implementation outcome measures. Implementation Science. 2017;12:108.

7.2 DATA MANAGEMENT PLAN

S:/Centre for Trials Research/Research/Mixed Studies/LISTEN/8. Data Management/8.1 Data Management inc DMP/DMP

7.3 TRIAL MASTER FILE AND STATISTICAL MASTER FILE

S:/Centre for Trials Research/Research/Mixed Studies/LISTEN

S:/Centre for Trials Research/Research/Mixed Studies/LISTEN/8. Data Management/8.5 Statistics

7.4 OTHER SOPS OR GUIDANCE DOCUMENTS

Not applicable
