# Supplementary material for: Effectiveness of a personalised self-management intervention for people living with long covid (Listen trial): pragmatic, multicentre, parallel group, randomised controlled trial
Source: BMJ Med. 2025 Jan 31;4(1):e001068. doi: 10.1136/bmjmed-2024-001068 (PMC11881025; doi:10.1136/bmjmed-2024-001068)
Supplement: Supplementary file 5 [file bmjmed-2024-001068.reviewer_comments.pdf]

PEER REVIEW HISTORY

BMJ Medicine publishes all reviews undertaken for accepted manuscripts. Reviewers are asked to complete a checklist review form and are provided with free text boxes to elaborate on their assessment. These free text comments are reproduced below.

This paper was submitted to another journal from BMJ but declined for publication following peer review. The authors addressed the reviewers' comments and submitted the revised paper to BMJ Medicine. The paper was subsequently accepted for publication at BMJ Medicine.

ARTICLE DETAILS

**TITLE (PROVISIONAL)**	Effectiveness of a Personalised Self-management Intervention for People Living with Long Covid: the LISTEN randomised controlled trial
**AUTHORS**	Busse, Monica; Pallmann, Philip; Riaz, Muhammad; Potter, Claire; Leggat, Fiona; Harris, Shaun; Longman, Andrea; Lowe, Rachel; Edwards, Adrian; Siriwardena, Aloysius; Sevdalis, Nick; McRae, Jackie; Fish, Jessica; Sewell, Bernadette; Jones, Fiona

VERSION 1 - REVIEW

**REVIEWER NAME**	McGregor, Gordon
**REVIEWER AFFILIATION**	UHCW NHS Trust
**REVIEWER CONFLICT OF INTEREST**	None
**DATE REVIEW RETURNED**	15-Aug-2024

**GENERAL COMMENTS**	Thank you for the opportunity to review this very important study which provides much needed evidence in people with non-hospitalised long covid. This was a large rigorously conducted RCT. A few issues require further justification:<br><br>Reporting 3 month outcomes only is not a sufficiently long enough follow-up to claim overall effectiveness. Whilst the effect of the intervention is likely to be greatest at 3 months, thus important to report, it is only longer-term follow up (12 months) that can confirm 'effectiveness'. Is longer-term follow up planned? I can't see it in the protocol. If it is, study reporting should wait for that data. Whilst this is transparently exported as limitation, I don't think that mitigates the issue. Data interpretation and conclusions should be attenuated to reflect the short term outcome data.<br><br>I think the same also applies (possibly more so) for cost-effectiveness. Can this be determined with only 3-month data?<br><br>How were potential participants who were sent information by post/email/text identified?<br><br>What were the recruiting sites? Primary care?<br><br>Inclusion criteria stated a at least 1 long covid symptom for more than 12 weeks – what symptoms were included?<br><br>The intervention could be better described. Did the 6 sessions have

	specific topics? How were they structured? What was the content? The underlying theory is well described but that actual content is not clear.<br><br>The authors should perhaps attest to the fact the effect of the intervention could in fact be diluted as some participants may have had only 1 symptom that may not have been amendable to self-management i.e. anosmia. Also, usual care may have included structured rehabilitation if this was provided in long covid clinics.<br><br>Were any efforts made to accommodate those with poor digital literacy or no access to digital infrastructure?<br><br>Were those who completed <4 sessions handled differently in the analysis?<br><br>More explanation and justification is required with regards to the minimally important difference (MID) identified for the primary outcome which is used to calculate the sample size and assess clinical effectiveness.<br><br>The paper referenced by the authors (doi: 10.2147/PROM.S198619) is a short report which ultimately used data from n=19 to identify mean change/MID (described as 'slight improvement') and effect size for 'routine activities' domain score. The paper reports a within group change in score of 7.51 points (effect size 0.32) as being the MID.<br><br>Could the authors consider the following points when interpreting their findings:<br><br>1. 'slight improvement' was considered to be clinically meaningful. Is 'slight improvement' large enough to be meaningful?<br>2. In the source data, is n=19 large enough to calculate an MID?<br>3. The sample for LISTEN was calculated on a between groups difference effect size of 0.32, but the referenced paper indicates the effect size was calculated within group.<br>4. An increase within group (n=19) of 7.51 points over time was considered to be the MID for the source paper, but the LISTEN trial used a between groups value and a smaller number was considered meaningful (2.90 adjusted). Can this be justified?<br>5. The trial was statistically significant so confirmed a between groups difference in effect size of 0.32 but does this translate accordingly to the proposed MID?<br><br>When concluding 'effectiveness', the conclusion should reflect that the population was 75% female and 93% White as these numbers limit generalisability of effectiveness.

**REVIEWER NAME**	Davis, Mike
**REVIEWER AFFILIATION**	
**REVIEWER CONFLICT OF INTEREST**	None
**DATE REVIEW RETURNED**	15-Aug-2024

**GENERAL COMMENTS**	I am reviewing this paper as a non-clinically qualified patient with limited understanding of statistical methodology. The nature and

	scope of the report and its components put it well out of my comfort zone, and I am not sure if my review can contribute much.<br><br>Long COVID is clearly a somewhat unexpected problem for a significant number of people and one that has had a major impact on sufferers capacity to manage day-to-day life and to gain support to do this. The LISTEN project is a well labelled project that provided appropriate support and care to patients who struggled with the consequences of COVID in the absence of any simple clinical solution. The paper, therefore, is a useful contribution, if clarity as to next steps could be achieved, and appears to suggest that there is both patient experience value and economic value in providing a level of post covid support.<br><br>The paper and its appendices is long and complex and it is not clear how it would be presented in the journal.

**REVIEWER NAME**	Oliván-Blázquez, Bárbara
**REVIEWER AFFILIATION**	University of Zaragoza
**REVIEWER CONFLICT OF INTEREST**	None
**DATE REVIEW RETURNED**	15-Aug-2024

**GENERAL COMMENTS**	This is a very interesting and complete manuscript, that shows the results of effectiveness and cost-effectivenes of the LISTEN intervention for people suffering from long covid.<br>The introduction section is well-writen and has a good thread.<br>The methodology section should be completed in some aspect which I explain below:<br>- Explain more in deep the following incluison/exclusion criteria: loss of sense of smell or taste during the acute phase in the absence of any other identified cause (without any kind of positive SARS-COV2 test?); why are not elegibles those who were hospitalized during the acute phase of Covid-19?<br>- Could the authors specify how many participants were recruited in health centers and how many by other means (advertising)?-<br>- missing L in line 52, in the word mental (menta)<br>- For readers who are not familiar with the instruments, it would be interesting to explain them in more depth (number of items, score ranges, etc.) so that they can better understand the results.<br>- I understand the methodology in terms of the realistic clinical practice that has been developed, but it would be necessary to go deeper into the intervention that the participants of the control group have performed (it may be a waiting list in some patients, compared to others who have done rehabilitation? there are studies that demonstrate the effectiveness of certain rehabilitation therapies and it could be a bias if some participants have done them and others have not.<br>- Have normality tests been performed for the choice of statistics to be used and types of analysis to be performed?<br>In the results section is also well written and show correctly the results, but according to the flowchart there are 188 people (113+67+8) who did not progress to randomization, was any analysis done to see if there were significant differences in any variable with those who did participate?<br>It is very interesting the Implementation Measure and cost-effectiveness because it facilitates the real implementation.<br>In the discussion section, it is important to delve into both the

	strengths and limitations of the study. Could there have been any bias? Could the LISTEN intervention be more effective in people with higher health literacy, self-efficacy, or educational attainment?

VERSION 1 – AUTHOR RESPONSE

Reviewer 1.

Reporting 3-month outcomes only is not a sufficiently long enough follow-up to claim overall effectiveness. Whilst the effect of the intervention is likely to be greatest at 3 months, thus important to report, it is only longer-term follow up (12 months) that can confirm 'effectiveness'. Is longer-term follow up planned? I can't see it in the protocol. If it is, study reporting should wait for that data. Whilst this is transparently exported as limitation, I don't think that mitigates the issue. Data interpretation and conclusions should be attenuated to reflect the short-term outcome data.

Our trial was designed in response to our funder's requirements to deliver an intervention trial in 2 years, but some participants had been living with symptoms for 2 years or more and even a short-term impact was welcomed. Our process evaluation (reported elsewhere) is focussing on how some of the short-term changes could translate into longer term behaviour change but further follow-up data would help to prove/disprove this hypothesis. We have added detail on this aspect in the discussion.

I think the same also applies (possibly more so) for cost-effectiveness. Can this be determined with only 3-month data?

This is no longer applicable to this manuscript as the cost effectiveness aspect has been removed from the manuscript.

How were potential participants who were sent information by post/email/text identified?

Our primary recruitment method was self-referral. People who were on waiting lists for the Long Covid clinics were sent invitation letters but if interested they needed to complete the expression of interest form on the trial website. We also opened 30 primary care (GP practices) Participant Identification Centres in England and Wales. Participants were sent text messages if on screening of records there was indication that they attended for a GP consultation having had a positive Covid test and had ongoing post Covid symptoms which meant that they may have been eligible for the LISTEN trial.

What were the recruiting sites? Primary care?

We opened research sites across primary and secondary care (GP practices and Long Covid clinics). We have added details about recruiting sites to the manuscript.

Inclusion criteria stated a at least 1 long covid symptom for more than 12 weeks – what symptoms were included?

We gathered data on Long Covid symptoms lasting for 12 weeks or longer across 10 organ systems as defined by (Davis et al. 2021) who surveyed 3762 participants with confirmed (diagnostic/antibody positive; 1020) or suspected (diagnostic/antibody negative or untested; 2742) Covid-19, from 56 countries, with illness lasting over 28 days and onset prior to June 2020. These included cardiovascular symptoms, dermatological symptoms, gastrointestinal symptoms, These

included cardiovascular symptoms, dermatological symptoms, gastrointestinal symptoms, head/ears/eyes/nose or throat symptoms, immunological symptoms, musculoskeletal symptoms, pulmonary symptoms, reproductive symptoms, systemic symptoms (e.g. fatigue, fever, sweats, coldness), mood and emotion symptoms, cognitive dysfunction, headaches, memory issues, sensory issues, sleep issues, language and speech issues and smell or taste issues.

The intervention could be better described. Did the 6 sessions have specific topics? How were they structured? What was the content? The underlying theory is well described but that actual content is not clear.
LISTEN is a personalised intervention (rather than a structured, manualised intervention). As such specific topics and structure are individual to the participant guided by intervention core principles. The intervention development processes, and resultant intervention details are reported elsewhere(Jones et al. 2024). We have now addressed this further in the discussion.

The authors should perhaps attest to the fact the effect of the intervention could in fact be diluted as some participants may have had only 1 symptom that may not have been amendable to self-management i.e. anosmia. Also, usual care may have included structured rehabilitation if this was provided in long covid clinics.
The median number of long Covid symptoms reported at baseline was 12 (interquartile range 9-14). This suggests that those with only 1 symptom would form the minority of our trial population. Furthermore, we adjusted for the number of symptoms in the final regression model (Model b). In terms of whether symptoms are amenable to self-management or not, our intervention offered a personalised approach whereby individuals were supported to identify solutions for their own challenges, and we did not define whether specific symptoms seemed amenable to self-management or not, rather we listened to the participants and explored what mattered to them. Whilst usual care may have included structured rehabilitation if it were provided in long Covid clinics, we accounted for this variability of care in our pre-specified statistical analyses with the use of a mixed effect model with random effect for the study centre (cluster).

Were any efforts made to accommodate those with poor digital literacy or no access to digital infrastructure?
All our participant materials were developed with guidance from, Diversity and Ability, a social enterprise, led by and for disabled people who support groups to create inclusive cultures within their activities. The book was posted to all participants in hard copy and the option to receive the intervention by phone was provided. This is detailed in the manuscript.

Were those who completed <4 sessions handled differently in the analysis?
Our analyses were based on intention to treat (ITT). We also specified and conducted per protocol analyses which included all those participants who completed 50% or more of the sessions. This is now addressed in our manuscript. Also see response (d) above.

More explanation and justification is required with regards to the minimally important difference (MID) identified for the primary outcome which is used to calculate the sample size and assess clinical effectiveness.

The paper referenced by the authors (doi: 10.2147/PROM.S198619) is a short report which ultimately used data from n=19 to identify mean change/MID (described as 'slight improvement') and effect size for 'routine activities' domain score. The paper reports a within group change in score of 7.51 points (effect size 0.32) as being the MID.

Could the authors consider the following points when interpreting their findings:
1. 'slight improvement' was considered to be clinically meaningful. Is 'slight improvement' large enough to be meaningful?
2. In the source data, is n=19 large enough to calculate an MID?
3. The sample for LISTEN was calculated on a between groups difference effect size of 0.32, but the referenced paper indicates the effect size was calculated within group.
4. An increase within group (n=19) of 7.51 points over time was considered to be the MID for the source paper, but the LISTEN trial used a between groups value and a smaller number was considered meaningful (2.90 adjusted). Can this be justified?
5. The trial was statistically significant so confirmed a between groups difference in effect size of 0.32 but does this translate accordingly to the proposed MID?

The selection of a primary outcome that reflected the multiple aspects of participation which could be impacted on by the wide-ranging symptoms of Long Covid, whilst being valid for self-administration given the remote nature of trial delivery, was an important consideration for the trial team. The trial was designed, and funding secured in 2021, at a time when there was no consensus on core outcomes for Long Covid research. There is now, as of 2023, a recommended set of instruments for survival, recovery, and respiratory functioning, symptoms, and conditions but consensus has not been reached for any single instrument that assesses impact on daily life (Gorst et al. 2023). The Oxford Participation & Activities Questionnaire (Ox-PAQ) is a psychometrically sound and valid patient reported outcome measure developed for use in a range of health conditions. It is theoretically grounded in the World Health Organization International Classification of Function and intended for use in the meaningful evaluation of interventions aimed at promoting participation and activity. The original study which reported on the MID used an anchor-based method which is the optimal approach to establish clinically important differences (Devji et al. 2020). They recruited 373 individuals with chronic disease, followed up for 6 months and included 290 in the final analyses. Data for those individuals (n=19/290) who reported at least being slightly better were used to calculate the related MID and effect size being the smallest change that respondents perceive to be important. The authors of this initial validation study acknowledge the small sample size and associated lack of precision of the presented MID (Devji et al. 2020) but also recognise the value of this data in informing initial estimates for clinical trial sample size calculations. They recommend further research with larger samples to inform greater precision in the estimated MIDs and related effect sizes.

We agree with the reviewer that there are limitations that should be considered with respects the MID that we used as a guide to inform our sample size calculation, namely, (1) the data came from a preliminary study in a small sample, (2) it was developed to reflect within group change rather that for two group comparison, 3) the data came from a different population (i.e. we do not know what the MID in Long Covid would be) and (4) the control group was receiving usual care where some change would be expected. Given these limitations, we suggest that the statistically significant

reduction of nearly 3 points in the Ox-PAQ routine activities scale for the treatment group compared with usual care could be considered a clinically relevant effect. Whilst lacking power and not part of our pre-specified statistical analysis plan, we have conducted a post hoc mixed effect logistic regression analysis that shows that the odds of achieving a MID of 7.51 or higher are close to being significantly greater in those receiving the LISTEN trial intervention (see response (h) above). We have added to our discussion to reflect this point.

We have also reviewed the qualitative data from responders and whilst we are not presenting this specifically in the manuscript the exemplar quotes below to provide further support for the clinically relevant effect noted in our trial:

> *P294 – "We talked about adapting past activities or focussing on what you can do and trying to adjust, or finding things that you used to enjoy and finding a way that you could still enjoy them. So I used to love doing park runs and I knew that I was in no state to be able to do those, but on a couple of occasions I went and volunteered there, and that was a way of getting a little bit of my old self back."*

> *P322 – "I think a lot of things [have changed]. A great example, like pre- [LISTEN]sessions, my Fridays were, I could only describe them as frustrated Fridays, where I would just be sort of lying down on the sofa sort of not really having much motivation to do anything. Not going for walks like I can do now. So, it has had a significant impact on my functioning and on my work-life balance as well."*

Reviewer 2.
Long COVID is clearly a somewhat unexpected problem for a significant number of people and one that has had a major impact on sufferers' capacity to manage day-to-day life and to gain support to do this. The LISTEN project is a well labelled project that provided appropriate support and care to patients who struggled with the consequences of COVID in the absence of any simple clinical solution. The paper, therefore, is a useful contribution, if clarity as to next steps could be achieved, and appears to suggest that there is both patient experience value and economic value in providing a level of post covid support.

The paper and its appendices is long and complex, and it is not clear how it would be presented in the journal.
We have reviewed for clarity, added a figure to illustrate the primary outcome and added a short section in the discussion to try to explain a suggested way forward such that our data can be used to inform development of services to enhance patient experience.

Reviewer 3.
When concluding 'effectiveness', the conclusion should reflect that the population was 75% female and 93% White as these numbers limit generalisability of effectiveness.
We have added detail in the discussion in relation to this and the potential for ascertainment bias.

This is a very interesting and complete manuscript, that shows the results of effectiveness and cost -effectiveness of the LISTEN intervention for people suffering from long covid.
The introduction section is well-written and has a good thread.
Thank you.

The methodology section should be completed in some aspect which I explain below:
Explain more in deep the following inclusion/exclusion criteria: loss of sense of smell or taste during the acute phase in the absence of any other identified cause (without any kind of positive SARS-COV2 test?); why are not eligibles those who were hospitalized during the acute phase of Covid-19?
At the time of writing the protocol people with long Covid symptoms had not all had access to routine testing, and this meant many people would not be recruited to the trial. We excluded people who were hospitalised during the acute phase of Covid-19 as a requirement of the funder.

Could the authors specify how many participants were recruited in health centres and how many by other means (advertising)?
We asked those who completed the online expression of interest forms to indicate how they heard about the trial. Responses were as follows: advertisement in press (n=17); recruited by trial site either via letter/referral from healthcare practitioner (n=187); social media (n=216); and other (n=304). The most common reasons given for "other" were: (i) told about it by a friend or relative; (ii) long Covid groups; and (iii) staff emails/newsletters at hospitals/health boards.

missing L in line 52, in the word mental (menta)
Corrected

For readers who are not familiar with the instruments, it would be interesting to explain them in more depth (number of items, score ranges, etc.) so that they can better understand the results.
Specific information about outcome measures were provided in Supplementary materials (items, score ranges and direction of effects in Table S3).

I understand the methodology in terms of the realistic clinical practice that has been developed, but it would be necessary to go deeper into the intervention that the participants of the control group have performed (it may be a waiting list in some patients, compared to others who have done rehabilitation? there are studies that demonstrate the effectiveness of certain rehabilitation therapies and it could be a bias if some participants have done them and others have not.
We have added detail with respects usual care in the manuscript. At the time of protocol development and recruitment to the LISTEN trial there was no definitive evidence of effectiveness of any rehabilitation interventions and such usual care rehabilitation would typically follow NICE guidelines based on indirect evidence and expert opinion. Since the completion of this trial, the only published trial providing definitive evidence for rehabilitation in long Covid (for people who were hospitalised) is the REGAIN trial which we have cited in our discussion.

Have normality tests been performed for the choice of statistics to be used and types of analysis to be performed?

As stated in the statistical analysis plan which was provided as supplementary material, the distribution of the scores were examined using histograms or boxplots for assessing normality.

In the results section is also well written and show correctly the results, but according to the flowchart there are 188 people (113+67+8) who did not progress to randomization, was any analysis done to see if there were significant differences in any variable with those who did participate?

Potential participants completed the expression of interest form and only if eligible progressed to consenting and baseline data collection. As such, when individuals did not progress to randomisation, we did not gain informed consent for trial participation or to gather any further data which could be explored. Individuals did not progress for the following reasons: (1) hospitalised for covid; (2) duration of Long Covid symptoms less than 12 weeks; (3) unable to allocate to participating research site; (4) had not consulted with GP; (5) reported another life limiting condition; and (6) participating in another Covid trial. We have explained this in the manuscript.

It is very interesting the Implementation Measure and cost-effectiveness because it facilitates the real implementation.

Thank you.

In the discussion section, it is important to delve into both the strengths and limitations of the study. Could there have been any bias? Could the LISTEN intervention be more effective in people with higher health literacy, self-efficacy, or educational attainment?

We have expanded the discussion to reflect the editorial committee point about ascertainment bias and other limitations. Our analyses controlled for age, gender, ethnicity, employment status and number of long Covid symptoms but we did not examine further factors such as health literacy, self-efficacy or educational attainment. It is however important to note that the intervention itself was co-designed to focus on how people could gain control and stability of symptoms at a time when people described real helplessness/hopelessness and thus facilitating self-efficacy was explored as part of our intervention fidelity measure.

References.

Davis, Hannah E, Gina S Assaf, Lisa McCorkell, Hannah Wei, Ryan J Low, Yochai Re'em, Signe Redfield, Jared P Austin, and Athena Akrami. 2021. 'Characterizing long COVID in an international cohort: 7 months of symptoms and their impact', EClinicalMedicine, 38.

Devji, Tahira, Alonso Carrasco-Labra, Anila Qasim, Mark Phillips, Bradley C Johnston, Niveditha Devasenapathy, Dena Zeraatkar, Meha Bhatt, Xuejing Jin, and Romina Brignardello-Petersen. 2020. 'Evaluating the credibility of anchor based estimates of minimal important differences for patient reported outcomes: instrument development and reliability study', BMJ, 369.

Fu, Vivian. 2019. 'Taking Charge After Stroke: a novel, community-based intervention to improve the lives of people with stroke', Open Access Te Herenga Waka-Victoria University of Wellington.

Gorst, Sarah L, Nina Seylanova, Susanna R Dodd, Nicola L Harman, Margaret O'hara, Caroline B Terwee, Paula R Williamson, Dale M Needham, Daniel Munblit, and Timothy R Nicholson. 2023. 'Core outcome measurement instruments for use in clinical and research settings for adults with post-COVID-19 condition: an international Delphi consensus study', The Lancet Respiratory Medicine, 11: 1101-14.

Jones, F, A Domeny, J Fish, F Leggat, I Patel, J McRae, C Rowe, and ME Busse. 2024. 'Using co-design methods to develop new personalised support for people living with Long Covid: the 'LISTEN'intervention', Health Expectations.
Potter, Claire, Fiona Leggat, Rachel Lowe, Philip Pallmann, Muhammad Riaz, Christy Barlow, Adrian Edwards, Aloysius Niroshan Siriwardena, Nick Sevdalis, and Bernadette Sewell. 2023. 'Effectiveness and cost-effectiveness of a personalised self-management intervention for living with long COVID: protocol for the LISTEN randomised controlled trial', Trials, 24: 75.

VERSION 2 – REVIEW

**REVIEWER NAME**	McGregor, Gordon
**REVIEWER AFFILIATION**	
**REVIEWER CONFLICT OF INTEREST**	None
**DATE REVIEW RETURNED**	18-Sep-2024

**GENERAL COMMENTS**	Thank you for addressing my comments from the original BMJ review. All comments have been addressed satisfactorily.<br><br>An unfortunate outcome of the revisions is that the paper (particularly the discussion) is very long. Could the discussion be streamlined to improve readability, but without losing the very important additional context that has been provided?

**REVIEWER NAME**	Wade, Angie
**REVIEWER AFFILIATION**	UCL, GOS ICH
**REVIEWER CONFLICT OF INTEREST**	None
**DATE REVIEW RETURNED**	20-Sep-2024

**GENERAL COMMENTS**	The authors have addressed reviewer and editor comments. Regarding editorial comment (I), a plot showing the data points was envisaged. The plot provided adds no further information than that given in table 4 (means and sds at the two time points). Please would the authors replace with a scatterplot with baseline values on the x-axis and post values on the y-axis, using differing symbols/colours for intervention and control groups. Such a plot would allow readers to view the actual data points collected and better understand the study outcomes, including where there are outliers.

**REVIEWER NAME**	Oliván-Blázquez, Bárbara
**REVIEWER AFFILIATION**	
**REVIEWER CONFLICT OF INTEREST**	None
**DATE REVIEW RETURNED**	27-Sep-2024

**GENERAL COMMENTS**	The authors have improved the manuscript. I do not recommend any further changes or additions. From my point of view it is suitable for publication.

VERSION 2 – AUTHOR RESPONSE

Reviewer 1. *Could the discussion be streamlined to improve readability, but without losing the very important additional context that has been provided.*

We have revised and worked to make the discussion more streamlined as requested.

Reviewer 2. *Please would the authors replace with a scatterplot with baseline values on the x-axis and post values on the y-axis, using differing symbols/colours for intervention and control groups. Such a plot would allow readers to view the actual data points collected and better understand the study outcomes, including where there are outliers.*

Thank you for this suggestion. We have revised Figure 2 as requested. We believe that it better illustrates the greater change in the primary outcome reported by trial intervention group compared to usual care with points below the diagonal line representing improvements in participation as lower scores on RASS are better.

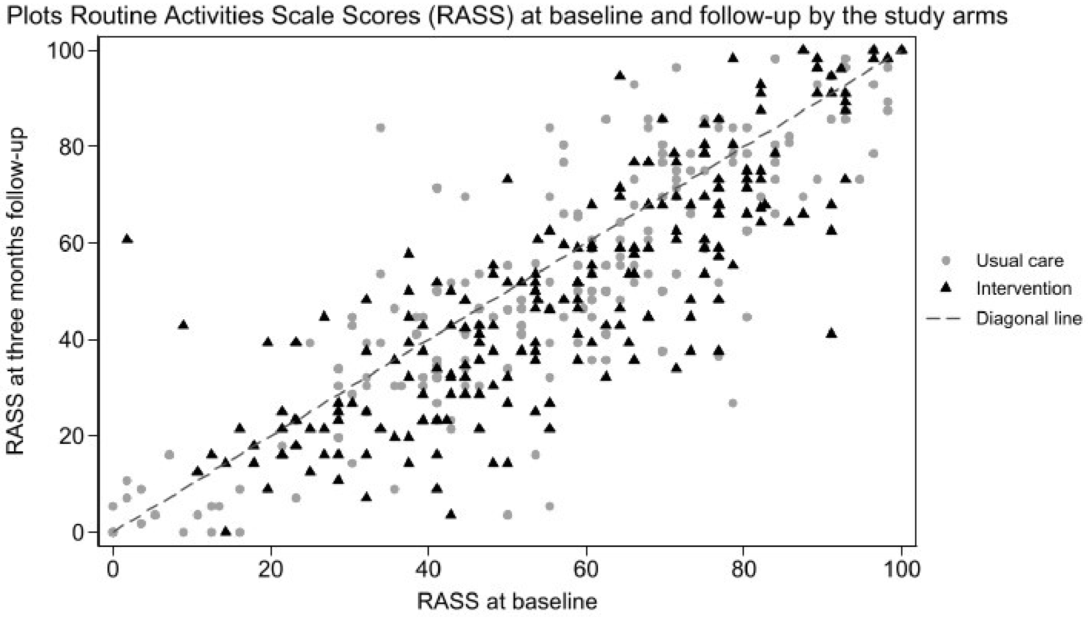

VERSION 3 – REVIEW

**REVIEWER NAME**	Islam, Nazrul
**REVIEWER AFFILIATION**	University of Southampton, School of Primary Care, Population Sciences and Medical Education
**REVIEWER CONFLICT OF INTEREST**	None
**DATE REVIEW RETURNED**	04-Nov-2024

**GENERAL COMMENTS**	The authors conducted a trial on an important and relevant topic. Certain aspects of the manuscript, including the interpretation,

	would benefit further clarification and/or modification.<br><br>Could the authors elaborate on the scale and range of the Ox-PAQ tool (is it 0-100?).<br><br>The sample size calculation clearly specifies as an MID of 7.51 on the raw score (0.32 SMD), which was not met. The study found a difference of -2.7 to -2.9 which is far away from the postulated MID. Therefore, the manuscript, including the abstract and the key message sections, should clearly mention that despite an indication of improvement, the change is not clinically meaningful.<br><br>Furthermore, there are at least three analyses (ITT, PP, and adjusted) for the primary outcome. Even though they returned comparable effect sizes, some of them crossed the null value of 0, while others did not. Just because some of them did not cross the null value should not necessarily mean that those findings are significant. We should rather focus on the precision of the estimates (aka, the 95% CIs are all wide, and an effect size of +0.02, or -0.15 could not be excluded). The upper bounds of the CIs are either very very small (-0.15), or on the harmful side (+0.02).<br><br>Even more, the analysis of odds of achieving changes of at least 7.51 (MID) showed a non-statistically significant OR.<br><br>There are many other potential biases including non-blinding, potential measurement bias etc., which authors<br><br>The loss to follow up was also much higher than the one postulated in the sample size calculation (15%).<br><br>The subgroup analyses by recruitment source show widely different findings (an overall effect size as low as -0.25 to -3.90).<br><br>All of these beg for a more nuanced and toned-down conclusions on the effectiveness of the intervention.

VERSION 3 – AUTHOR RESPONSE

The authors conducted a trial on an important and relevant topic. Certain aspects of the manuscript, including the interpretation, would benefit further clarification and/or modification.

Could the authors elaborate on the scale and range of the Ox-PAQ tool (is it 0-100?).

Details on the scale and range of Ox-PAQ and all other outcome measures were presented in detail in supplementary materials, however in the interests of clarity we have now additionally included this information in Table 4 in the main manuscript.

The sample size calculation clearly specifies as an MID of 7.51 on the raw score (0.32 SMD), which was not met. The study found a difference of -2.7 to -2.9 which is far away from the postulated MID. Therefore, the manuscript, including the abstract and the key message sections, should clearly mention that despite an indication of improvement, the change is not clinically meaningful.

We have amended the manuscript accordingly.

Furthermore, there are at least three analyses (ITT, PP, and adjusted) for the primary outcome. Even though they returned comparable effect sizes, some of them crossed the null value of 0, while others

did not. Just because some of them did not cross the null value should not necessarily mean that those findings are significant. We should rather focus on the precision of the estimates (aka, the 95% CIs are all wide, and an effect size of +0.02, or -0.15 could not be excluded). The upper bounds of the CIs are either very very small (-0.15), or on the harmful side (+0.02).

We have amended the manuscript to better reflect this uncertainty and shift the focus towards appraising the estimated effect sizes.

Even more, the analysis of odds of achieving changes of at least 7.51 (MID) showed a non-statistically significant OR.

We have discussed the issue of clinical meaningful change and the impact on firm conclusions at the beginning of the discussion.

There are many other potential biases including non-blinding, potential measurement bias etc.

We have in previous revisions addressed the potential biases (non-blinding, performance bias etc.) both in the discussion and in the abstract.

The loss to follow up was also much higher than the one postulated in the sample size calculation (15%).

We have added this to our discussion as another limitation of the study.

The subgroup analyses by recruitment source show widely different findings (an overall effect size as low as -0.25 to -3.90).

This additional analysis (post hoc) was requested by the clinical editor in the previous revision. As we explained in our response letter dated 15.10.2024, this is underpowered and cannot inform any firm conclusions. These results are not presented in the main manuscript.

All of these beg for a more nuanced and toned-down conclusions on the effectiveness of the intervention.

We have amended the abstract, key messages and discussion accordingly.
